# Dermatology “AI Babylon”: Cross-Language Evaluation of AI-Crafted Dermatology Descriptions

**DOI:** 10.3390/medicina62010227

**Published:** 2026-01-22

**Authors:** Emmanouil Karampinis, Christina-Marina Zoumpourli, Christina Kontogianni, Theofanis Arkoumanis, Dimitra Koumaki, Dimitrios Mantzaris, Konstantinos Filippakis, Maria-Myrto Papadopoulou, Melpomeni Theofili, Nkechi Anne Enechukwu, Nomtondo Amina Ouédraogo, Alexandros Katoulis, Efterpi Zafiriou, Dimitrios Sgouros

**Affiliations:** 1 Second Dermatology Department, School of Health Sciences, Aristotle University of Thessaloniki, 541 24 Thessaloniki, Greece; 2 Department of Dermatology, Faculty of Medicine, School of Health Sciences, University General Hospital of Larissa, University of Thessaly, 411 10 Larissa, Greece; 3 1st Department of Dermatology and Venereology, “Andreas Sygros” Hospital, Medical School, National and Kapodistrian University of Athens, 161 21 Athens, Greece; 4 Department of Internal Medicine, InnKlinikum Altötting, 84503 Altötting, Germany; 5 2nd Academic Department of General Surgery, Aretaieion Hospital, National and Kapodistrian University of Athens, 161 21 Athens, Greece; arkoumanisplasticsurgery@gmail.com; 6 Dermatology Department, University Hospital of Heraklion, 715 00 Heraklion, Greece; dkoumaki@yahoo.gr; 7 Computational Intelligence and Health Informatics Lab, Nursing Department, University of Thessaly, 382 21 Larissa, Greece; 8 Department of Internal Medicine, Chios General Hospital, 821 00 Chios, Greece; 9 Department of Internal Medicine, General Hospital of Karditsa, 431 31 Karditsa, Greece; myrto.papadopoulou4@gmail.com; 10 2nd Department of Dermatology and Venereology, “Attikon” General University Hospital, Medical School, National and Kapodistrian University of Athens, 157 84 Athens, Greecealexanderkatoulis@yahoo.co.uk (A.K.); disgo79@gmail.com (D.S.); 11 Department of Dermatology, Nnamdi Azikiwe University Teaching Hospital, Nnewi 431101, Nigeria; 12 Dermatology Department, University Joseph Ki-Zerbo, Ouagadougou 03 BP 7021, Burkina Faso

**Keywords:** artificial intelligence, dermatology, language, chatbot, AI-generated text, dermoscopy, terminology

## Abstract

*Background and Objectives*: Dermatology relies on a complex terminology encompassing lesion types, distribution patterns, colors, and specialized sites such as hair and nails, while dermoscopy adds an additional descriptive framework, making interpretation subjective and challenging. Our study aims to evaluate the ability of a chatbot (Gemini 2) to generate dermatology descriptions across multiple languages and image types, and to assess the influence of prompt language on readability, completeness, and terminology consistency. Our research is based on the concept that non-English prompts are not mere translations of the English prompts but are independently generated texts that reflect medical and dermatological knowledge learned from non-English material used in the chatbot’s training. *Materials and Methods*: Five macroscopic and five dermoscopic images of common skin lesions were used. Images were uploaded to Gemini 2 with language-specific prompts requesting short paragraphs describing visible features and possible diagnoses. A total of 2400 outputs were analyzed for readability using LIX score and CLEAR (comprehensiveness, accuracy, evidence-based content, appropriateness, and relevance) assessment, while terminology consistency was evaluated via SNOMED CT mapping across English, French, German, and Greek outputs. *Results*: English and French descriptions were found to be harder to read and more sophisticated, while SNOMED CT mapping revealed the largest terminology mismatch in German and the smallest in French. English texts and macroscopic images achieved the highest accuracy, completeness, and readability based on CLEAR assessment, whereas dermoscopic images and non-English texts presented greater challenges. *Conclusions*: Overall, partial terminology inconsistencies and cross-lingual variations highlighted that the language of the prompt plays a critical role in shaping AI-generated dermatology descriptions.

## 1. Introduction

Generative AI (GenAI) is one of the most widely accessible and commonly used forms of artificial intelligence available online, and it is accessible to virtually any user. This type of artificial intelligence has the ability to produce content, clarify complex concepts in almost every topic and domain, create well-organized texts, translate and summarize provided texts or even invent images that show the instructed picture [1]. Most existing evaluations of chatbot-crafted medical texts have been conducted in English, focusing on properties such as readability, structural coherence, and terminology use (Section 1.1). Building on these English-based findings, an important and largely unresolved question concerns how these linguistic properties are preserved or altered when a chatbot-generated text is produced in foreign (non-English) languages (Section 1.2).

### 1.1. The Linguistic Properties of a Chatbot-Crafted Text

Large language models (LLMs) are included in the category of GenAI and focus on the understanding and generation of human language [1]. These capabilities stem from powerful foundation models built using advanced machine learning and deep learning techniques such as transformer architecture, whose key component is the attention mechanism, based on which the model can identify which words in a sentence are the most important and how they relate to each other. As a result, transformers can understand context, identify and track relationships across long passages of text, and produce structured and meaningful responses [2,3]. The increasing volume of data that the models are trained on has led to modern systems trained on billions of data points and parameters, enabling them to capture more complex patterns in language and perform a wide range of text tasks with greater accuracy. Building on these LLMs, chatbots serve as the user-facing applications that provide interfaces, allowing users to interact with the model through text or voice [4]. For example, in the case of GPT-4 as an LLM, the corresponding chatbot is ChatGPT. The Generative Pre-Trained Transformer (GPT) family, including GPT-3 as a large language model (LLM) and GPT-4 as a large multimodal model (LMM), represents prominent examples of such foundation models [5].

GenAI outputs and texts can mimic human language; however, differences between AI-crafted and human-written texts can be detected—LLM-powered chatbots produce sophisticated and polished writing, while human-written text can vary in style, have a more personal perspective and a more empathic tone, particularly in storytelling and narration [6]. At the syntactic level, human-written texts may contain typos or irregular sentence structures, while chatbot-generated texts are typically free of such errors and often auto-corrected. Rahman and Watanobe described ChatGPT as “a revolutionary LLM that can maintain human-like conversations and generate human-like text for any natural language query that is nearly indistinguishable” [7]. Indeed, in most studies in which the participants were presented with a mixture of AI and non-AI texts, they struggled to find the text creator [8], while in a few studies, participants, especially those who use chatbots frequently and were therefore more familiar with them, were able to distinguish between the outputs when presented with a pair of answers [9]. It is worth mentioning that AI-written texts cannot be easily distinguished even with the use of AI-detector tools [10], raising concerns regarding the integrity of academic assessments and the potential misuse of AI for producing deceptive or unverified content. Although in most cases indistinguishable, the purpose of the text should also be examined separately and compared with human writing across different dimensions and in terms of how it affects the reader.

GPT models favor nominalizations (such as the expansion of contractions, removal of punctuations, etc.) as well as higher sentence complexity, giving a more technical or scientific tone to the text, while students often rely on modal and epistemic forms that reflect their personal attitude and confidence on the topic of interest [11]. Additionally, LLMs’ responses tend to stay strictly focused on the given question, providing objective, neutral, and informative answers, whereas human responses are more subjective and flexible, often shifting to related topics or interpreting the hidden meaning behind a question using common sense and prior knowledge. On the other hand, human writers produce richer and more specific content, frequently including citations, examples, humor, irony, metaphors, slang, or even memes, and express emotion through punctuation and grammar, while LLMs maintain a formal tone [12]. In a study that tested the production of fake news by both AI chatbots and writers, AI-generated fake news was often more emotionally provocative, employing attention-grabbing language, while frequently casting doubt without evidence and drawing unsupported conclusions [13]. Finally, chatbots tend to use specific types of nouns, adjectives, pronouns, and adjectival–prepositional modifiers [14].

Similar results were noted in the production of medical texts, with the tested chatbot outperforming nurse students in defining nursing goals and devising suitable interventions. Chatbot’s performance improved sequentially across diagnosing, goal setting, and intervention-writing, whereas students’ performance declined across these stages [15]. Due to this ability, chatbots have also been tested in generating empathetic responses to online patient inquiries and to aid in tasks involving medical knowledge. The results have shown that the integration of large language models into clinical workflows occurred naturally, proved practical, and was linked to better clinician well-being [16]. Although most studies on chatbots including specific medical topics show that it can effectively summarize existing knowledge and produce informative texts, the generation of fabricated references and hallucinations poses a significant concern for its use in academic and healthcare contexts [17].

A significant application of LMMs is their ability to process uploaded images for various tasks, such as modification or description. This image upload feature makes chatbots particularly valuable in dermatology, where users can submit macroscopic or dermoscopic images and request assessments. Indeed, LMMs can generate detailed images from textual descriptions, and conversely, they can analyze images and provide accurate answers. The model learns to create a shared “embedding space” where both text and images can be represented numerically, allowing it to understand relationships between words and visual elements and associate concepts with appearances. Additionally, with the application of transfer learning, knowledge based on massive datasets can be applied to a specific task with additional minimal training, giving the model the ability to recognize similar images. Noteworthy are the attention mechanisms, methods that allow the model to focus on the most relevant parts of the input image and let the model decide which words, image regions, or combinations are important for a particular task [18].

Regarding dermatology, LLMs are able to analyze dermatologic images by generating descriptions or differential diagnoses, and conversely, when provided with a textual description of a lesion, the chatbot can suggest potential diagnoses. In a recent study, the authors assessed how well a chatbot can understand dermoscopic terminology by presenting it with dermoscopic descriptions. Even among experts, dermoscopic language can be challenging, as it often incorporates descriptive or metaphorical expressions that may lead to subjective interpretations of the same image. The chatbot performed well, generating mostly comprehensive, diagnostically helpful, and educational lists of differential diagnoses. Slight modifications in how descriptions are phrased can cause the chatbot to generate similar differential diagnoses, since dermatology has a specialized vocabulary and synonyms are often interchangeable. For instance, in basal cell carcinoma, replacing “arborizing” with the term “branching” produces nearly the same differential list (such as BCC, SCC, melanoma). The same pattern appears when non-dermoscopic wording is altered. If dotted vessels in psoriasis are described as “scattered,” the chatbot still offers a similar set of possible diagnoses, such as psoriasis, lichen planus, and pityriasis rosea [19].

Therefore, wording plays a crucial role not only in how chatbots interpret dermatologic input, but also in the descriptive text they are instructed to generate and the descriptions they themselves produce.

### 1.2. Chatbot-Crafted Text in Different Languages

Due to the ability of LLMs to produce texts in different languages, they have been used as educational tools as they can edit and refine writing, translate content, offer synonyms and idiomatic expressions, and provide explanations when needed. In their study, Zhang et al. differentiated the vocabulary learning process into receptive, productive, and incidental. Productive vocabulary learning consists of words that learners are able to actively produce and apply in oral or written communication, while incidental vocabulary learning refers to the unintentional or non-deliberate acquisition of new words that occurs while learners are engaged in other tasks or activities. LMMs were found to improve both vocabulary processes, indicating their ability to bridge gaps between two or more languages [20]. The translation ability of ChatGPT has also been tested on medical texts, such as in radiology, with reports showing that the quality of translations between English French and Spanish outperformed that of the Russian language [21].

Therefore, the question of how well chatbots actually understand foreign languages arises. Research suggests that their responses are not simply English translations but newly generated sentences and texts, because the model understands the language directly rather than relying on English or synonym-based dictionaries [22]. This concept is based on the fact that LLMs perform according to the training on the language in question. As a result, variations in training may lead to different levels of language proficiency. For example, ChatGPT is only able to recognize approximately 80% of the words in the dictionary and 90% of the words in *Don Quixote*, in some cases assigning incorrect meanings [23]. This variation may affect text generation across multiple domains, including medicine and, consequently, dermatology [21].

GPT-4 currently supports over 50 languages [24]. As demonstrated in Table 1, LLMs trained primarily on the language of the prompt perform better, while in certain cases, they did not understand all the questions provided and generated inaccurate or nonsensical sentences [25]. Research shows that ChatGPT, which is primarily trained on English-centric internet data, can pass the United States Medical Licensing Examination and effectively handle a variety of clinical medical queries [26]. However, in some non-English medical exams, it fails to reach a passing score [27]. The most frequent conclusion drawn across studies is that the tested generative AI models generally perform worse in foreign languages than in English, despite the performance being rated as sufficient.

Table 1 shows 17 studies comparing English and foreign language outputs in medical texts; LLMs consistently perform better in English than in other languages when applied to medical examinations, patient counseling, and clinical question-answering tasks. The results are uneven, but the best performance outside English is reported in high-resource languages (such as Chinese, Spanish, Italian, French). In contrast, Arabic outputs, for example, showed more limitations, including lower correctness and reduced completeness, while in certain cases, the performance of the models had domain-specific weaknesses.

It is worth mentioning that the language component is also part of the METRICS checklist (Model, Evaluation, Timing, Range/Randomization, Individual factors, Count, and Specificity of prompts and language) [44], which aims to standardize the reporting of generative AI-based studies in healthcare. Also, the texts created are usually assessed by participants using a Likert scale, usually the CLEAR tool, that assesses five components as follows: completeness, lack of false information (accuracy), evidence-based content, appropriateness, and relevance. Using the METRICS checklist and CLEAR tool is a common approach used in studies. Therefore, language becomes a measurable component of performance, as AI models are highly sensitive to how prompts are worded.

As shown in Table 1, no studies have evaluated the comprehensiveness of chatbots’ responses in dermatology across languages other than English, nor compared their performance between languages. To address this gap, we conducted a study presenting various dermatology images (both macroscopic and dermoscopic) to a chatbot, prompting it to generate descriptions in non-English languages, and subsequently comparing these outputs to assess the chatbot’s effectiveness in this medical domain.

## 2. Materials and Methods

**Figure 1:**
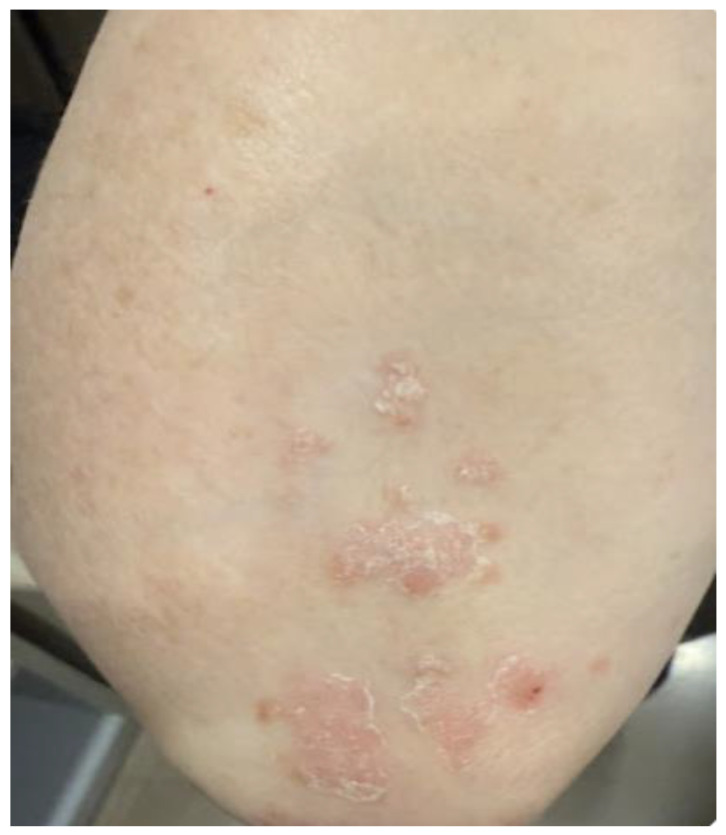
Example image uploaded to the chatbot for generating dermatology descriptions. The Supplementary Materials provides the six prompts and corresponding outputs in five different languages.

**Figure 2:**
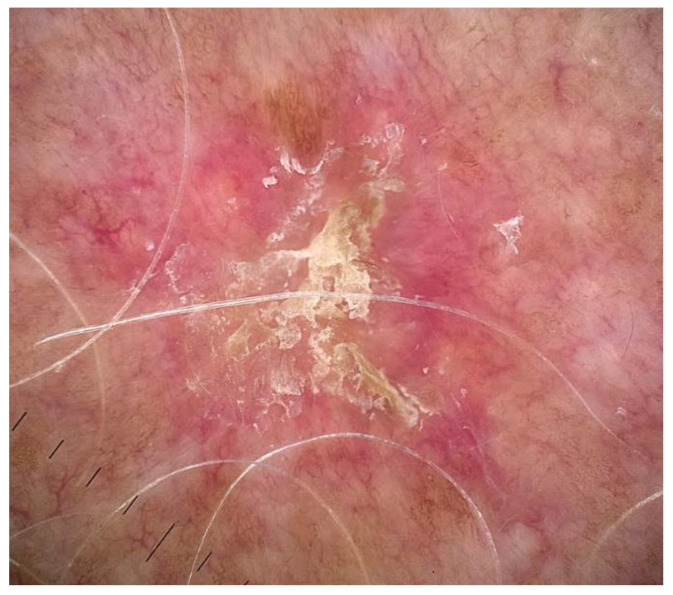
Dermoscopy image of actinic keratosis uploaded to the chatbot to generate a description and possible diagnosis. The Supplementary Materials provides the chatbot-generated descriptions.

In order to assess the chatbot’s ability to produce dermatology-related texts in different languages, we provided 5 macroscopic and 5 dermoscopic images of commonly encountered skin lesions. In the first category, we included images with psoriasis plaques, herpes labialis, facial dermatosis, actinic keratosis scalp and onychomycosis, while the second category featured dermoscopy images of actinic keratosis, dermatofibroma, seborrheic keratosis, dysplastic nevi, and pigmented BCC. For methodological consistency, we focused on the above-mentioned representative selection of common macroscopic and dermoscopic lesions that have well-established visual descriptors, minimizing confounding effects from diagnostic uncertainty that could influence the linguistic features of the AI-produced descriptions. Those images were uploaded in the Gemini 2 chatbot with the following prompts: “Describe what you observe in this clinical image in one short paragraph, focusing on the visible dermatological features (color, texture, distribution, and possible diagnosis)” for the macroscopy image and “Describe what you observe in this dermoscopic image in one short paragraph, focusing on the visible dermoscopic structures (colors, patterns, borders, and possible diagnosis)” for the dermoscopy image. The prompts were translated using Google Translate, so the prompt was provided in 4 additional languages apart from English (Table 2). The same image was uploaded 6 times to collect 6 outputs for each language in three different times of the day (morning, afternoon and evening), always starting a ‘new chat’. As a result, 300 output descriptions of dermatology images were collected for assessment. Examples of a macroscopy image and a dermoscopy image can be seen in Figure 1 and Figure 2 and their respective descriptions in Table 3 and Table 4.

In order to evaluate those descriptions, we examined the readability of each description, the clinical concept similarity amongst the different language answers, and the assessment of the image–text correlation using another chatbot. Also, the outputs were assessed using the CLEAR tool by native speakers dermatologists with proficient knowledge of the English language, allowing direct comparisons to be made.

Readability was assessed using the Lix index to evaluate the comprehensibility of online materials across multiple languages. The prompts were analyzed with the Lix readability metric, a validated tool that measures text complexity based on two factors: average sentence length and the proportion of long words (defined as having more than six characters). The Lix score is calculated by dividing the total number of words by the number of sentences and adding the percentage of long words. Higher Lix scores indicate more complex and difficult texts [45]. Lix, although mainly applied for Scandinavian languages, has been shown to be a reliable readability measure in several languages, including English, French, German, Italian, and Spanish [46]. Each description text was processed using the Lix calculator available at https://haubergs.com/rix (accessed on 1 December 2025) [47].

To assess clinical concept similarity amongst the different language answers, we used Systematized Medical Nomenclature for Medicine–Clinical Terminology (SNOMED CT), which is a medical terminology library. For every description provided by the chatbot for every language, the text was translated to English using Google Translate. Following this, the clinical concepts detected (lesion, symptom, disease) were collected and compared in each language group. According to SNOMED CT terminology, every clinical term has a unique Concept ID (similar to a CUI), and each concept can have multiple fully specified terms in different languages. The percentages of mismatch were then calculated and compared across the texts.

Image correlation was also assessed using a different chatbot that was trained on different data. As the outputs assessed were crafted by Gemini 2, we provided prompt pairs to ChatGPT-4 asking to what percentage the descriptions provided were about the same dermatology image. The question used for ChatGPT-4 was “Do these two descriptions refer to the same image? Answer with yes, maybe, or no.” As a result, 36 pairs for each image description were created to evaluate not only the relationships between English and non-English languages but also between languages such as French and Greek, etc.

For each language, ten native-speaker dermatology experts, with strong English proficiency, independently evaluated the 60 responses associated with each language using a 3-point Likert scale. Responses were scored as follows: 3 for excellent answers, 2 for mediocre or partially correct answers, and 1 for poor or incorrect answers. Each text was assessed based on CLEAR criteria reported previously. METRICS presentation of our study is shown in Table 3.

## 3. Results

To evaluate the linguistic quality of the chatbot-generated dermatology descriptions, we conducted an analysis encompassing several complementary assessment approaches. Specifically, we examined the readability of each description (Section 3.1), the similarity and consistency of clinical concepts expressed across different language outputs using SNOMED CT-mapping (Section 3.2), and the correspondence between image content and textual descriptions using an independent chatbot-based evaluation (Section 3.3). In addition, all generated outputs were assessed using the CLEAR tool by native-speaker dermatologists with proficient knowledge of the English language, enabling direct and systematic comparisons across languages (Section 3.4).

### 3.1. Readability Scores Amongst Languages

In Table 4 and Table 5, we present the Lix score index, sentence number, word number and words per sentence regarding disease-specific macroscopy case examples, dermoscopy descriptions, and summary points for each language, respectively. As presented, the readability score presents variability that is connected to the language structure as well as to the skin lesions that the chatbot describes. For example, the text reporting the description of psoriasis plaques is simpler than the one describing the dermoscopic features of BCC. Regarding the comparison between macroscopy and dermoscopy language, no statistic differences were observed between the same language, except from English (61.63 ± 6.75 vs. 69.30 ± 5.45, *p* < 0.001). This result may come from the fact that dermoscopy uses a highly specialized, abstract vocabulary to describe microscopic–level structures as well as a more technical terminology with pattern-based abstractions and a metaphorical terminology that occasionally confuses the reader.

As shown in Table 3 and Table 4, a statistically significant difference was observed in the language-based descriptions, with ANOVA reporting *p* = 0.004. Post hoc analysis revealed significant differences specifically between French and English, and between French and German for macroscopic descriptions. This observation suggests that the chatbot tends to write descriptions in French language that were either more complex or simpler in structure than their English and German counterparts. For dermoscopic descriptions, ANOVA also indicated a significant difference (*p* < 0.01), and post hoc comparisons identified statistically significant differences between Greek and French, Greek and English, and English and German (Table 5). As a whole (including both dermoscopic and macroscopic language), English (65.4 ± 7.8) and French (65.7 ± 6.6) were proven to be the most difficult texts provided (ANOVA test, *p* < 0.01)). French descriptions often employ long adjectival phrases and post-nominal modifiers, such as “plaques rouges recouvertes de squames argentées,” which increase average word length and lexical density. The descriptions in English tend to use long, multi-clause sentences with ideas connected by commas and conjunctions. In contrast, Greek and German descriptions were easier to read. Greek texts, although using long words, tended to have more linear sentence structures. Finally, German texts, while containing compound nouns, often broke information into shorter or more segmented clauses, making the sentences easier to analyze.

### 3.2. Medical Terminology-Based Assessment of Chatbot-Created Dermatology Descriptions and Their Comparison in Multiple Language

For each language, we analyzed the dermatology descriptions and, in the case of macroscopic examination, identified clinical terms related to primary and differential diagnoses, morphology, anatomical sites, surface characteristics, colors, distributions, and additional findings. We then mapped these terms to their corresponding SNOMED CT IDs (international edition) (see example table for psoriasis descriptions in English text outputs). For dermoscopy, we evaluated core inflammatory features, structural epidermal changes such as scaling, crusting, and hyperkeratosis, vascular patterns, and lesion architecture (Table 6).

The descriptions in French, Greek, and German were translated using Google Translate, after which we extracted the key clinical terms and compared them to the English reference descriptions. Mismatch percentages were calculated as Mismatch % = Number of missing or extra or different terms compared to English reference description/Total reference terms × 100) with the language-specific ranges presented in Table 3.

The largest mismatch was observed in German, while the smallest was in French, likely because French dermatology extensively uses Latin-derived terms, which closely resemble international English medical terminology. Similar evaluations of English and French translations in prior studies also reported high concordance (Table 7) [48].

Limitations of this approach include translation accuracy and SNOMED concept coverage. Translation quality can vary significantly by language when mapped into English, depending on the domain and methodology [49]. Additionally, SNOMED CT often lacks pre-coordinated concept codes for many specific dermoscopic features (such as “radial streaks,” “pseudonetwork,” “regression structures”) used in dermoscopy pattern analysis, as dermoscopic terminology is a specialized imaging vocabulary not fully represented in the core SNOMED CT content.

It is worth mentioning that the different focus on specific image characteristics appears to vary with language. For example, in the case of actinic case dermoscopy, a feature-based comparison emphasizing skin cancer-related features was performed between the reference dermoscopic description and three datasets from French, Greek, and German, using clinically relevant dermoscopic and diagnostic features aligned with SNOMED CT terminology. Features were grouped into inflammatory, epidermal structural, vascular, architectural, oncologic, and autoimmune-specific categories, with higher weights assigned to vascular and oncologic indicators due to their greater diagnostic significance and divergence for autoimmune skin disease clues. Overall, all three description datasets demonstrated overlap with the reference pattern, confirming a shared underlying clinic–dermoscopic spectrum characterized by diffuse erythema, inflammation, poorly defined lesion borders, scaling, crusting, and non-melanocytic morphology. It is worth mentioning that the chatbot’s differential diagnoses, reporting the six outputs as a whole, were consistent across all studied languages, indicating that the relationships between dermoscopic or macroscopic features, clinical differentials, and language were preserved. Further studies specifically designed to evaluate language-dependent diagnostic performance across a broader range of lesions are needed.

In the oncology-weighted approach, French-translated texts most closely reflected the reference English dermoscopic phenotype (with weighted match score of 87.5%). Greek-translated represented a more malignant-leaning variant within the same spectrum (weighted match score of 82.5%), while German-translated texts showed partial overlap with a shift toward autoimmune inflammatory pathology (weighted match of 70%). The respective overall matches without performing special weighting for the specific example were 87.5%, 83%, and 75%, respectively. Notably, this example demonstrates that clinical terminology concept similarity remains directionally consistent even when oncologic traits are emphasized, which is particularly important in dermoscopic analysis.

### 3.3. Chatbot-by-Chatbot Assessment of Multiple-Language Dermatology Descriptions

In this section, we evaluated multiple language comparisons to determine whether AI-generated descriptions produced by one chatbot are interpreted consistently by another chatbot. We used Gemini-2 to generate image descriptions and then prompted ChatGPT-4.5 in two different languages, asking whether each description represented the same image, offering responses “Yes”, “No”, or “Maybe”. The results are summarized in the table.

Overall, the most frequent response was “Yes”, while the “Maybe” responses generally represented that the overall image presentation was similar, certain descriptive elements were either missing, emphasized differently, or presented with a slightly different differential diagnosis. Our results indicate that in both multi-language approaches and individual language comparisons, dermoscopic descriptions showed more variability than macroscopic descriptions (Chi-square, *p* = 0). Dermoscopy produced more “Maybe” responses, suggesting that users uploading dermoscopic images to the chatbots and asking for information may need to ask multiple times to obtain a reliable answer (Table 8).

Specific image characteristics also influenced results. For example, in dermoscopy images of seborrheic keratosis, all language comparisons reported a full “Yes” category, indicating that this skin disease presentation was consistent across languages. Beyond the image itself, prompt-specific differences were observed: certain prompts produced alternative diagnoses or emphasized different features compared to other prompts for the same image, as indicated in the actinic keratosis example presented in Table 9. These prompt-related variations were more prominent in dermoscopy cases than in macroscopic cases.

Regarding language comparisons, the largest uncertainty expressed with highest “Maybe” or “No” answers in macroscopic descriptions occurred when comparing the French–German pair (Chi-square, *p* = 0), whereas in dermoscopy, the greatest uncertainty was observed for the French–Greek and Greek–German pairs (Chi-square, *p* = 0).

It is important to note certain limitations in terms of comparing languages based on chatbot-generated descriptions assessed by another chatbot. One limitation is the probabilistic nature of chatbot outputs, which can lead to variability in the content, a feature common to all studies that rely on chatbot assessments and mitigated with increased number of outputs. Additionally, because the descriptions were produced by one chatbot and evaluated by another, any inherent biases, text styles, or errors of the generating model can affect the distribution of responses that were produced by the assessing model. As a result, the observed differences may reflect characteristics of the generating chatbot rather than the intrinsic properties of the languages themselves.

### 3.4. CLEAR Tool-Based Assessment of the Chatbot’s Dermatology Descriptions

To evaluate differences in image description quality across languages and image types, a mixed-design (split-plot) ANOVA was performed. The dependent variable was the Likert scale score, ranging from 1 to 3, for each dimension of interest (completeness, accuracy, evidence-based content, appropriateness, and relevance—CLEAR assessment). The analysis included one between-subjects factor—language (four levels: English, French, Greek, German per 10 participants per language group)—and one within-subjects factor—image type (two levels: macroscopy, dermoscopy)—as all participants rated both image types. The outputs assessed were 600 in each language (six prompts produced based on five macroscopy images and six prompts produced based on five macroscopy images, all assessed by native-speaker dermatologists). This design allowed testing for (i) the main effect of language, (ii) the main effect of image type, and (iii) the interaction between language and image type. Statistical significance was evaluated at *p* = 0.05.

A series of split-plot ANOVAs were conducted to examine the effects of language and image type using CLEAR parameters. For comprehensiveness and evidence-based approach, significant main effects of language and image type were observed, while the language × image type interaction was non-significant (*p* = 0.34 for comprehensiveness and *p* = 0.25 for evidence-based approach). This indicates that the effect of language on accuracy does not depend on the image type. For accuracy, appropriateness, and relevance, significant main effects of language and image type were observed, along with a significant interaction. Overall, these results demonstrate that language and image type both influence performance metrics, with some effects acting independently and others interacting depending on the outcome measured (Table 10).

Across all evaluated metrics, English consistently achieved the highest scores (Table 10), followed by German, French, and Greek. For all languages, responses to macroscopic images were higher than those for dermoscopic images, indicating that macroscopy was easier to interpret, and more complete, accurate, and appropriate outputs were produced by the chatbot in question. The largest declines in performance for dermoscopy were observed in Greek and French, particularly for accuracy and evidence-based content, highlighting a language and image type interaction. Overall, these results demonstrate that both language and image type influence performance, with English and macroscopic images producing the best outcomes, while dermoscopy and non-English languages pose greater challenges across all evaluated metrics.

## 4. Discussion

Dermatology is a medical domain that relies on a highly complex terminology that encompasses descriptions of lesion types, distribution patterns, colors and color intensities, as well as involvement of specialized sites such as hair and nails, making interpretation subjective. This complexity is also based on the use of dermoscopy, a noninvasive magnified imaging technique frequently used to support diagnosis, which introduces an additional descriptive framework. Even among dermatologists, dermoscopic terminology can be challenging, as it often incorporates descriptive and metaphorical language, resulting in variability in image interpretation. Moreover, the diagnostic value of dermoscopic images may differ depending on the available equipment and the level of expertise of the observer. Consequently, dermoscopy represents a particularly demanding domain for evaluation when integrating AI technologies such as LLMs that connect image with texting. Furthermore, the breadth of dermatologic conditions (inflammatory dermatoses, skin cancer, skin infections, etc.) and the diversity of visual presentations add another layer of complexity. These challenges are further compounded in multilingual contexts, where differences in language and translation may alter interpretation and meaning [50].

It is generally highlighted that the lack of standardized requirements for delivering non-English medical care includes clinician preferences for support such as interpreters, varying levels of linguistic fluency and comfort, and the languages spoken by the patient populations served. AI ha thes potential to bridge this linguistic gap. Different language texts can have different levels of complexity, and it is generally not easy to assess readability across languages. Generally, the most common readability assessment of English texts is Flesch Reading Ease, Gunning Fog, Flesch–Kincaid Grade Level, Coleman–Liau Index, SMOG Index, and Automated Readability Index (ARI). Based on these reading assessment scores, a previous study assessing ChatGPT-generated content across dermatologic conditions, found that the material corresponded to a 10th-grade reading level [51]. In another study, the authors produced revised health information regarding dermatology, such as sunscreen instructions, at a lower reading level consistent with American Medical Association recommendations (10th- to 15th-grade reading levels) [52]. Therefore, evaluating readability across multiple metrics is essential when assessing texts generated by chatbots, as it directly affects their accessibility and usefulness to end users. It is also worth noting that some languages have language-specific readability assessment tools, such as the Ateşman readability index for Turkish [53]. More broadly, evaluating readability across different languages presents inherent challenges, as readability metrics are often language-dependent and may not be directly comparable across linguistic contexts. The LIX readability index was selected in our study due to its relative language independence and established use across multiple European languages. As it relies on sentence length and word length rather than syllable structure, LIX is particularly suitable for cross-linguistic readability assessment of technical and medical texts [46]. The language differences we found, with English and French providing more complex and sophisticated texts than Greek and German, indicate that word length alone does not determine readability, as sentence structure, clause organization, and modifier placement are equally important factors. These insights are critical, as structural differences can impact comprehension even when terminology is standardized. Consequently, users are encouraged to experiment with multiple languages when requesting highlights or explanations for dermatology images, particularly if the terminology is unclear. Alternatively, users can prompt the chatbot to simplify the text and reduce readability difficulty.

SNOMED CT is a comprehensive, multi-hierarchical clinical terminology system that provides a standardized and scientifically validated terminology list representing clinical information. It has been widely employed in research and studies focusing on medical terminology, clinical documentation, and electronic health records, enabling consistent representation of clinical concepts across different healthcare settings. In case of chatbot’s text, a recent scoping review highlights diverse approaches to integrating SNOMED CT into language models to support biomedical natural language understanding and generation [54]. Other studies have applied NLP techniques to annotate free medical text with SNOMED CT codes and to map local medical terminologies into SNOMED CT using generative AI. Together, these efforts suggest the feasibility of incorporating SNOMED CT into the evaluation or annotation of chatbot-generated medical content for consistency [55]. Our data indicate that SNOMED CT mapping mismatches vary by language and image type in the chatbot-created descriptions. For macroscopy, mismatches were generally low in French (0–15%) but more variable in Greek and German (0–40%), suggesting that basic terminology for macroscopic descriptions is relatively well-aligned in French but less consistently mapped in the other languages. For dermoscopy, mismatches were notably higher across all languages. This suggests that dermoscopic terminology is more complex, specialized, and harder to standardize, leading to greater discrepancies when mapping to SNOMED CT. Overall, these findings highlight language-specific challenges in clinical terminology standardization in chatbot-created texts. Using specific diagnosis category such as oncology-weighted approach, the overall clinical concept similarity remained generally consistent across languages, showing that the outputs preserve the main focus. This approach is helpful in dermoscopy because it highlights subtle differences in emphasis, which can be important when assessing features related to malignancy.

Following this, we used a cross-AI evaluation of semantic consistency across languages, providing a practical method to test whether AI-generated clinical text retains its meaning when interpreted by a separate model. Specifically, ChatGPT-4.5 was used to assess the outputs generated by another chatbot (Gemini-2) and was prompted to judge whether each pair of different language description represented the same image, responding with “Yes,” “Maybe,” or “No.” For dermoscopy, agreement was lower, with 789 “Yes,” 271 “Maybe,” and 20 “No” responses. The higher number of “Maybe” and “No” responses suggests that dermoscopic descriptions, in this case as well, are proved to be more complex and subject to interpretation, likely due to more specialized terminology and subtle visual features. Certain language pairs, such as French–Greek and Greek–German, showed more uncertainty or disagreement, highlighting language-specific challenges in preserving nuanced clinical meaning. Overall, these findings indicate that while AI-generated descriptions are largely interpretable across languages, dermoscopy is more prone to inconsistencies, emphasizing the need for careful review when translating or cross-validating specialized clinical image descriptions.

The CLEAR tool is widely used in the literature to evaluate medical text readability and quality, enabling comparisons between different chatbots or between human- and AI-generated content. To our knowledge, this is the first study to use CLEAR to compare chatbot-generated medical content across multiple languages, allowing us to evaluate cross-linguistic consistency, interpretability, and quality in a multilingual context. Consistent with previous studies, English texts generally achieved the highest readability and quality scores (Table 1). Using the CLEAR tool, dermoscopic image descriptions consistently scored lower than macroscopic descriptions across all languages.

Lastly, apart from the limitations connected to each assessment method described in Section 2, it is important to note that these findings are specific to the chatbot in question. Also, another limitation of this study is the exclusion of rare skin diseases, which often lack standardized macroscopic and dermoscopic descriptors. As AI technology continues to evolve, more sophisticated chatbots or different model versions may produce different results. A key observation from our study is that the language used in the prompt significantly influences the terminology and descriptions generated, even when describing the same image. Therefore, future studies evaluating chatbot-generated content should report the language of the instructions to ensure reproducibility and proper interpretation of results.

## 5. Conclusions

This study demonstrates that AI-generated dermatology texts are influenced by both language, such as English, French, German and/or Greek, and image type (macroscopy and/or dermoscopy). Differences in readability scores were detected amongst languages. English and macroscopic images produced the most accurate, complete, and readable outputs based on CLEAR assessment, while non-English languages and dermoscopic images posed greater challenges, Furthermore, partial terminology inconsistency amongst different languages, as evaluated using SNOMED CT and cross-lingual comparisons, indicates that the language used in prompts plays a critical role in shaping AI-generated dermatology texts. Limitations include the fact that the findings are specific to the chatbot model evaluated and that rare skin diseases were not included due to limited standardized descriptors. Evaluating the reliability and consistency of chatbot-generated dermatology descriptions and their associated diagnoses in multilingual settings is essential, as this line of research may contribute to the development of standardized, multilingual dermatologic terminology specifically designed for safe and effective chatbot use in dermatology.

## Figures and Tables

**Table 1:** Studies comparing chatbot efficacy, particularly ChatGPT, in generating medically relevant responses across multiple languages.

Study	Foreign Language	Comparison	Result
[27]	Chinese	Comparison between LLMs trained on different languages corpora, based on Chinese National Medical Licensing Examination as reference point.	LLMs that are mostly trained on English texts and those trained primarily on Chinese text both perform well on the examination, although the Chinese-trained models achieve slightly better results.
[28]	Finish	Assessment of ChatGPT and Microsoft Bing exam performance on Finish medical exams.	LLMs did not pass the test.
[29]	Chinese	Evaluation of the performance of ChatGPT China National Medical Licensing Examination.	When used without additional training, ChatGPT did not achieve a passing score on the exam. However, when combined with external medical knowledge frameworks such as Knowledge and Few-Shot Enhancement In-Context Learning, LLMs of different sizes showed consistent and substantial performance improvements.
[30]	Japanese	Assessment of the accuracy of large language models (LLMs) in generating responses to questions in clinical radiology practice based on Japan Radiology Board Examination.	GPT-4 scored 65% when answering Japanese questions. The performance of GPT-4 was also domain-based as the model significantly better in nuclear medicine than in diagnostic radiology. GPT-4 also performed better on lower-order questions than on higher-order questions.
[31]	French and Arabic	Testing of ChatGPT in a set of neuroscience questions.	The richness of ChatGPT’s response and the intelligibility of its writing in Arabic and French languages were notably inferior to that in English.
[32]	Arabic	Comparison of AI models’ efficiency for infectious disease queries in English and Arabic.	Queries in English consistently achieved the highest performance, with Bard performing best, followed by Bing, ChatGPT-4, and ChatGPT-3.5. A similar pattern appeared in Arabic queries, though the differences were not statistically significant. A generally inferior performance of the tested generative AI models was observed for Arabic compared to English, despite being rated “above average”.
[33]	Arabic	Comparison of AI model efficiency for cirrhosis in English and Arabic.	The performance of ChatGPT in Arabic was less accurate than that in English.
[34]	French, Italian Spanish	Medical note assessment in French, Italian, English, and Spanish.	The results of the model evaluation study showed that ChatGPT-4 is accurate when analyzing medical notes in three different languages.
[35]	Spanish	Evaluation of chatbots Spanish responses on cancer questions.	AI chatbots generated good-quality Spanish responses.
[36]	Spanish	Completeness and readability of ChatGPT-4 discharge instructions for common pediatric emergency room complaints in Spanish.	GPT-4 generated discharge instructions in both English and Spanish that were easy to read and adjusted the reading level appropriately. The English instructions generally demonstrated greater completeness compared to the Spanish versions.
[37]	Spanish	Evaluation ChatGPT’s answers to ten questions on labor epidurals in Spanish and English.	ChatGPT’s responses in Spanish were less accurate than its answers in English, especially concerning the impact of labor epidurals on the progression of labor and delivery method.
[38]	Italian	Comparison of the accuracy and completeness of English vs. Italian answers on metabolic dysfunction-associated steatotic liver disease (MASLD).	While language does not seem to impact ChatGPT’s ability to deliver clear and thorough counseling to MASLD patients, its accuracy is still inadequate in some areas.
[39]	French	Comparison of differential diagnoses based on English and French prompts including retina cases.	GPT-4 performed similarly in English and French while ophthalmic images were identified in both languages as critical for correct diagnosis.
[40]	English, French, Chinese, Thai, Hindi, Nepali, Vietnamese, and Arabic	Comparison of seven AI chatbot LLMs on three simple cancer-related queries across eight languages.	Hallucinations are less frequent in English.
[41]	French	Assessment of effectiveness of ChatGPT responses on kidney donation in different languages.	Nephrologists showed moderate agreement for English responses and poor agreement for French responses. Kidney donors exhibited high agreement for English but low for French.
[42]	Arabic	Comparison of ophthalmology queries in Arabic vs. English based on multiple AI models, including ChatGPT and DeepSeek.	English remains the higher-performing language overall. Arabic outputs were slightly lower across all models.
[43]	Arabic	Comparison of the performance of ChatGPT-4 and Gemini in answering virology multiple-choice questions in both English and Arabic, and evaluation of the quality of the content they generate.	ChatGPT-4 and Gemini showed stronger performance in English than in Arabic, with ChatGPT-4 consistently outperforming Gemini in both accuracy and completeness.

**Table 2:** Multiple-language inputs used to instruct chatbots to describe macroscopic or dermoscopic dermatology images.

Language	Prompt Requesting a Macroscopic Description of the Dermatology Image	Prompt Requesting a Dermoscopic Description of the Dermatology Image
English	Describe what you observe in this clinical image in one short paragraph, focusing on the visible dermatological features (color, texture, distribution, and possible dιagnοsis).	Describe what you observe in this dermoscopic image in one short paragraph, focusing on the visible dermoscopic structures (colors, patterns, borders, and possible diagnosis).
French	Décrivez en un court paragraphe ce que vous observez sur cette image clinique, en vous concentrant sur les caractéristiques dermatologiques visibles (couleur, texture, distribution et diagnostic possible).	Décrivez en un court paragraphe ce que vous observez sur cette image dermoscopique, en vous concentrant sur les structures dermoscopiques visibles (couleurs, motifs, contours et diagnostic possible).
German	Beschreiben Sie in einem kurzen Absatz, was Sie auf diesem klinischen Bild beobachten, und konzentrieren Sie sich dabei auf die sichtbaren dermatologischen Merkmale (Farbe, Textur, Verteilung und mögliche Diagnose).	Beschreiben Sie in einem kurzen Absatz, was Sie auf diesem dermoskopischen Bild beobachten, und konzentrieren Sie sich dabei auf die sichtbaren dermoskopischen Strukturen (Farben, Muster, Grenzen und mögliche Diagnose).
Greek	Περιγράψτε τι παρατηρείτε σε αυτήν την κλινική εικόνα σε μία σύντομη παράγραφο, εστιάζοντας στα ορατά δερματολογικά χαρακτηριστικά (χρώμα, υφή, κατανομή και πιθανή διάγνωση).	Περιγράψτε τι παρατηρείτε σε αυτήν την δερματοσκοπική εικόνα σε μία σύντομη παράγραφο, εστιάζοντας στις ορατές δερματοσκοπικές δομές (χρώματα, μοτίβα, όρια και πιθανή διάγνωση).

**Table 3:** METRICS checklist used in this study.

METRICS Parameters	METRICS Results
Model	Gemini 2.0
Evaluation	Language differences in creating content in dermatology.
Timing	The survey performed from 1 September 2025 to 15 December 2025.
Range/Randomization	Diversity of images provided and randomness in test cases or prompts.
Individual factors	Dermatology and dermoscopy knowledge of the participants; personal opinions.
Count	60 prompts for each language and 30 prompts for each image.
Specificity of prompts	Reading difficulty assessment, use of similar clinical terms, CLEAR criteria amongst the prompts.
Language	English, German, French, Greek.

**Table 4:** Mean ± SD of linguistic assessment scores for each language evaluated.

Macroscopy Language	Index
English	61.67 ± 7.16
French	66.23 ± 6.44
Greek	62.3 ± 6.12
German	60.5 ± 5.38
Dermoscopy Language	
English	69.13 ± 6.63
French	65.2 ± 6.81
Greek	60.53 ± 4.77
German	61.53 ± 5.31

**Table 5:** Post hoc statistical comparisons of linguistic assessment scores across languages.

	Dermoscopy Language—Post Hoc *p* Value Comparison	Macroscopy Language—Post Hoc *p* Value Comparison
Greek vs. French	0.02	0.08
Greek vs. English	<0.01	0.98
Greek vs. German	0.9	0.69
French vs. English	0.06	0.03
French vs. German	0.09	<0.01
English vs. German	<0.01	0.89

**Table 6:** SNOMED CT concept IDs for clinical terms identified in English-language chatbot prompts.

Category	Clinical Term	SNOMED CT Concept ID
Diagnosis	Plaque psoriasis	200965009
Morphology	Plaque (skin lesion)	1522000
	Erythematous plaque	72768000
	Papule	25694009
	Indurated/raised lesion	260399008
Surface/Texture	Hyperkeratosis	26996000
	Scaly skin	271761007
	Thickened skin	263899003
Color/Inflammation	Erythema	247441003
Anatomical Location	Extensor surface of limb	249973009
	Elbow	127949000
Distribution Pattern	Localized lesion	255471002
	Grouped/clustered lesions	255504006

**Table 7:** Mismatch ranges based on SNOMED CT concept IDs of clinical terms identified in each language’s chatbot prompts.

	Mismatch Percentage Range in French Language	Mismatch Percentage Range in Greek Language	Mismatch Percentage Range in German Language
Macroscopy	0–15%	0–40%	0–40%
Dermoscopy	12.5–55.6%	16.7–50%	5–60%

**Table 8:** Number of “Yes,” “Maybe,” and “No” responses generated by ChatGPT 4.5 for each language comparison.

Macroscopy	Yes	Maybe	No
English–French comparisons	148	32	0
English–Greek comparisons	159	21	0
English–German comparisons	174	6	0
French–Greek comparisons	174	6	0
Greek–German comparisons	169	11	0
French–German comparisons	123	57	0
	947	133	0
Dermoscopy	Yes	Maybe	No
English–French comparisons	129	45	6
English–Greek comparisons	144	36	0
English–German comparisons	139	41	0
French–Greek comparisons	118	62	0
Greek–German comparisons	119	47	14
French–German comparisons	140	40	0
	789	271	20

**Table 9:** Similarity between six English and six German dermoscopic descriptions of actinic keratosis. Responses were obtained using ChatGPT 4.5 to assess whether descriptions in English vs. German languages report the same image. “Yes” indicates essentially the same lesion features, “Maybe” indicates partial or interpretative overlap, and “No” indicates a clear mismatch (none observed in this dataset). English prompts 3–6 align most closely with German 1–6, whereas English 1–2 and 5 show more partial overlap due to their emphasis on potential malignancy versus inflammatory interpretation. Also, the fifth German output emphasizes the differential of regressive LPLK, while fibrotic structures were reported, which is not explicitly mentioned in most of the compared English descriptions. Therefore, prompt-specific variations occur.

	German Prompt 1	German Prompt 2	German Prompt 3	German Prompt 4	German Prompt 5	German Prompt 6
English Prompt 1	Yes	Maybe	Maybe	Maybe	Maybe	Maybe
English Prompt 2	Maybe	Yes	Maybe	Maybe	Maybe	Maybe
English Prompt 3	Yes	Yes	Yes	Yes	Maybe	Yes
English Prompt 4	Maybe	Maybe	Maybe	Maybe	Maybe	Yes
English Prompt 5	Yes	Yes	Yes	Yes	Maybe	Maybe
English Prompt 6	Yes	Yes	Yes	Yes	Maybe	Maybe

**Table 10:** CLEAR assessment scores for each parameter of the chatbot-generated dermatology descriptions.

		Completeness	Lack of False Information (Accuracy)	Evidence-Based Content	Appropriateness	Relevance
English	Macroscopy	2.5 ± 0.70	2.49 ± 0.76	2.47 ± 0.72	2.51 ± 0.70	2.53 ± 0.71
	Dermoscopy	2.37 ± 0.76	2.22 ± 0.81	2.16 ± 0.83	2.21 ± 0.81	2.16 ± 0.84
	Sum	2.44 ± 0.72	2.35 ± 0.74	2.31 ± 0.76	2.36 ± 0.74	2.34 ± 0.76
French	Macroscopy	2.33 ± 0.70	2.22 ± 0.78	2.34 ± 0.74	2.23 ± 0.79	2.11 ± 0.82
	Dermoscopy	2.18 ± 0.22	2.03 ± 0.84	2.09 ± 0.8	2.01 ± 0.78	2.01 ± 0.79
	Sum	2.23 ± 0.77	2.19 ± 0.79	2.17 ± 0.78	2.12 ± 0.79	2.06 ± 0.81
Greek	Macroscopy	2.38 ± 0.75	2.2 ± 0.70	2.31 ± 0.81	2.2 ± 0.7	2.15 ± 0.85
	Dermoscopy	2.13 ± 0.87	1.92 ± 0.87	2.1 ± 0.81	2.04 ± 0.80	2.06 ± 0.82
	Sum	2.26 ± 0.77	2.06 ± 0.84	2.21 ± 0.82	2.12 ± 0.81	2.10 ± 0.84
German	Macroscopy	2.35 ± 0.70	2.25 ± 0.79	2.30 ± 0.76	2.2 ± 0.79	2.2 ± 0.79
	Dermoscopy	2.2 ± 0.77	2.11 ± 0.77	2.08 ± 0.79	2.15 ± 0.96	2.16 ± 0.79
	Sum	2.28 ± 0.79	2.18± 0.78	2.19 ± 0.78	2.18 ± 0.87	2.18 ± 0.78

## Data Availability

The data described in this study are available upon request from the corresponding author.

## Supplementary Materials

The following supporting information can be downloaded at https://www.mdpi.com/article/10.3390/medicina62010227/s1, Full version of the study questionnaire.
