# Supplementary material for: Dermatology “AI Babylon”: Cross-Language Evaluation of AI-Crafted Dermatology Descriptions
Source: Medicina (Kaunas). 2026 Jan 22;62(1):227. doi: 10.3390/medicina62010227 (PMC12843771; doi:10.3390/medicina62010227)
Supplement: Supplementary file 1 [file medicina-62-00227-s001.zip › medicina-4087922-supplementary.pdf]

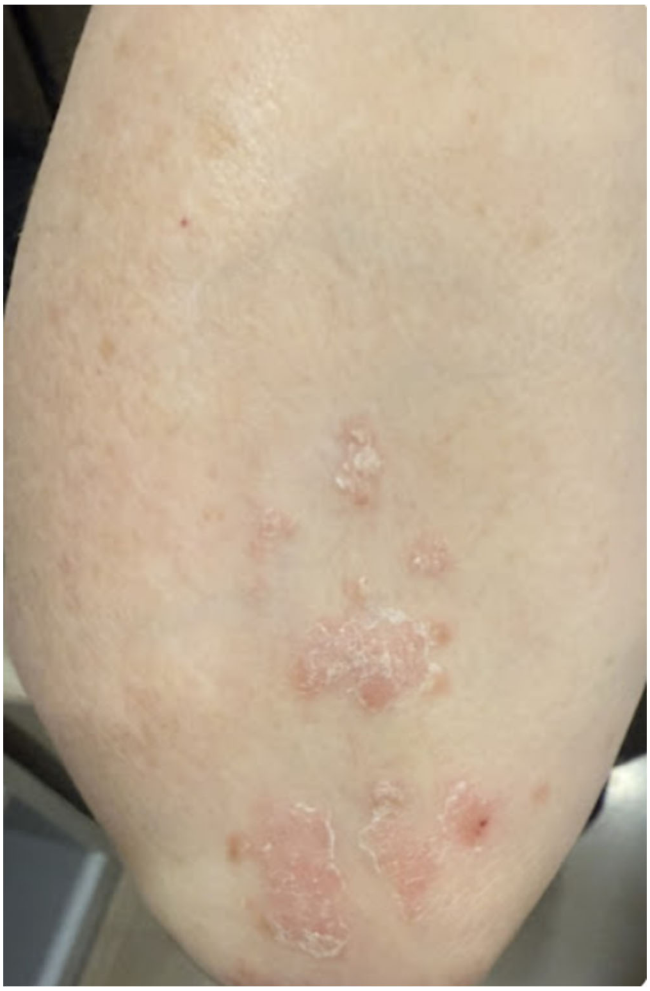

Description	Completeness	Lack of false information (accuracy)	Evidence-based content	Appropriateness	Relevance
**1**This clinical image displays several erythematous (reddish) to pink plaques with varying degrees of overlying silvery-white scale on the skin, likely located on an extensor surface such as the elbow. The lesions appear well-demarcated and exhibit a slightly raised/indurated texture. This presentation, particularly the scaling on an extensor surface, is highly suggestive of plaque psoriasis.					
**2**This clinical image shows several erythematous (reddish) plaques with overlying silvery-white scale on the skin, likely located on an extensor surface (like an elbow or knee) given the curvature and common presentation site. The lesions are well-demarcated and appear to be coalescing in some areas, characteristic features strongly suggestive of plaque psoriasis. Some smaller, satellite lesions are also visible, and the surrounding skin appears normal.					

**3**This clinical image displays several erythematous (reddish) to salmon-pink plaques located on the extensor surface of the elbow, a common site. These lesions are well-demarcated and covered with characteristic silvery-white scale, giving them a scaly and thickened texture. The distribution shown is localized to the elbow area. Based on these classic features—erythematous plaques with micaceous scale on an extensor surface—the morphology is highly suggestive of plaque psoriasis.					
**4**<br>Based on the image, the skin shows several well-demarcated erythematous (red/pink) plaques covered with silvery-white scale, indicative of hyperkeratosis. These lesions vary in size, with some appearing to be small papules and others coalescing into larger patches. The texture is rough and scaly. The distribution appears to be localized on the extensor surface of a limb, likely the elbow, a classic location for certain skin conditions. These features are highly suggestive of a possible diagnosis of plaque psoriasis.					
**5** This clinical image displays an area of skin, likely over an elbow, exhibiting several erythematous (reddish) plaques with overlying silvery-white scale. The lesions vary in size, with some appearing small and papular, and others forming larger, irregular plaques. The texture is scaly and somewhat raised above the surrounding normal skin. The distribution appears localized and clustered on the extensor surface of the limb. These dermatological features, particularly the well-demarcated, scaly, erythematous plaques on an extensor surface, are highly suggestive of Psoriasis.					
6This clinical image displays several pink-to-red plaques on the skin, notably over what appears to be an extensor surface (like an elbow). The lesions are characterized by a scaly, silvery-white surface and appear to be well-demarcated from the surrounding normal skin. They range in size and are somewhat coalescing in the center of the affected area. The presence of these erythematous plaques with thick silvery-white scale on an extensor surface is highly suggestive of a common dermatological condition such as plaque psoriasis.					
1L'image présente une zone cutanée (semblant être un coude ou une autre zone articulaire) affichant plusieurs plaques érythémateuses (rouges) de tailles variées, bien délimitées et recouvertes d'une desquamation argentée ou blanc-nacrée (squames). Ces lésions ont une texture écailleuse et épaissie (hyperkératosique) et sont regroupées, formant une distribution en plaques. Cette apparence clinique, notamment les plaques rouges					

recouvertes de squames argentées sur une zone d'extension (comme le coude), est hautement suggestive du diagnostic de psoriasis en plaques (ou psoriasis vulgaire).					
2 L'image présente une zone de peau (probablement un coude ou une autre zone articulaire) où l'on observe plusieurs plaques érythémateuses (rouges ou rosées) de taille variable. Ces lésions sont caractérisées par une desquamation visible, se présentant sous forme de squames blanchâtres ou argentées qui leur confèrent une texture rugueuse et écailleuse. Les bords des plaques semblent bien délimités. La distribution est localisée, et l'aspect général — en particulier les plaques rouges avec de grosses squames blanches sur une zone typique d'extension articulaire — est très suggestif d'un possible psoriasis en plaques. On note également de petites papules discrètes et quelques petites excoriations ou points rouges isolés.					
3 Ceci est une image clinique montrant des lésions cutanées sur l'articulation du coude (ou une zone similaire). On observe plusieurs plaques érythémateuses (rouges) de tailles variées, bien délimitées, recouvertes de squames argentées ou blanchâtres épaisses, conférant une texture rugueuse et écailleuse. Ces lésions sont regroupées sur la face externe de l'articulation, une distribution typique. Les caractéristiques principales (plaques érythémateuses et squameuses, distribution sur les surfaces d'extension) suggèrent fortement un diagnostic de psoriasis en plaques.					
4<br>C'est une image clinique montrant des lésions cutanées localisées sur ce qui semble être le coude ou une autre surface d'extension. Les lésions se présentent sous forme de plaques érythémateuses (rougeâtres) avec des squames épaisses et blanchâtres-argentées en surface, donnant un aspect sec et écailleux. La distribution est en regroupement (confluentes ou juxtaposées). Ces caractéristiques cliniques sont hautement suggestives de Psoriasis en plaques (ou Psoriasis vulgaire), une dermatose inflammatoire chronique courante affectant souvent les surfaces d'extension comme les coudes et les genoux.					
5<br>En examinant cette image clinique, on observe une zone de peau présentant des plaques érythémateuses (rouges) de taille variable, localisées dans la région du coude ou d'une articulation similaire. Ces plaques sont caractérisées par une desquamation (squames) blanchâtre ou argentée visible à leur surface, ce qui leur confère une texture rugueuse ou squameuse. La distribution est groupée en plusieurs lésions distinctes ou					

confluentes. Ces caractéristiques dermatologiques (plaques érythémateuses et squameuses, couleur argentée/blanchâtre, localisation sur le coude) sont fortement suggestives d'un diagnostic de Psoriasis en plaques.					
6<br>L'image présente une zone de peau qui semble être l'intérieur ou l'extérieur d'un membre (possiblement le coude ou le genou), avec des lésions cutanées bien définies. On observe la présence de plaques érythémato-squameuses, c'est-à-dire des zones de peau rouges (érythème) recouvertes de squames blanchâtres et épaisses. Les lésions sont de tailles et de formes variées, certaines étant confluentes pour former de plus grandes plaques, d'autres étant des papules plus petites. La texture est sèche et écailleuse (squameuse), et l'érythème sous-jacent est visible, allant du rose pâle au rouge. La distribution semble localisée. Ces caractéristiques cliniques, notamment les plaques rouges recouvertes de squames argentées/blanchâtres, sont hautement suggestives d'un diagnostic de Psoriasis en plaques (Psoriasis Vulgaris), en particulier si elles sont situées sur les faces d'extension comme le coude ou le genou, bien que d'autres diagnostics comme un eczéma chronique puissent être considérés.					
1 Η εικόνα απεικονίζει μια περιοχή δέρματος, πιθανώς στον αγκώνα ή σε άλλη εκτατική επιφάνεια, η οποία παρουσιάζει πολλαπλές, ερυθηματώδεις πλάκες με έντονη λευκοργυρώδη απολέπιση (λέπια). Οι πλάκες φαίνονται καλά περιγεγραμμένες και έχουν ανομοιόμορφη κατανομή στην περιοχή, με μικρότερες βλάβες να συνυπάρχουν με μεγαλύτερες. Η υφή των βλαβών είναι τραχιά λόγω της υπερκεράτωσης (απολέπισης). Αυτά τα χαρακτηριστικά - καλά περιγεγραμμένες, ερυθηματώδεις πλάκες με παχιά, αργυρόχρωμη απολέπιση σε εκτατικές επιφάνειες - είναι πολύ ενδεικτικά της Ψωρίασης Πλάκας.					
2 Η εικόνα απεικονίζει μια περιοχή δέρματος, πιθανώς σε αγκώνα ή άλλη εκτατική επιφάνεια, με πολλαπλές, ερυθηματώδεις πλάκες με σαφώς καθορισμένα όρια. Τα ορατά δερματολογικά χαρακτηριστικά περιλαμβάνουν την παρουσία ασημί-λευκών λεπιών στην επιφάνεια των βλαβών, υποδεικνύοντας υπερκεράτωση και απολέπιση. Το υποκείμενο δέρμα φαίνεται να είναι ελαφρώς ερυθρό (ερύθημα). Οι βλάβες ποικίλλουν σε μέγεθος και εμφανίζονται συρρέουσες σε ορισμένα σημεία, ενώ άλλες είναι μικρότερες, διάσπαρτες βλατίδες. Αυτή η εμφάνιση, χαρακτηριζόμενη από ερυθηματώδεις πλάκες με παχιά λέπια σε εκτατική επιφάνεια, είναι ενδεικτική					

δερματικών παθήσεων όπως η Ψωρίαση (ιδιαίτερα η Ψωρίαση Πλάκας), αν και απαιτείται κλινική αξιολόγηση για επιβεβαίωση.					
3 Στην κλινική εικόνα παρατηρούνται ερυθηματώδεις πλάκες με σαφή όρια, οι οποίες καλύπτονται από αργυρόχροες, λεπιδώδεις απολεπίσεις (λέπια). Η υφή τους φαίνεται τραχιά και η κατανομή τους είναι περιορισμένη, εντοπιζόμενη σε μία συγκεκριμένη περιοχή του δέρματος που μοιάζει να είναι ο αγκώνας ή το γόνατο, τυπική θέση εντοπισμού. Το χρώμα του υποκείμενου δέρματος είναι ελαφρώς ερυθρό/ροζ. Τα χαρακτηριστικά αυτά είναι ιδιαίτερα ενδεικτικά ψωρίασης, πιθανώς ήπιας έως μέτριας μορφής, αν και η τελική διάγνωση απαιτεί πάντα ιατρική αξιολόγηση.					
4 Η εικόνα απεικονίζει μια περιοχή δέρματος, πιθανώς στον αγκώνα ή σε άλλη εκτατική επιφάνεια, όπου παρατηρούνται ερυθηματώδεις πλάκες με έντονη λέπιση. Το χρώμα των βλαβών είναι κόκκινο-ροζ (ερύθημα), ενώ η υφή χαρακτηρίζεται από αργυρόχροες, παχιές φολίδες (λέπια) στην επιφάνειά τους, οι οποίες φαίνονται να αποκολλώνται. Οι βλάβες είναι σαφώς αφοριζόμενες από το γύρω, φυσιολογικό δέρμα και η κατανομή τους φαίνεται να είναι εστιάζουσα σε αυτήν την περιοχή. Τα ορατά δερματολογικά χαρακτηριστικά, ειδικά η παρουσία καλά αφοριζόμενων ερυθηματωδών πλακών με παχιά λέπια σε εκτατική επιφάνεια, είναι ενδεικτικά της ψωρίασης.					
5 Η εικόνα απεικονίζει μια περιοχή δέρματος, πιθανώς στον αγκώνα ή σε άλλη εκτατική επιφάνεια, όπου παρατηρούνται ερυθηματώδεις πλάκες με έντονη λέπιση. Οι βλάβες είναι ασαφώς ή σαφώς περιγεγραμμένες και έχουν ένα ασημένιο-λευκό χρώμα λέπια, χαρακτηριστικό που υποδηλώνει μια υπερκερατωσική διαδικασία. Η κατανομή φαίνεται να είναι περιορισμένη και εστιασμένη στην συγκεκριμένη περιοχή. Τα δερματολογικά αυτά χαρακτηριστικά είναι συμβατά με την εικόνα της Ψωρίασης, μιας χρόνιας αυτοάνοσης φλεγμονώδους νόσου του δέρματος, αν και μια οριστική διάγνωση απαιτεί πάντα ιατρική εξέταση.					
6 Από την εικόνα, παρατηρούνται στο δέρμα, πιθανώς στην περιοχή του αγκώνα, ερυθροχρωματισμένες πλάκες με σαφή όρια, που καλύπτονται από αργυρόχροα, λεπιδώδη λέπια. Η υφή των βλαβών φαίνεται τραχιά και ξεφλουδισμένη. Η κατανομή είναι εστιομένη και συρρέουσα σε ορισμένα σημεία. Αυτά τα χαρακτηριστικά είναι ιδιαίτερα ενδεικτικά της ψωρίασης, μιας χρόνιας φλεγμονώδους δερματοπάθειας, με την ψωρίαση κατά πλάκας να είναι ο πιο κοινός τύπος, συχνά εντοπιζόμενος στους αγκώνες και τα γόνατα.					
1					

Die Aufnahme zeigt eine Nahansicht der Haut, wahrscheinlich an einem Streckbereich wie dem Ellenbogen, mit mehreren fleckförmigen, rötlich-rosa Läsionen, die von silbrig-weißen, schuppigen Auflagerungen bedeckt sind. Die Hautveränderungen sind als Plaques erhöht und haben eine scharfe Begrenzung zu der umgebenden, normal erscheinenden Haut. Die Verteilung ist fleckförmig/gruppiert. Das klinische Erscheinungsbild, insbesondere die charakteristischen rötlichen Plaques mit groben, silbrigen Schuppen an einer typischen Prädilektionsstelle, ist hochverdächtig für eine Psoriasis vulgaris (Schuppenflechte).					
2 Auf dem Bild, das eine Hautpartie zeigt, vermutlich einen Ellbogen aufgrund der Form und der typischen Lokalisation von Läsionen an Streckseiten, sind erythematöse (rötliche) Plaques sichtbar. Diese Läsionen sind scharf begrenzt und weisen eine auffällige silbrig-weiße, schuppende Oberfläche auf, was der Textur eines hyperkeratotischen und schuppigen Belags entspricht. Die Verteilung ist konzentriert auf einen Bereich, der typisch für chronische Reibung oder Druckstellen ist. Die Kombination aus scharf begrenztem Erythem und den charakteristischen silbrigen Schuppen ist stark verdächtig auf eine Diagnose von Psoriasis (Schuppenflechte), insbesondere der Plaque-Psoriasis, der häufigsten Form dieser chronisch-entzündlichen Hauterkrankung.					
3<br>Basierend auf dem klinischen Bild sieht man am Ellenbogengelenk mehrere ** erythematöse Papeln und Plaques** mit einer deutlichen, silbrig-weißen Schuppung auf der Oberfläche. Die Läsionen sind unregelmäßig verteilt und weisen eine hyperkeratotische (verdickte) Textur auf. Die Farbe reicht von blassem Rosa bis zu leicht geröteten, entzündlichen Stellen. Dieses Erscheinungsbild ist typisch für Psoriasis (Schuppenflechte), eine chronische Autoimmunerkrankung, die sich häufig an den Streckseiten der Gelenke wie dem Ellenbogen manifestiert.					
4<br>Auf diesem klinischen Bild ist eine Hautläsion an einem vermutlich großen Gelenk (wie dem Ellbogen, der durch die Wölbung angedeutet wird) zu sehen. Die Läsionen zeigen rötliche bis erythematöse Plaques mit einer deutlichen, silbrig-weißen, schuppenden Oberfläche (Schuppen). Sie sind unregelmäßig geformt und in einem lokalisierten, gruppierten Muster verteilt. Die Haut um die Läsionen herum erscheint überwiegend hell und leicht trocken. Die Kombination aus scharf begrenzten, erythematösen Plaques und dicken, silbrigen Schuppen ist klassisch für Psoriasis (Schuppenflechte), insbesondere					

die Plaque-Psoriasis, die häufig die Streckseiten der Gliedmaßen wie die Ellbogen betrifft.					
5 Das klinische Bild zeigt eine Ansammlung von Plaques an der Außenseite eines Gelenks, höchstwahrscheinlich dem Ellbogen (typische Prädilektionsstelle). Die Läsionen erscheinen rötlich (erythematös) und sind mit silbrig-weißen, fest haftenden Schuppen bedeckt, was ihnen eine schuppige (squamöse) Textur verleiht. Die Verteilung ist lokalisiert und gruppiert. Diese charakteristischen dermatologischen Merkmale – erythematöse Plaques mit silbrigen Schuppen an einer Streckseite – sind stark suggestiv für Psoriasis (Schuppenflechte).					
6 Auf diesem klinischen Bild ist eine Hautläsion an einem Ellenbogen oder Knie (typische Lokalisation) zu sehen, die multiple, erythematöse (gerötete) Plaques aufweist. Diese Läsionen sind durch eine deutliche, silbrig-weiße Schuppung charakterisiert, die auf einer verdickten, leicht erhabenen Basis sitzt. Die Textur ist trocken und schuppig. Die Verteilung scheint fokal und leicht gruppiert zu sein. Die Gesamtmerkmale — gut definierte, erythematöse Plaques mit silbriger Schuppung an einer Streckseite eines Gelenks — sind stark suggestiv für Psoriasis (Schuppenflechte).					

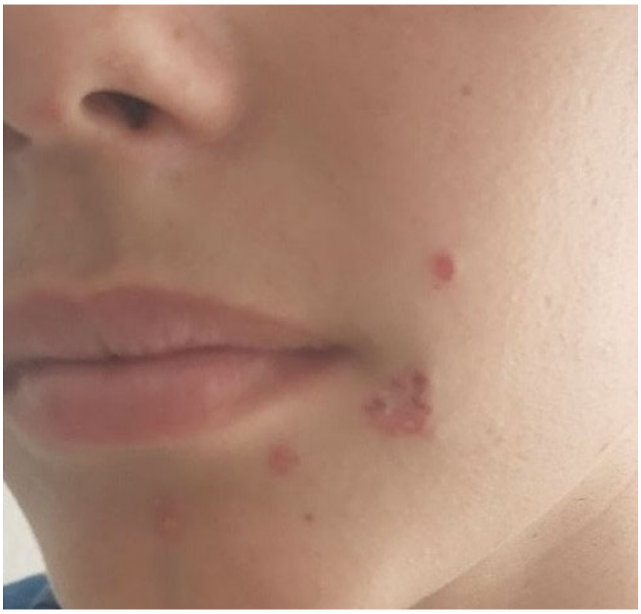

Description	Completeness	Lack of false information (accuracy)	Evidence-based content	Appropriateness	Relevance

1 In the clinical image, the skin around the corner of the mouth and on the chin shows a cluster of small, reddish, crusted lesions with an irregular border, which appears somewhat vesicular or erosive in texture. These are surrounded by a few scattered, smaller red papules. The overall appearance and perioral distribution are highly suggestive of a herpes simplex virus infection, commonly known as a cold sore or herpes labialis, though a differential diagnosis like impetigo or contact dermatitis would also need consideration by a medical professional.					
2 Based on the image, the patient presents with multiple erythematous papules and a clustered, erosive plaque on the perioral skin, specifically near the corner of the mouth and on the adjacent chin/cheek area. The lesions are red (erythematous), slightly raised papules, with the central cluster exhibiting a crusted or erosive texture and a somewhat irregular, polycyclic border. The distribution is localized and unilateral near the lower lip. The appearance of the clustered, erosive lesions is highly suggestive of Herpes Simplex Labialis (cold sore), although the scattered, smaller papules could represent adjacent irritation or a separate, less severe process like acne or folliculitis					
3 This clinical image shows the perioral area of a face, exhibiting several dermatological lesions. The most prominent feature is a cluster of erythematous (reddish) papulovesicles (small, raised, fluid-filled bumps) and erosions (breaks in the skin) located on the chin, lateral to the lower lip. This cluster has a somewhat crusted, irregular texture and a grouped distribution. There are also a few smaller, isolated, erythematous papules in the surrounding skin. The appearance of the clustered, erythematous, and vesicular/crusted lesions is highly suggestive of Herpes Simplex infection (cold sore).					
4 The image displays the peri-oral (around the mouth) area of a person's face. The most prominent dermatological feature is a cluster of vesicles or small erosions on an erythematous (red) base located just lateral and slightly inferior to the corner of the mouth on the lower cheek/chin area. This					

lesion appears somewhat crusting or scabbed in the center and is relatively localized. There are also a few scattered, smaller, erythematous papules (small, raised bumps) in the surrounding area. The distribution is unilateral (on one side) and localized to the peri-oral skin. Given the appearance of grouped vesicles on a red base in a common location, the possible differential diagnosis includes a Herpes Simplex Virus (HSV) infection, commonly known as a cold sore.					
5 Based on the image, the patient's skin around the mouth area shows multiple erythematous papules and a vesicular-appearing plaque. The lesions are primarily distributed on the lower chin and perioral region. The central cluster, close to the corner of the mouth, presents as a group of small, red bumps or vesicles on a red base, suggesting a vesiculopapular eruption. This specific presentation—clustered vesicles on an erythematous base near the mouth—is highly suggestive of Herpes Simplex Labialis (cold sore), a common viral infection. Other, smaller, scattered erythematous papules are also visible, potentially representing early or resolving lesions.					
6 The image shows the perioral (around the mouth) and chin area with several skin lesions. Most notably, there is a cluster of small vesicles or crusts on an erythematous (red) base located on the lower right side of the mouth. The overall texture in this area appears somewhat rough or crusted. Scattered around this central cluster and on the chin are several other small, erythematous papules or macules. Given the typical location and the vesicular/crusted grouped appearance, the central lesion is highly suggestive of Herpes Simplex Labialis (cold sore). The other individual lesions could be early stages of the same process, or possibly unrelated acne/folliculitis, but the prominent clustered lesion strongly points to a viral etiology.					
1 L'image présente une zone péribuccale avec des lésions cutanées visibles. On observe un groupement de vésicules ou de petites papules érythémateuses formant une plaque croûteuse et légèrement érosive juste					

à côté de la commissure des lèvres (zone péri-orale). La couleur est rouge vif (érythème) avec des zones jaunâtres ou brunâtres correspondant aux croûtes. La texture semble irrégulière et surélevée. La distribution est localisée et groupée. Ces caractéristiques, notamment l'aspect groupé des vésicules sur une base érythémateuse et la localisation péri-orale, sont très suggestives d'un Herpès Simplex labial (bouton de fièvre), bien qu'un diagnostic formel nécessite une évaluation médicale. D'autres petites papules érythémateuses isolées sont également visibles sur le menton et la joue adjacente.					
2<br>En se basant sur l'image clinique fournie, on observe une éruption cutanée localisée sur la zone péribuccale et le menton. Les lésions principales sont :<br>Des petites papules ou pustules érythémateuses (rouges), de taille variable, certaines isolées (notamment au-dessus et en dessous de l'amas principal) suggérant des lésions acnéiques ou inflammatoires.<br>Un amas de petites vésicules ou croûtes érythémateuses et potentiellement suintantes juste à côté de la commissure des lèvres (coin de la bouche). Cet amas présente une texture plus irrégulière et une distribution groupée caractéristique.<br>La distribution groupée et l'aspect vésiculeux/croûteux de l'amas principal sont fortement suggestifs d'un herpès labial (bouton de fièvre).					
3<br>D'après l'image, on observe sur la zone péri-buccale et mentonnière plusieurs lésions cutanées érythémateuses (rouges). L'une d'elles, située juste à côté de la commissure des lèvres, présente une texture vésiculeuse ou croûteuse et un aspect groupé en bouquet. Les autres sont des papules plus petites, isolées. Cette distribution groupée de vésicules sur une base érythémateuse, près de la bouche, est très suggestive d'un herpès labial (bouton de fièvre). Les lésions isolées pourraient être de l'acné ou d'autres éruptions, mais la lésion centrale groupée est la caractéristique dermatologique la plus frappante.					

4 L'image présente une zone cutanée péri-buccale (autour de la bouche) avec plusieurs lésions érythémateuses (rouges). On observe un groupement vésiculaire ou croûteux de petites lésions sur la joue inférieure droite, suggérant une texture irrégulière ou suintante dans cette zone centrale. Autour de ce foyer principal, ainsi que sur la lèvre inférieure et la zone mentonnière, on distingue des papules (petites bosses) ou de petites macules érythémateuses isolées. La distribution est localisée autour de la bouche. Ce tableau clinique, en particulier le regroupement de vésicules ou de croûtes dans un motif érythémateux, est fortement suggestif d'une infection herpétique (Herpès simplex, souvent appelé « bouton de fièvre »), bien que d'autres diagnostics (comme certaines formes d'acné inflammatoire ou de dermatite) ne puissent être exclus sans examen clinique.					
5<br>D'après l'image clinique, on observe plusieurs lésions cutanées sur la région péribuccale et la joue inférieure. La lésion la plus notable est un petit agrégat vésiculaire ou croûteux de forme irrégulière, rougeâtre et légèrement érosif, situé sur le côté droit du menton, près de la lèvre. Autour de cette lésion principale et sur la joue, on distingue également quelques papules érythémateuses (petits boutons rouges), certaines apparaissant comme des lésions plus petites et isolées ou des stades précoces/résolutifs. La couleur générale des lésions est rouge (érythémateuse). Cette distribution et cette morphologie, en particulier l'agrégat vésiculaire ou croûteux et les lésions satellites, sont fortement suggestives d'un Herpès labial (ou bouton de fièvre), une infection virale due au Herpes Simplex Virus (HSV).					
6 L'image présente une zone du visage autour de la bouche. On y observe plusieurs lésions cutanées érythémateuses (rouges) et surélevées. La zone la plus notable est un groupe de petites vésicules ou papules confluentes formant une plaque agrégée et croûteuse juste sous le coin de la lèvre inférieure. Autour de cette lésion principale, il y a quelques autres petites papules rouges					

isolées sur la joue et le menton. Les lésions sont localisées dans la région périorale.					
1 Στην παρούσα εικόνα, παρατηρείται η περιοχή γύρω από το στόμα (περιστοματική περιοχή) ενός ατόμου. Το πιο εμφανές δερματολογικό εύρημα είναι μια συρρέουσα ομάδα (cluster) μικρών, ερυθηματωδών (κόκκινων) φυσαλίδων ή μικρών διαβρώσεων/εφελκίδων (κρούστα) ακριβώς κάτω και πλάγια από τη γωνία του στόματος, η οποία υποδηλώνει μια ομάδα βλαβών. Η υφή φαίνεται να είναι επιφανειακή και φλεγμονώδης. Επιπλέον, υπάρχουν μερικές μεμονωμένες, ερυθρές κηλίδες ή μικροί βλαστοί (σπυράκια) διασκορπισμένες στην κοντινή περιοχή του προσώπου. Η κατανομή είναι εντοπισμένη στη μια πλευρά του προσώπου. Η εμφάνιση της συρρέουσας ομάδας βλαβών, ειδικά στην περιστοματική περιοχή, είναι ισχυρά συμβατή με τη διάγνωση του απλού έρπητα (κοινώς, επιχείλεις ή "πυρετού") στην ενεργό ή αρχόμενη φάση επούλωσης.					
2 Στην εικόνα παρατηρείται τμήμα του κάτω μέρους του προσώπου, γύρω από την περιοχή των χειλιών (περιστοματική περιοχή). Το πιο εμφανές δερματολογικό εύρημα είναι μία συρρέουσα, ερυθηματώδης (κόκκινη) πλάκα με ακανόνιστο περίγραμμα και φυσαλιδώδη ή εφελκιδοποιημένη επιφάνεια (μικρές φουσκάλες ή κρούστα), που βρίσκεται λίγο κάτω και δεξιά από την κάτω χείλη. Αυτό το εύρημα είναι τυπικό για ιογενείς λοιμώξεις, και συγκεκριμένα η πιθανότερη διάγνωση είναι ο επιχείλιος έρπης. Πιο μακριά από αυτήν την κύρια βλάβη, διακρίνονται και μερικά σποραδικά, μικρά, ερυθηματώδη κηλίδες ή βλατίδες (κόκκινα σπυράκια), τα οποία μπορεί να είναι είτε αρχικά στάδια της ίδιας ιογενούς λοίμωξης είτε τυπικές βλάβες ακμής ή φλεγμονής.					
3 Στην εικόνα παρατηρείται τμήμα του κάτω μέρους του προσώπου, κυρίως γύρω από την περιοχή των χειλιών (περιστοματική περιοχή) και του πηγουνιού. Το πιο εμφανές εύρημα είναι μια συστάδα από μικρές, ερυθηματώδεις βλάβες με φυσαλιδώδη ή εφελκιδοποιημένη υφή, οι οποίες έχουν μια σαφώς ακανόνιστη, ομαδοποιημένη					

κατανομή ακριβώς δίπλα στη γωνία του στόματος. Το χρώμα των βλαβών είναι κοκκινωπό/ροζ. Επιπλέον, διακρίνονται μερικές διάσπαρτες, μικρότερες, ερυθηματώδεις βλατίδες (πιθανόν ακνεϊκές βλάβες) στο γύρω δέρμα. Η ομαδοποιημένη και ερυθηματώδης βλάβη με φυσαλιδώδη/εφελκιδοποιημένη όψη στην περιστοματική περιοχή είναι ιδιαίτερα ενδεικτική για έρπητα χείλους (επιχείλιος έρπης ή κοινώς "πυρετός"), αν και μια οριστική διάγνωση απαιτεί κλινική εξέταση από δερματολόγο.					
4 Βάσει της εικόνας, παρατηρούνται ερυθρές βλατίδες και συρρέουσες φυσαλίδες (ή διαβρώσεις από ρήξη φυσαλίδων) με εφελκίδωση (κρούστα) στην περιοχή γύρω από το στόμα, συγκεκριμένα στη γωνία του στόματος και στο πηγούνι. Τα βλάβες είναι περιορισμένες και έχουν ομαδοποιημένη κατανομή, με μία χαρακτηριστική εστία στη γωνία του στόματος και μερικές μεμονωμένες βλατίδες λίγο πιο πάνω και κάτω. Το χρώμα των βλαβών είναι κυρίως ερυθρό και στην κεντρική, συρρέουσα βλάβη υπάρχει κιτρινωπή/καφεόχρους εφελκίδωση. Η εικόνα είναι ισχυρά ενδεικτική για έρπητα χείλους (επιχείλιο έρπητα), αν και άλλες καταστάσεις όπως το μολυσματικό κηρίο ή εκζεματίτιδα γωνίας στόματος θα μπορούσαν να τεθούν σε διαφορική διάγνωση.					
5 Η εικόνα δείχνει την περιοχή γύρω από το στόμα και το πηγούνι ενός ατόμου, όπου παρατηρούνται ερυθηματώδεις βλάβες στο δέρμα. Κοντά στην κάτω γωνία των χειλιών υπάρχει μία συρρέουσα βλάβη που αποτελείται από μικρότερες, ερυθηματώδεις, πιθανώς ορορροούσες ή εφελκιδοποιημένες (με κρούστα) μικρο-βλάβες, δίνοντας μία ακανόνιστη, φλεγμονώδη εμφάνιση. Άλλες μικρότερες, μεμονωμένες, ερυθρές βλατίδες ή κηλίδες διακρίνονται στην περιβάλλουσα περιοχή. Η κατανομή είναι περιορισμένη και εστιασμένη στην περιοχή του στόματος. Με βάση την εμφάνιση και την τυπική κατανομή, η συρρέουσα βλάβη είναι ιδιαίτερα ύποπτη για απλό έρπητα (κυρίως τύπου HSV-1), κοινώς γνωστό ως "επιχείλιος έρπης", ενώ οι άλλες βλάβες μπορεί να είναι είτε αρχόμενες					

ερπητικές βλάβες είτε φλεγμονώδεις βλάβες ακμής.					
6 Στην εικόνα παρατηρείται η περιοχή γύρω από το στόμα, εστιασμένη στη δεξιά γωνία του χείλους και στο παρακείμενο δέρμα.<br>Κύριο εύρημα είναι μία συρρέουσα βλάβη (μια ομάδα βλαβών που ενώνονται) στην περιοχή του πηγουνιού, η οποία φαίνεται ερυθηματώδης (κόκκινη) και επερυσμένη (με κρούστα). Η υφή φαίνεται να είναι φυσαλιδώδης ή εξελκωτική (πληγή) σε ορισμένα σημεία, υποδηλώνοντας πιθανώς μια φλεγμονώδη ή μολυσματική διεργασία.<br>Επιπλέον, υπάρχουν και μεμονωμένες, μικρότερες, ερυθηματώδεις βλατίδες (μικρά εξογκώματα) στην ίδια περιοχή. Η κατανομή είναι περιορισμένη και εντοπισμένη στο περικογχικό δέρμα.<br>Αυτά τα χαρακτηριστικά μπορεί να είναι συμβατά με καταστάσεις όπως:<br>Έρπητας Χειλιών : Ειδικά η συρρέουσα, φυσαλιδώδης, και ελκωτική βλάβη.<br>Μικρή Δερματίτιδα εξ' Επαφής: Λόγω ερεθισμού.<br>Πυοδερμία / Μόλυνση: Όπως μολυσματικό κηρίο.<br>Απλή ακμή ή θυλακίτιδα: Αν και η μορφολογία της κεντρικής βλάβης είναι λιγότερο τυπική.					
1 Auf dem klinischen Bild sind periorale Läsionen zu sehen, d.h. in der Nähe des Mundes. Die Hauptmerkmale sind:<br>Verteilung und Textur: Es handelt sich um eine gruppierte Anordnung von kleinen Läsionen, die hauptsächlich aus Bläschen (Vesikel) oder Erosionen bestehen, die zu einem leicht erhabenen, verkrusteten Bereich verschmelzen.<br>Farbe: Die betroffene Haut ist gerötet (erythematös). Die Krusten zeigen eine bräunliche bis gelbliche Färbung, was auf eine Heilungsphase oder eine sekundäre Infektion hindeuten kann.<br>Mögliche Diagnose: Das typische Bild von gruppierten Bläschen oder Krusten im Lippenbereich, oft auch als "Fieberbläschen" bekannt, weist stark auf einen Herpes labialis hin, verursacht durch das Herpes-simplex-					

Virus (HSV). Die herpetiforme (gruppierte) Anordnung ist ein charakteristisches Zeichen.					
2 Auf diesem klinischen Bild ist der Bereich um den Mundwinkel (perioral) und das Kinn einer Person zu sehen. Auffällig ist eine Gruppe von vesikulären oder krustösen Läsionen in der Nähe des rechten Mundwinkels, die eine rötliche Basis und eine leicht verkrustete oder erodierte Oberfläche aufweisen. Dies deutet auf eine clusterförmige Anordnung hin. Einzelne, diskretere rötliche Papeln oder Flecken sind ebenfalls auf der Wange und dem Kinn sichtbar. Die Läsionen sind nicht gleichmäßig verteilt; das größte und auffälligste Cluster befindet sich peri-oral. Aufgrund der klassischen gruppierten, vesikulären/krustösen Erscheinung an dieser Stelle besteht der Verdacht auf Herpes simplex (Lippenherpes), wobei die kleineren Läsionen auch akneähnliche Merkmale sein könnten. Eine genaue Diagnose erfordert jedoch eine professionelle Untersuchung.					
3 Das klinische Bild zeigt die untere Gesichtshälfte im Bereich des Mundwinkels und des Kinns. Es sind mehrere kleine, rötliche Läsionen auf der Haut zu sehen. Auffällig ist eine gruppierte Ansammlung von vesikulären oder krustigen Läsionen direkt neben dem Mundwinkel. Diese Stelle ist erythematös (gerötet) und weist eine leicht unebene, eventuell nässende oder verkrustete Textur auf, was auf eine aktive oder abheilende Läsion hindeutet. Einzelne, diskretere rötliche Flecken oder Papeln sind in der näheren Umgebung verteilt. Die Verteilung ist perioral (um den Mund herum) lokalisiert. Aufgrund der gruppierten Bläschenbildung oder Krusten auf erythematösem Grund, insbesondere in dieser perioralen Lokalisation, könnte es sich klinisch um einen Herpes labialis (Lippenherpes oder "Fieberbläschen") handeln, obwohl andere entzündliche oder infektiöse Hauterkrankungen nicht ausgeschlossen werden können.					
4 Das Bild zeigt im perioralen (um den Mund herum) und perilabiellen (um die Lippen herum) Bereich mehrere Läsionen auf der Haut. Das auffälligste Merkmal ist eine konfluierende (zusammenfließende) Gruppe					

von kleinen, flüssigkeitsgefüllten Bläschen (Vesikel), die auf einer geröteten (erythematösen) und leicht geschwollenen Basis liegt. Diese Bläschen haben eine gelbliche oder krustige Textur, was auf eine frühe Phase der Austrocknung/Krustenbildung oder eine Erosion der Bläschendächer hindeutet.<br>Merkmale und Mögliche Diagnose<br>Farbe: Die Läsionen sind überwiegend rötlich bis gelblich-bräunlich (Krusten), während die umgebende Haut normal erscheint.<br>Textur: Es liegt eine Bläschengruppe vor, die zu einer verkrusteten Plaque zusammenfließt. Die Haut ist dort erhaben und erscheint unregelmäßig/rau.<br>Verteilung: Die Läsionen sind gruppiert (herpetiform) und lokalisiert in der Nähe des Lippenrandes (Lippen-Wangen-Übergang).<br>Mögliche Diagnose: Die gruppierte Anordnung der Bläschen an dieser Stelle ist typisch für einen Lippenherpes (Herpes labialis), verursacht durch das Herpes-simplex-Virus. Andere, weniger wahrscheinliche, aber in Betracht zu ziehende Differentialdiagnosen könnten bestimmte Formen der Rosazea oder eine periorale Dermatitis sein, wobei das Bläschen-Krusten-Muster stark für Herpes spricht.					
5<br>Auf dem klinischen Bild sind im Bereich des rechten Mundwinkels und auf der angrenzenden Haut mehrere dermatologische Läsionen sichtbar. Auffällig ist ein gruppierter Ausschlag (Cluster) mit rötlicher Basis (erythematös) und kleinen, teils aufgebrochenen Bläschen oder Krusten, was auf eine aktive oder abheilende Bläschenbildung hindeutet. Die Läsionen sind überwiegend rötlich gefärbt, und ihre Verteilung ist auf den perioralen Bereich konzentriert, wobei sie sich sehr nah an der Lippe befinden. Einzelne kleinere, ebenfalls gerötete Läsionen sind weiter entfernt auf der Wange verteilt. Das typische Erscheinungsbild eines gruppierten Bläschenausschlags an oder um die Lippe, wie es hier zu sehen ist, deutet stark auf einen Herpes labialis (Lippenherpes) hin, verursacht durch das Herpes-simplex-Virus.					

6 Das Bild zeigt eine Region der perioralen Haut (um den Mund herum). Es sind mehrere rote, entzündliche Papeln und eine auffälligere Clusterbildung von Vesikeln oder kleinen Erosionen auf einem rötlichen, erythematösen Grund direkt neben der Lippe sichtbar. Die Läsionen erscheinen entzündet, mit einer leicht erhabenen Textur. Die Verteilung ist lokalisiert und gruppiert an einer Stelle, während andere einzelne Papeln in der näheren Umgebung verteilt sind. Die gruppierten Bläschen oder Krusten sind klinisch sehr suggestiv für Herpes simplex, möglicherweise ein rezidivierender Herpes labialis (Lippenherpes) . Die einzelnen rötlichen Papeln könnten ebenfalls frühe Herpesläsionen oder unspezifische entzündliche Papeln (z.B. Akne) darstellen, aber die gruppierte Läsion ist das bestimmende Merkmal.					

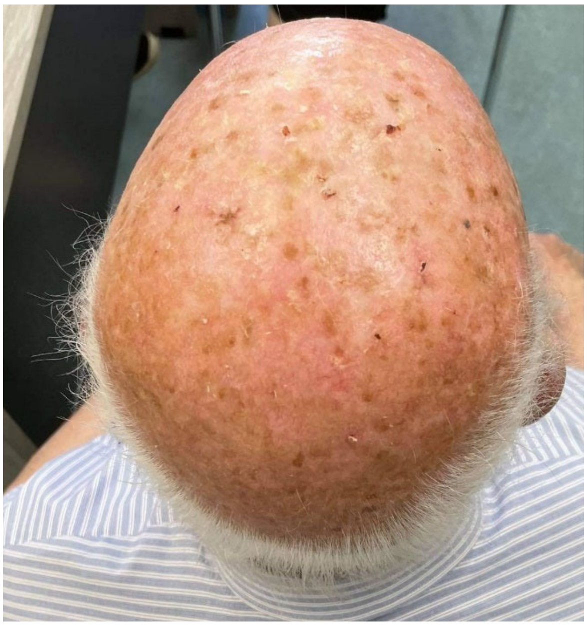

Description	Completeness	Lack of false information (accuracy)	Evidence-based content	Appropriateness	Relevance
1. The clinical image presents an overhead view of a bald scalp displaying significant chronic sun damage. The skin is erythematous (reddish) to tan/yellowish-brown, with an irregular texture. Scattered across the surface are multiple hyperpigmented macules (freckles/lentigines) and numerous small, crusty, scaling, and erythematous papules or macules. These lesions, which vary in size and color, are					

highly suggestive of Actinic Keratoses (AKs), which are premalignant lesions arising from prolonged UV exposure. The surrounding skin also shows signs of photoaging (elastosis and telangiectasias, although not all are clearly distinct in this image). This presentation is characteristic of a field cancerization process, where a large area of sun-exposed skin is at high risk for developing skin cancers, including squamous cell carcinoma.					
2.Based on the clinical image, the scalp appears erythematous (reddened) and diffusely sun-damaged with multiple scattered, ill-defined, hyperkeratotic (thickened, rough, or scaly) lesions. The skin texture is generally atrophic and poikilodermatous (showing mottled pigment changes, telangiectasias, and atrophy) over the vertex and crown, consistent with extensive chronic photodamage in an elderly, balding individual. The distribution of these rough lesions is widespread across the sun-exposed scalp. These findings are highly suggestive of Actinic Keratoses (AKs), which are common premalignant lesions arising from prolonged UV exposure.					
3.Based on the clinical image, the patient's scalp displays diffuse erythema (redness) and telangiectasias (small, widened blood vessels) indicative of chronic sun damage (photoaging). The texture is rough and scaly with multiple scattered, ill-defined, flesh-colored to light brown or yellow-brown papules and macules, consistent with actinic keratoses (AKs), which are precancerous lesions. The distribution is diffuse over the balding scalp, a sun-exposed site. The overall clinical picture strongly suggests Actinic Keratosis on a background of severe photodamage					
4. The image displays the bald scalp of an elderly individual, showing diffuse erythema (redness) and significant sun damage. The skin is characterized by a rough, scaly texture and multiple, scattered, irregular, yellowish-brown to reddish-brown macules and patches. There are also several small, crusted or slightly eroded areas and some hyperpigmentation (darker spots), suggestive of chronic sun exposure. The overall appearance is highly consistent with Actinic Keratosis (AK), a common precancerous condition, often developing on sun-exposed skin, superimposed on a background of chronic photodamage (dermatoheliosis).					
5.Based on the clinical image, the scalp of this individual appears largely alopecic (bald), showing diffuse erythema (redness) and a sun-damaged, thin-skinned texture. Multiple, scattered, small, hyperkeratotic, tan-to-brown papules and macules are visible, distributed broadly across the sun-exposed area. These features are highly suggestive of actinic keratoses (AKs), which are precancerous lesions resulting from chronic ultraviolet (UV) exposure, possibly in a context of field cancerization. There are also small areas of crusting and excoriation (scratch marks).					

6.The image displays the bald scalp of a patient, which exhibits multiple, scattered, irregular patches with a red to pink (erythematous) color and a rough, scaly, and crusty texture. These lesions are distributed over the sun-exposed areas of the scalp. The surrounding skin appears sun-damaged, suggesting a long history of chronic ultraviolet (UV) exposure. This presentation, marked by erythematous, rough, and scaly papules or plaques on a chronically sun-exposed site, is highly consistent with Actinic Keratosis (AK) (also known as solar keratosis), a common precancerous dermatological condition.					
1. L'image présente une vue de dessus du cuir chevelu d'un individu chauve (alopécie), affichant une peau érythémateuse (rougeâtre) et diffusément endommagée par le soleil (photovieillissement sévère). La texture de la peau apparaît épaissie, rugueuse et squameuse par endroits. On observe de multiples macules et papules hyperpigmentées (taches et petites bosses plus foncées), irrégulièrement distribuées sur tout le cuir chevelu, ainsi que des lésions croûteuses et érosives de petite taille, suggérant des zones de traumatisme ou de cicatrisation. Le diagnostic possible le plus pertinent, compte tenu de la distribution sur une zone exposée au soleil chez un sujet âgé, des lésions multiples, rugueuses et érythémateuses, est la présence d'une kératose actinique diffuse et avancée, une affection précancéreuse fréquente.					
2. L'image clinique montre le cuir chevelu d'un patient, principalement chauve (alopécie), avec une érythème (rougeur) diffuse sous-jacente. La couleur de la peau est globalement rosée à rougeâtre, et on note une pigmentation hétérogène avec des taches brunâtres (lentigos solaires ou autres macules pigmentées) et des zones de dyskératose/squames (petites croûtes ou plaques rugueuses et écailleuses) de couleur jaunâtre/brunâtre dispersées sur toute la surface. Ces lésions sont multiples, de petite taille, et confèrent à la peau une texture rugueuse et irrégulière. La distribution est diffuse sur le cuir chevelu exposé au soleil. Compte tenu de cet aspect clinique, notamment l'érythème et la présence de multiples lésions kératosiques, un diagnostic possible (différentiel) serait la présence de multiples kératoses actiniques (ou solaires) sur un fond de dommages solaires chroniques (élastose solaire, poïkilodermie de Civatte si elle s'étend aux zones photo-exposées).					
3. L'image présente une vue de dessus du cuir chevelu d'un individu, probablement âgé (cheveux blancs sur les côtés). Le cuir chevelu est alopécique (chauve) sur la partie supérieure, exposant la peau au soleil et aux intempéries. On observe une érythème (rougeur) diffuse de la peau,					

ainsi qu'une texture squameuse et rugueuse par endroits. De multiples lésions maculaires et papulaires de couleur brun-jaunâtre, de petite taille et bien définies, sont réparties sur toute la zone. Ces caractéristiques, en particulier l'érythème chronique, la desquamation, et les taches solaires multiples sur une zone photo-exposée et alopécique, sont fortement suggestives de la présence de kératoses actiniques diffuses (ou solaires). Un diagnostic différentiel inclurait une dermatite séborrhéique ou d'autres lésions pigmentées, mais la présentation est classique de l'héliodermie (dommages cutanés dus au soleil) chronique avec kératoses actiniques.					
4. L'image montre un cuir chevelu chauve présentant des signes marqués de dommages solaires chroniques (photovieillissement). La peau est érythémateuse (rougeâtre) avec une teinte légèrement jaunâtre/brunâtre et une texture visiblement rugueuse ou squameuse. On observe de multiples lésions discrètes, irrégulières, éparses, souvent hyperkératosiques (épaissies), allant du rose pâle au brun-rouge, sur une distribution très typique des zones exposées au soleil. Le diagnostic clinique le plus probable pour ces lésions multiples et diffuses chez un patient avec un phototype clair est la kératose actinique (KA) (ou kératose solaire), qui sont des lésions précancéreuses fréquentes résultant d'une exposition solaire excessive cumulée.					
5. L'image clinique montre un cuir chevelu chauve présentant un érythème diffus (rougeur) avec une desquamation fine et jaunâtre, suggérant une atteinte chronique par le soleil. On observe de multiples macules, papules et plaques brunâtres et kératosiques (rugueuses et squameuses) de taille variable, distribuées de manière éparse sur toute la calvitie. Ces lésions, notamment les plus rugueuses, sont très évocatrices de kératoses actiniques (ou solaires). La présence de ces lésions multiples, associées aux signes de dommages solaires chroniques, pose le diagnostic possible principal de kératoses actiniques multiples sur fond de dermite actinique chronique. Il est essentiel qu'un professionnel de la santé évalue ces lésions pour écarter toute transformation maligne.					
6. L'image clinique montre le cuir chevelu d'un individu, probablement âgé compte tenu de la calvitie et des caractéristiques cutanées. La peau est érythémateuse (rougeâtre) de manière diffuse et présente une texture rugueuse et écailleuse, en particulier au niveau des multiples lésions maculaires et papuleuses de couleur brun-jaunâtre à rouge-brun. Ces lésions sont réparties de façon assez irrégulière sur l'ensemble du cuir chevelu exposé. L'aspect général, caractérisé par une peau endommagée par le soleil (héliodermie) et ces multiples lésions kératosiques, est hautement suggestif d'une Kératose					

Actinique (KA), une condition précancéreuse fréquente dans les zones chroniquement exposées au soleil. Un diagnostic définitif et un plan de traitement nécessiteraient un examen clinique approfondi par un dermatologue.					
1. Η κλινική εικόνα παρουσιάζει την κορυφή του κρανίου ενός ατόμου, το οποίο εμφανίζεται φαλακρό στην περιοχή αυτή, με λίγες λευκές/γκρίζες τρίχες να διακρίνονται στην περιφέρεια (πίσω και πλάγια). Το δέρμα της κεφαλής είναι ερυθρό/ροζ (ερύθημα) με μια γενική κιτρινοκαφέ χροιά και παρουσιάζει ατροφία και διακριτή ξηρότητα/απολέπιση (συνεκτική με αίσθηση τραχύτητας, πιθανώς υπερκερατωσική). Παρατηρείται διάσπαρτη κατανομή πολλαπλών, μικρών, ακανόνιστων καφεοειδών/σκούρων κηλίδων (πιθανώς εφηλίδες ή φακές) και πολλαπλές υπερκερατωσικές βλάβες (τραχείες στην αφή) μερικές από τις οποίες εμφανίζουν μικρές εσχάρες ή επιφανειακές διαβρώσεις. Τα χαρακτηριστικά αυτά, σε έναν ασθενή μεγαλύτερης ηλικίας με εμφανή χρόνια φωτογήρανση και ιστορικό χρόνιας έκθεσης στον ήλιο, είναι ιδιαίτερα ενδεικτικά για πολλαπλές Ακτινικές Κερατώσεις επί εδάφους χρόνιας ηλιακής βλάβης, μια κοινή προκαρκινική κατάσταση.					
2. Παρατηρείται κεφαλή ηλικιωμένου ασθενούς με πλήρη αλωπεκία στην κορυφή. Το δέρμα στο κρανίο είναι ερυθηματώδες (κοκκινωπό) με διάσπαρτες υπερμελαγχρώσεις (καφέ κηλίδες) και πολλαπλές κερατινικές βλάβες (σκληρές, τραχιές περιοχές) που δίνουν μια ανομοιογενή και τραχιά υφή. Αυτή η κατανομή και τα χαρακτηριστικά είναι ιδιαίτερα ενδεικτικά της Ακτινικής Κεράτωσης (ΑΚ) επί εδάφους χρόνιας φωτογήρανσης (βλάβης από τον ήλιο), μια κοινή προκαρκινική δερματική πάθηση σε εκτεθειμένες στον ήλιο περιοχές, όπως το φαλακρό κρανίο.					
3. Η εικόνα απεικονίζει το δέρμα του τριχωτού της κεφαλής ενός ηλικιωμένου ατόμου, πιθανώς με ανδρογενετική αλωπεκία, καθώς το μεγαλύτερο μέρος του τριχωτού είναι φαλακρό. Το δέρμα εμφανίζει γενικευμένη ερυθρότητα (ερύθημα) και φωτογήρανση, χαρακτηριζόμενη από χρόνια έκθεση στον ήλιο. Παρατηρούνται πολλαπλές, διάσπαρτες, καφέ κηλίδες (πιθανώς εφηλίδες, φακές ή ηλιακές φακές) και υπερκερατωσικές, λεπιδώδεις, ερυθηματώδεις βλάβες (σκληρές στην αφή), που είναι συμβατές με Ακτινικές Κερατώσεις. Οι ακτινικές κερατώσεις είναι προκαρκινικές βλάβες που αναπτύσσονται σε περιοχές χρόνιας έκθεσης στον ήλιο. Επιπλέον, εμφανίζονται μερικές μικρές διαβρώσεις ή κρούστες. Η πιθανή διάγνωση που κυριαρχεί βάσει των ορατών χαρακτηριστικών είναι η Πολλαπλή					

Ακτινική Κεράτωση σε φόντο χρόνιας φωτοδερματίτιδας/φωτογήρανσης.					
4. Παρατηρείται μια εκτεταμένη βλάβη στο τριχωτό της κεφαλής ενός ηλικιωμένου ατόμου. Το δέρμα εμφανίζεται ερυθρό και κίτρινο-καφέ με διάσπαρτες κηλίδες και εφελκίδες (κοινώς, "κακάδια"). Η υφή είναι τραχιά και ξεφλουδισμένη σε ορισμένες περιοχές, υποδεικνύοντας υπερκερατωσικές αλλοιώσεις. Η κατανομή είναι διάχυτη στο άνω μέρος του κεφαλιού, το οποίο είναι εκτεθειμένο στον ήλιο. Αυτά τα χαρακτηριστικά είναι ιδιαίτερα ενδεικτικά της Ακτινικής Κεράτωσης, μιας προκαρκινικής βλάβης που προκαλείται από χρόνια έκθεση στην υπεριώδη ακτινοβολία, συχνά στο πλαίσιο της Χρόνιας Ηλιακής Βλάβης.					
5. Η εικόνα παρουσιάζει το τριχωτό της κεφαλής ενός ατόμου με ανδρογενετική αλωπεκία (αραιά μαλλιά ή φαλάκρα). Το δέρμα του τριχωτού της κεφαλής είναι ερυθρό/ροζ (ερύθημα) με συνοδό φωτογήρανση και δερματική βλάβη λόγω χρόνιας έκθεσης στον ήλιο. Παρατηρείται υπερκεράτωση και απολέπιση σε πολλαπλές, διάσπαρτες, ακανόνιστες βλάβες, με τη μορφή μικρών καφέ/κίτρινων/κοκκινωπών κηλίδων και πλακών, συχνά τραχιές στην αφή. Αυτά τα ευρήματα είναι άκρως ενδεικτικά για τη διάγνωση της Ακτινικής Κεράτωσης (ΑΚ), η οποία είναι προκαρκινική βλάβη. Η κατανομή των βλαβών είναι διάχυτη στην εκτεθειμένη στον ήλιο περιοχή του τριχωτού της κεφαλής.					
6. Αυτή η κλινική εικόνα από το άνω μέρος του τριχωτού της κεφαλής (πρόσωπο με αλωπεκία) ενός ηλικιωμένου ατόμου εμφανίζει εκτεταμένες δερματολογικές αλλοιώσεις. Το δέρμα φαίνεται ερυθρό (ερύθημα) με διάσπαρτες κηλίδες ανομοιόμορφης χρώσης (καστανοκίτρινες, πιθανώς φακίδες ή γεροντικές κηλίδες), πολλαπλές σκούρες κρούστες και υπερκερατωτικές πλάκες με τραχιά υφή, οι οποίες είναι ενδεικτικές ακτινικών κερατώσεων (προκαρκινικές αλλοιώσεις λόγω χρόνιας έκθεσης στον ήλιο). Η συνολική εμφάνιση, σε συνδυασμό με την έντονη φωτογήρανση και την απώλεια μαλλιών, είναι συμβατή με την εικόνα εκτεταμένης ηλιακής βλάβης και πεδίου καρκινογένεσης.					
1. Auf dem klinischen Bild ist die weitgehend unbehaarte Kopfhaut eines älteren Patienten zu sehen, was eine klassische sonnenexponierte Stelle darstellt. Die Haut weist eine diffuse Rötung (Erythem) auf und zeigt eine unregelmäßige Pigmentierung mit zahlreichen kleinen, bräunlichen Flecken (Lentigines solares). Zahlreiche multiple, umschriebene Läsionen sind erkennbar, die typischerweise eine rauhe, schuppige Textur aufweisen und					

teilweise krustig erscheinen. Diese Läsionen sind in ihrer Farbe variierend, oft rötlich, hautfarben oder bräunlich, und in verschiedenen Größen verteilt. Die Gesamtheit dieser Befunde – insbesondere die Verteilung an der Glatze, das diffuse Erythem und die rauen, schuppigen Flecken – deutet stark auf eine aktinische Keratose (auch solare Keratose genannt) hin. Diese Hautveränderungen gelten als Präkanzerose (Vorstufe des Plattenepithelkarzinoms) und sind auf chronische UV-Schädigung zurückzuführen.					
2. Das Bild zeigt eine glatzköpfige Kopfhaut, die eine diffuse Rötung (Erythem) und eine generell sonnengeschädigte (photo-gealterte) Textur aufweist. Die Haut ist ungleichmäßig gefärbt, mit Bereichen von gelblich-bräunlicher Verfärbung (Hyperpigmentierung) und zahlreichen, kleinen, verstreut liegenden rauhen, schuppigen, krustigen Läsionen. Diese Läsionen sind typischerweise rötlich-braun oder hautfarben und zeigen eine ungleichmäßige Verteilung über die gesamte Kopfhaut. Das klinische Erscheinungsbild, insbesondere bei einer chronisch sonnenexponierten Stelle wie der Kopfhaut eines älteren Mannes, ist hochverdächtig auf multiple aktinische Keratosen (AKs), die präkanzeröse Läsionen darstellen, die aus der kumulativen UV-Exposition resultieren. Die dünne, atrophische Haut im Hintergrund ist ebenfalls charakteristisch für schwere chronische Lichtschäden.					
3. Das klinische Bild zeigt die glatzköpfige Kopfhaut eines Patienten, die mehrere dermatologische Merkmale aufweist. Die Hintergrundfarbe der Haut erscheint gerötet (erythematös) mit einem insgesamt sonnengeschädigten (photogeschädigten) Aspekt. Die Textur der Kopfhaut ist unregelmäßig und rau. Es sind zahlreiche diskrete, gelblich-bräunliche Flecken (Lentigines oder Solar-Lentigines) sowie mehrere kleinere, rötlich-braune, schuppige, hyperkeratotische Plaques und Krusten unterschiedlicher Größe und Verteilung zu sehen. Diese schuppigen Läsionen sind typischerweise über die gesamte photogeschädigte Kopfhaut verteilt und sind stark verdächtig auf aktinische Keratosen (AK). Aktinische Keratosen sind Präkanzerosen (Vorstufen von Plattenepithelkarzinomen), die durch chronische UV-Exposition entstehen. Die Verteilung der Läsionen auf der sonnenexponierten Kopfhaut unterstützt diese mögliche Diagnose.					
4. Auf diesem klinischen Bild ist die Kopfhaut eines Patienten mit ausgeprägter chronischer Lichtschädigung (Photoaging) zu sehen. Die Haut erscheint erythematös (gerötet) und weist eine gelblich-bräunliche Verfärbung auf, was auf chronische Sonneneinstrahlung hindeutet. Es sind zahlreiche, unregelmäßig verteilte, diskrete, raue, schuppige Papeln oder Plaques sichtbar, die sich bei Berührung sandpapierartig anfühlen könnten. Diese Läsionen sind typisch für Aktinische Keratosen (AKs).					

Zusätzlich sind wahrscheinlich Lentigines solares (Altersflecken) und Bereiche mit Hautatrophie (dünnere Haut) sowie möglicherweise vereinzelte kleine Erosionen oder Krusten vorhanden. Die Verteilung der Läsionen ist auf die lichtexponierte Glatze beschränkt. Die primäre mögliche Diagnose ist die aktinische Keratose, eine präkanzeröse Läsion, die bei chronisch geschädigter Haut auftritt und sich zu einem Plattenepithelkarzinom entwickeln kann.					
5.<br>Auf dem klinischen Bild ist die Kopfhaut eines Patienten zu sehen, die kahl und stark der Sonne ausgesetzt ist. Die Haut zeigt eine ausgeprägte chronische Lichtschädigung (Photoalterung), erkennbar an einer diffusen Rötung (Erythem) und einer unregelmäßigen Pigmentierung (bräunliche Flecken/Makulae). Die Textur wirkt rau und verdickt, mit multiplen, unregelmäßig verteilten, krustigen oder schuppigen Läsionen, die hauptsächlich im oberen und zentralen Bereich konzentriert sind. Diese Merkmale, insbesondere die raue Textur und die diskreten Läsionen auf sonnengeschädigter Haut, sind charakteristisch für aktinische Keratosen (AK). Eine mögliche klinische Diagnose wäre eine ausgeprägte aktinische Keratose der Kopfhaut, ein präkanzeröser Zustand, der sich zu einem Plattenepithelkarzinom entwickeln kann.					
6. Das klinische Bild zeigt die Kopfhaut eines Patienten, die durch eine diffuse Rötung (Erythem) und eine gelblich-bräunliche Verfärbung (wahrscheinlich chronische Lichtschäden) gekennzeichnet ist. Die Textur der Kopfhaut erscheint rau und weist multiple, unregelmäßig verteilte, krustige oder schuppende Läsionen auf, die typischerweise als aktinische Keratosen (AKs) interpretiert werden können. Aktinische Keratosen sind präkanzeröse Läsionen, die durch jahrelange UV-Exposition entstehen. Zusätzlich sind einige kleinere, dunklere Flecken (Lentigines oder Pigmentflecken) und möglicherweise kleine Erosionen oder Krusten durch Kratzen sichtbar. Die Verteilung der Merkmale ist großflächig über die sonnenexponierte Glatze. Das gesamte Erscheinungsbild deutet auf eine ausgeprägte chronische Lichtschädigung (Photoaging) mit einer hohen Dichte an aktinischen Keratosen hin, was oft als Feldkanzerisierung bezeichnet wird und ein erhöhtes Risiko für die Entwicklung von Plattenepithelkarzinomen darstellt.					

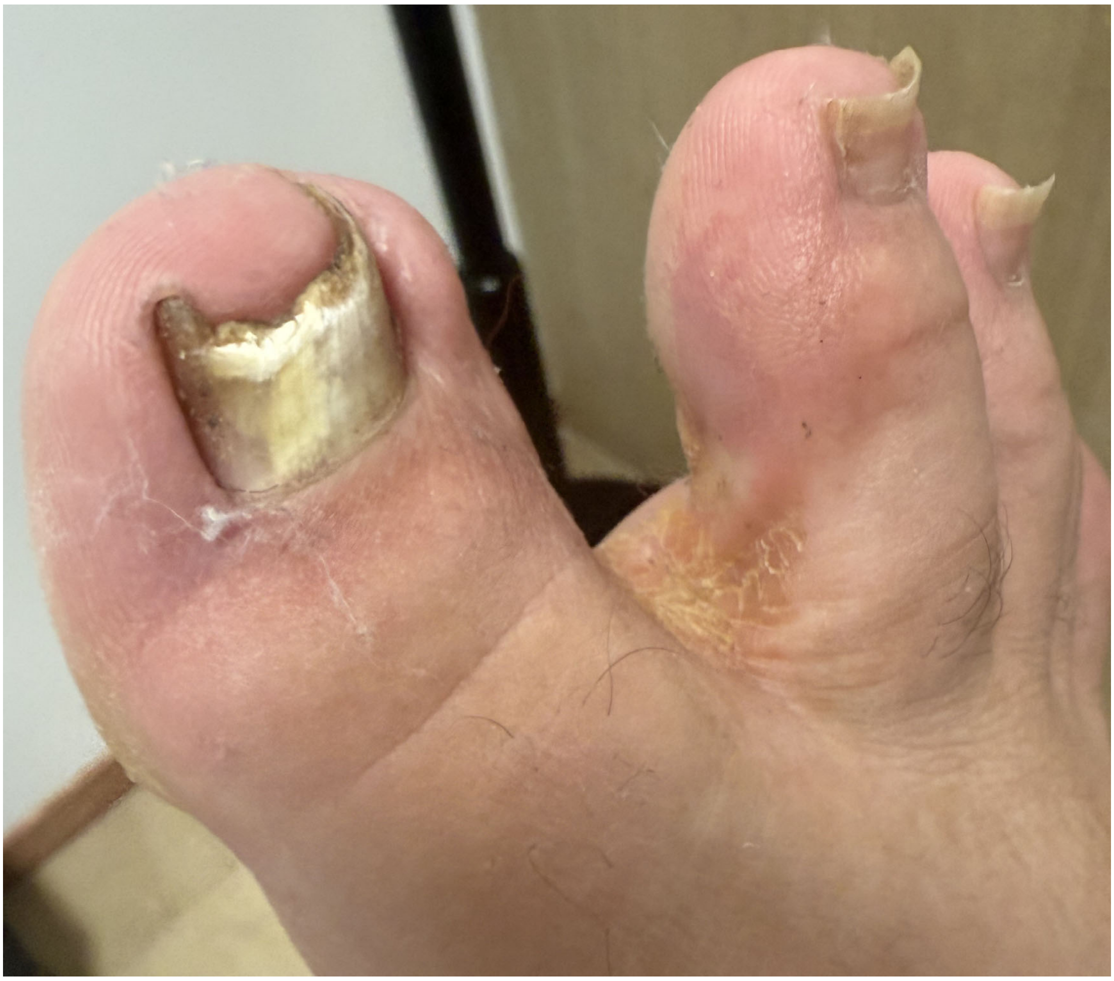

Description	Completeness	Lack of false information (accuracy)	Evidence-based content	Appropriateness	Relevance
1. The clinical image displays dermatological features consistent with Distal and Lateral Subungual Onychomycosis (DLSO), the most common form of fungal nail infection (tinea unguium). The entire nail plate appears thickened (hypertrophic) and severely discolored, exhibiting a yellowish-brown hue. The texture is rough and dystrophic, with prominent subungual hyperkeratosis (build-up of debris and thickened material) visible underneath the distal portion of the nail plate, causing the nail to lift (onycholysis). The infection has a predominantly distal and lateral distribution, starting at the free edge and sides of the nail and progressing proximally.					
2. The image shows the great toe and adjacent toes, primarily exhibiting features suggestive of onychomycosis (fungal nail infection) and tinea pedis (athlete's foot). The great toenail is significantly affected, displaying subungual hyperkeratosis (thickening under the nail) and onychodystrophy, with a marked yellow-to-brown discoloration and a rough, crumbly texture at the distal edge,					

consistent with distal and lateral subungual onychomycosis (DLSO). The skin in the web space between the great toe and the second toe shows erythema (redness), scaling, and possible maceration (softening due to moisture), which are classic signs of a dermatophyte infection like tinea pedis, often with an interdigital distribution. The remaining toenails also appear thickened and discolored.					
3. This clinical image shows the great toe (hallux) and adjacent toes, exhibiting several prominent dermatological features. The nail plate of the great toe appears significantly dystrophic, presenting with a yellowish-brown discoloration and marked thickening (hyperkeratosis), particularly distally, suggesting onychomycosis (fungal infection of the nail). There is also evidence of subungual debris and possible onycholysis (separation of the nail from the nail bed). The surrounding skin (perionychium) is somewhat erythematous (reddened) and slightly swollen. Between the great toe and the second toe, there is an area of erythema and scaling with some maceration (whitening and softening from moisture), consistent with an interdigital tinea pedis (athlete's foot), likely caused by the same fungal pathogen. The overall appearance is highly suggestive of tinea unguium (onychomycosis) complicated by tinea pedis.					
4. The image displays the great toe and adjacent toes, showing several dermatological features. The nail plate of the great toe is markedly dystrophic, characterized by hyperkeratosis, discoloration (a yellowish-brown/gold hue), and subungual debris. The nail appears thickened and partially lifted from the nail bed (onycholysis). This presentation is highly suggestive of onychomycosis (a fungal infection of the nail). Additionally, the skin in the interdigital web space between the first and second toes shows erythema (redness), scaling, and possibly maceration (whitening and softening from moisture), which is typical for tinea pedis (Athlete's foot), often caused by the same organisms that cause onychomycosis. The adjacent toe nails also show some distal onycholysis and minor changes.					
5. Based on the clinical image, the primary observation is an onychomycosis (fungal nail infection) affecting the great toenail. The nail appears significantly thickened (hypertrophic), distorted, and shows marked yellowish-brown discoloration, suggesting a possible distal and lateral subungual onychomycosis (DLSO) pattern. The skin of the great toe and the adjacent lesser toe displays features consistent with tinea pedis (athlete's foot), specifically a scaling and macerated texture with erythema (redness) in the interdigital space and on the skin below the great toenail, indicating a concurrent dermatophyte infection of the surrounding skin.					
6. The image primarily shows the hallux (big toe) and second toe of a foot, exhibiting several dermatological findings. The					

nail plate of the hallux appears significantly dystrophic, presenting with a yellowish-brown discoloration and marked thickening (hyperkeratosis), consistent with onychomycosis (fungal nail infection). There is also evidence of subungual debris and an irregular, lifted free edge. The skin in the interdigital space between the hallux and the second toe shows erythema (redness), scaling, and some signs of maceration and fissuring, suggesting an associated interdigital tinea pedis ("athlete's foot") or possibly an irritant/contact dermatitis, common secondary findings to the primary nail condition. The texture of the surrounding skin appears generally dry. The distribution is localized to the toes and interdigital web space.					
1. J'observe sur cette image clinique une vue rapprochée de deux orteils. L'hallux (gros orteil) présente une ongle visiblement épaissi, décoloré avec une teinte jaune-brunâtre et une texture irrégulière/cassante, ce qui est fortement suggestif d'une onychomycose (infection fongique de l'ongle). L'ongle semble également partiellement décollé du lit unguéal (onycholysis). La peau du gros orteil et de l'orteil adjacent présente une zone de desquamation (peau qui pèle) et de rougeur/inflammation localisée, notamment entre les orteils et sur le côté de l'orteil adjacent. Cette atteinte cutanée, caractérisée par une peau squameuse et fissurée, est compatible avec une tinea pedis (pied d'athlète), souvent causée par le même type de champignon que l'onychomycose. La distribution est localisée aux orteils, affectant l'ongle et la peau interdigitale et adjacente.					
2. L'image présente une vue rapprochée des orteils. L'aspect le plus frappant est celui du gros orteil (hallux), dont l'ongle montre une décoloration jaunâtre à brunâtre (chromonychie) avec un épaississement et une déformation marquée (hyperkératose sous-unguéale et onychodystrophie), des signes très évocateurs d'une onychomycose (infection fongique de l'ongle). Il y a également une inflammation et une légère rougeur autour des bords de l'ongle. De plus, l'espace entre le gros orteil et le deuxième orteil présente une desquamation (peau qui pèle) avec des fissures et une rougeur (érythème) modérée, ce qui suggère fortement un intertrigo ou une tinea pedis (pied d'athlète) interdigitale, souvent causée par le même type de champignon que l'onychomycose.					
3. L'image présente une vue rapprochée de deux orteils. L'hallux (gros orteil) montre des signes marqués de dystrophie unguéale, avec une plaque unguéale épaissie (hyperkératose sous-unguéale) et une décoloration significative allant du jaune-blanc au brun-noir, notamment au centre et à la partie distale de l'ongle. La texture de l'ongle est rugueuse et friable, et il semble y avoir une					

séparation partielle du lit unguéal (onycholyse). Ces signes sont fortement suggestifs d'une onychomycose (infection fongique de l'ongle). L'espace interdigital entre le gros orteil et le deuxième orteil, ainsi que la base du deuxième orteil, présente une zone d'érythème (rougeur) avec une desquamation et une macération, caractérisée par une texture squameuse et fissurée de couleur jaunâtre, ce qui est compatible avec un intertrigo ou un tinea pedis (pied d'athlète) de type interdigital. La peau environnante est sèche.					
4. C'est une image clinique des orteils, principalement le gros orteil. On observe au niveau du gros orteil une dystrophie unguéale (changement d'apparence de l'ongle) significative. L'ongle présente une décoloration marquée, tirant sur le jaune-brun et semble épaissi (hyperkératose sous-unguéale), avec une surface rugueuse et des bords irréguliers. Il y a également un décollement partiel de la tablette unguéale (onycholyse) au bord distal. La peau adjacente (péri-unguéale) du gros orteil semble érythémateuse (rougeâtre) et peut-être légèrement squameuse par endroits. L'orteil voisin présente également des signes d'infection ou d'irritation de la peau, avec des squames jaunâtres et une desquamation visible à la base et entre les orteils. La distribution de ces anomalies est localisée aux orteils, affectant principalement le gros orteil et la peau adjacente.Le diagnostic dermatologique le plus probable pour cet ensemble de caractéristiques est l'Onychomycose (infection fongique de l'ongle), souvent associée à une Tinea Pedis (pied d'athlète) pour l'atteinte cutanée interdigitale et plantaire. Cependant, d'autres affections comme le Psoriasis unguéal ou les conséquences d'un traumatisme chronique (dystrophie post-traumatique) peuvent également provoquer un épaississement et une décoloration similaires, nécessitant une confirmation médicale par prélèvement mycologique.					
5. Cette image clinique montre les orteils d'un pied, avec des anomalies dermatologiques notables. L'ongle du gros orteil (hallux) présente une décoloration jaune-brunâtre marquée, une épaississement (hypertrophie) et une texture irrégulière/friable, caractéristiques qui suggèrent fortement une onychomycose sévère (infection fongique de l'ongle). De plus, la peau entre le gros orteil et l'orteil adjacent présente une rougeur (érythème), une desquamation (peau qui pèle) et des fissures ou macérations légères dans le pli interdigital, ce qui est très évocateur d'un pied d'athlète (tinea pedis) interdigital. Ces deux conditions, l'onychomycose et le tinea pedis, sont couramment associées et sont causées par des infections fongiques.					
6. Cette image présente le gros orteil et l'orteil adjacent, révélant plusieurs caractéristiques dermatologiques notables. L'ongle du gros orteil est visiblement affecté,					

montrant une décoloration jaune-brunâtre (chromonychie) avec une hyperkératose sous-unguéale prononcée, soulevant la plaque unguéale (onycholyse). L'ongle lui-même apparaît épaissi (onychogryphose) et déformé avec des signes d'épaississement distal. Les bords de l'ongle sont effrités ou irréguliers. Ces signes sont fortement suggestifs d'une onychomycose, une infection fongique de l'ongle. De plus, la peau entre le gros orteil et le deuxième orteil présente une desquamation (peeling) et une rougeur (érythème), potentiellement liées à une infection fongique cutanée associée (comme le pied d'athlète ou tinea pedis).					
1. Βάσει της εικόνας, παρατηρείται μια κλινική εικόνα που επηρεάζει κυρίως τον μεγάλο δάκτυλο του ποδιού και τη γύρω περιοχή. Το νύχι του μεγάλου δακτύλου εμφανίζει σοβαρή δυσχρωμία (κιτρινωπό-καφετί) και υπερκεράτωση (πάχυνση) με πιθανή ονυχόλυση (αποκόλληση), χαρακτηριστικά που συνάδουν με ονυχομυκητίαση.Η υφή του νυχιού είναι τραχιά και παραμορφωμένη. Στο διάστημα μεταξύ του μεγάλου δακτύλου και του δευτέρου, καθώς και στη βάση του δευτέρου δακτύλου, υπάρχει ερύθημα (κοκκίνισμα), απολέπιση και ίσως μικρές φυσαλίδες ή σκασίματα, ευρήματα που υποδηλώνουν ποδαγρικό μύκητα με μεσοδακτύλια κατανομή. Η πιθανότερη συνδυαστική διάγνωση είναι ονυχομυκητίαση και δερματοφυτία (μύκητας) των ποδιών.					
2. Βλέπω μια εικόνα που απεικονίζει τα δάχτυλα του ποδιού, με εμφανή δερματολογικά χαρακτηριστικά. Ο ονυχας του μεγάλου δακτύλου παρουσιάζει ονυχομυκητίαση (είδος μυκητιασικής λοίμωξης), η οποία χαρακτηρίζεται από δυσχρωμία (κιτρινωπό/καφέ χρώμα) και πάχυνση του νυχιού (ολοκληρωτική ονυχοδυστροφία). Παρατηρείται επίσης υπερκεράτωση κάτω από το νύχι (υπονύχια) και παραμόρφωση της φυσιολογικής του δομής. Επιπλέον, στο διάστημα μεταξύ του μεγάλου δακτύλου και του δεύτερου δακτύλου, καθώς και στη βάση του δεύτερου, υπάρχει ερύθημα (κοκκίνισμα) και εμφανής απολέπιση (ξεφλούδισμα) του δέρματος, πιθανώς λόγω δερματοφυτίας (ringworm) ή μύκητα των ποδιών (tinea pedis) στην μεσοδακτύλια μορφή της. Η εικόνα υποδηλώνει μια χρόνια λοίμωξη που επηρεάζει τόσο τα νύχια όσο και το γύρω δέρμα.					
3. Βλέπω μια εικόνα που απεικονίζει τα δάχτυλα ενός ποδιού, όπου παρατηρούνται εμφανή δερματολογικά ευρήματα. Το νύχι του μεγάλου δακτύλου παρουσιάζει παχύνσεις, δυσχρωμία (κιτρινωπό-καφέ χρώμα) και ανώμαλη/τραχιά επιφάνεια, χαρακτηριστικά που συνάδουν με ονυχομυκητίαση (tinea unguium) ή ενδεχομένως ονυχογρύπωση. Στο δέρμα μεταξύ του μεγάλου και του					

διπλανού δακτύλου, υπάρχει ερύθημα (κοκκίνισμα), απολέπιση (ξεφλούδισμα) με σαφή όρια, και μια υγρή/μαλακή (διαβρωτική) εμφάνιση, στοιχεία που είναι τυπικά για δερματοφυτία των ποδιών (tinea pedis) τύπου μεσοδακτύλιου ή ποδιού του αθλητή. Η κατανομή των αλλοιώσεων είναι εστιασμένη στο νύχι και τον μεσοδακτύλιο χώρο.					
4. Η κλινική εικόνα απεικονίζει τα δάκτυλα του ποδιού. Το μεγάλο δάκτυλο εμφανίζει έντονη πάχυνση, κιτρινό-καφετί αποχρωματισμό και παραμόρφωση της ονυχαίας πλάκας (υπερκεράτωση και δυσμορφία), χαρακτηριστικά συμβατά με ονυχομυκητίαση (onychomycosis). Η περιονύχια περιοχή γύρω από το μεγάλο δάκτυλο φαίνεται ελαφρώς ερυθρή. Επιπλέον, στον ιστό ανάμεσα στο μεγάλο δάκτυλο και στο διπλανό δάκτυλο, παρατηρείται απολέπιση (ξεφλούδισμα) του δέρματος και πιθανή ερυθρότητα και διαβροχή, κατανομή που είναι τυπική για δερματοφυτία των ποδιών (tinea pedis) ή αλλιώς "πόδι του αθλητή", ειδικά στην περιοχή μεταξύ των δακτύλων (interdigital tinea pedis). Τα νύχια των άλλων δακτύλων επίσης φαίνονται υπερτροφικά και δυσμορφικά, αλλά λιγότερο έντονα από αυτό του μεγάλου δακτύλου. Συνολικά, η εικόνα είναι συμβατή με την παρουσία μυκητιασικής λοίμωξης (δερματοφυτία) που επηρεάζει τόσο το δέρμα όσο και τα νύχια.					
5. Παρατηρείται ορατή παραμόρφωση και αποχρωματισμός του νυχιού του μεγάλου δακτύλου του ποδιού, το οποίο εμφανίζει έντονο κιτρινωπό-καφετί χρώμα και παχύρρευστη, ανώμαλη υφή με σημάδια υπερκεράτωσης κάτω από την πλάκα του νυχιού (υπονύχια υπερκεράτωση). Η άκρη του νυχιού φαίνεται να είναι ξεφλουδισμένη ή μερικώς κατεστραμμένη. Στην περιοχή μεταξύ του πρώτου και του δεύτερου δακτύλου, υπάρχει μια ερυθηματώδης, φολιδωτή και ξηρή περιοχή του δέρματος με λέπια και ραγάδες (πιθανώς διαβρώσεις), υποδηλώνοντας φλεγμονή και απολέπιση. Τα ευρήματα στο νύχι είναι εξαιρετικά ενδεικτικά της Ονυχομυκητίασης (λοίμωξη του νυχιού από μύκητα), ενώ η μεσοδακτύλια βλάβη είναι συμβατή με Πόδι του Αθλητή, συχνά μία διαβρωτική ή φολιδωτή μορφή, η οποία συνήθως προκαλείται επίσης από μύκητες.					
6. Παρατηρείται μια κλινική εικόνα που αφορά τα δάχτυλα του ποδιού. Το μεγάλο δάχτυλο παρουσιάζει ορατή πάχυνση και δυσχρωμία του όνυχα, ο οποίος έχει ένα κιτρινωπό-καφετί χρώμα και ανώμαλη, κατεστραμμένη υφή. Τα χαρακτηριστικά αυτά είναι ισχυρά ενδεικτικά ονυχομυκητίασης (μυκητιασική λοίμωξη των νυχιών). Στο δεύτερο δάχτυλο και στο διάστημα μεταξύ του πρώτου και του δευτέρου δακτύλου διακρίνεται ένα ερυθρό, φολιδωτό (με λεπτές νιφάδες) και σκασμένο δέρμα, με πιθανή κατανομή που παραπέμπει σε δερματοφυτία (tinea pedis) ή μύκητα του ποδιού. Συνολικά, η εικόνα υποδηλώνει την παρουσία μυκητιασικών λοιμώξεων στο πόδι, οι οποίες					

επηρεάζουν τόσο τον όνυχα όσο και το μεσοδακτύλιο δέρμα.					
1. Auf dem klinischen Bild ist ein Fuß mit deutlichen dermatologischen Auffälligkeiten zu sehen, insbesondere am großen Zeh und im Zehenzwischenraum. Der Nagel des großen Zehs zeigt eine ausgeprägte Verfärbung, die hauptsächlich gelb-bräunlich ist, sowie eine verdickte und brüchige Textur mit Hyperkeratose unter der Nagelplatte. Dies ist ein starker Hinweis auf eine Onychomykose (Nagelpilz). Die umliegende Haut des großen Zehs erscheint leicht gerötet und möglicherweise geschwollen. Im Zehenzwischenraum zwischen dem großen Zeh und dem zweiten Zeh ist eine schuppige, gerötete und leicht nässende oder mazerierte Läsion zu erkennen, die typischerweise auf Tinea pedis (Fußpilz), oft in der interdigitalen Form, hindeutet. Die Verteilung der Merkmale ist auf die Zehennägel und die interdigitalen Bereiche konzentriert, was die gemeinsame Präsentation dieser Pilzinfektionen unterstreicht.					
2. Das klinische Bild zeigt eine deutliche dystrophische Veränderung des Nagels der Großzehe. Der Nagel weist eine unregelmäßige, verdickte Textur und eine auffällige gelblich-bräunliche Verfärbung auf, insbesondere distal und subungual (unter dem Nagel), was auf eine Hyperkeratose und eine mögliche Onycholyse (Ablösung des Nagels vom Nagelbett) hindeutet. Diese Merkmale sind stark suggestiv für eine Onychomykose (Nagelpilz), wahrscheinlich vom distolateralen subungualen Typ (DLSO). Zusätzlich sind im angrenzenden Interdigitalraum zwischen Großzehe und zweiter Zehe Hautveränderungen zu erkennen: Die Haut erscheint gerötet, schuppig und zeigt eine Mazeration (aufgeweichtes, weißliches Aussehen) im Bereich der Zehenfalte. Diese interdigitale Verteilung der Läsionen mit Schuppung und Rötung passt klinisch zur Tinea pedis (Fußpilz), der oft der Onychomykose vorausgeht oder gleichzeitig auftritt. Die Nägel der kleineren Zehen scheinen ebenfalls leicht verdickt und verfärbt zu sein, was auf eine Ausbreitung der Nagelbeteiligung hindeutet.					
3. Das klinische Bild zeigt die Großzehe und die zweite Zehe eines Fußes mit deutlichen dermatologischen Auffälligkeiten. Das Großzehennagel ist erheblich verändert und weist eine ausgeprägte gelblich-bräunliche Verfärbung auf, was als Chromonychie bezeichnet wird. Die Nagelplatte erscheint verdickt (Onychauxis), brüchig und in der Textur unregelmäßig. Die distalen und lateralen Ränder des Nagels sind erhaben oder deformiert. Diese Merkmale, insbesondere die Verfärbung und Verdickung, sind stark suggestiv für eine Onychomykose (Nagelpilzinfektion). Im					

Bereich zwischen der Großzehe und der zweiten Zehe (interdigital) ist eine rötliche, schuppende und leicht nässende Hautveränderung sichtbar, die auf eine interdigitale Tinea pedis (Fußpilz) oder Intertrigo hinweisen könnte. Die Haut um den Nagel der Großzehe wirkt leicht gerötet und geschwollen, möglicherweise aufgrund einer begleitenden Paronychie oder Entzündung. Die Verteilung der Läsionen ist primär auf den Großzehennagel und den interdigitalen Bereich zwischen Großzehe und zweiter Zehe beschränkt.					
4. Das Bild zeigt eine Nahaufnahme der Zehen eines Fußes, wobei die große Zehe und die zweite Zehe im Fokus stehen. Das auffälligste Merkmal ist die Veränderung des Nagels der großen Zehe (Onychodystrophie): Er ist gelb-bräunlich verfärbt, verdickt (Hyperkeratose) und weist eine veränderte, brüchige Textur mit Ablösungen (Onycholyse) auf. Die sichtbaren Merkmale sind stark suggestiv für eine Onychomykose (Nagelpilzinfektion). Die umgebende Haut der großen Zehe erscheint leicht gerötet (Erythem) und möglicherweise geschwollen. Zwischen der großen und der zweiten Zehe ist eine schuppige, leicht gerötete und aufgeplatzte (mazerierte) Hautläsion erkennbar, die typisch für eine Tinea pedis (Fußpilz), insbesondere die interdigitale Form, ist. Die gesamte Erscheinung deutet auf eine Pilzinfektion des Nagels und der interdigitalen Hauträume hin.					
5.<br>Dieser klinische Ausschnitt zeigt die Zehen, wobei sich die dermatologischen Merkmale auf den großen Zeh und den angrenzenden Zehenzwischenraum konzentrieren. Am Nagel des Großzehs ist eine deutliche dystrophische Veränderung zu erkennen, charakterisiert durch eine gelblich-braune Verfärbung, deutliche Verdickung (Hyperkeratose) und Brüchigkeit/Absplitterung der Nagelplatte, was mit einer Onychomykose (Nagelpilz), insbesondere der distalen subungualen Form (DSO), vereinbar ist. Die Verteilung der Verfärbung beginnt distal und breitet sich subungual aus. Die angrenzende Haut im Zehenzwischenraum (interdigital) und an der Basis des zweiten Zehs weist Läsionen auf, die durch Erythem (Rötung), Schuppung, und Bereiche von Mazeration oder oberflächlicher Erosion (hellere, feucht aussehende Bereiche mit Ablösung der obersten Hautschichten) gekennzeichnet sind. Diese interdigitale Hautveränderung ist hochsuggestiv für eine Tinea pedis (Fußpilz) des interdigitalen Typs, welche häufig mit einer Onychomykose vergesellschaftet ist.					
6. Das klinische Bild zeigt eine Nahaufnahme der Zehen eines Fußes, wobei mehrere dermatologische Merkmale auffallen. Der Nagel der Großzehe (Hallux) weist eine deutliche Onychomykose (Nagelpilz) auf, erkennbar an der gelblich-braunen bis gelblich-grünen Verfärbung und einer					

verdickten, brüchigen Textur (Hyperkeratose und Onychodystrophie), insbesondere im distalen und lateralen Bereich. Die Verfärbung und Verdickung sind hauptsächlich unter dem Nagel und an der Nagelplatte selbst sichtbar. Im Zehenzwischenraum zwischen der Großzehe und der zweiten Zehe ist eine schuppende, erythematöse (gerötete) Hautstelle mit leichter Mazeration (Aufweichung durch Feuchtigkeit) zu sehen, die auf eine Tinea pedis (Fußpilz), insbesondere die interdigitale Form, hindeutet. Die Nägel der kleineren Zehen erscheinen ebenfalls leicht verdickt und verfärbt, was eine Beteiligung an der Onychomykose nahelegt. Die gesamte Haut der Zehen erscheint leicht trocken.					

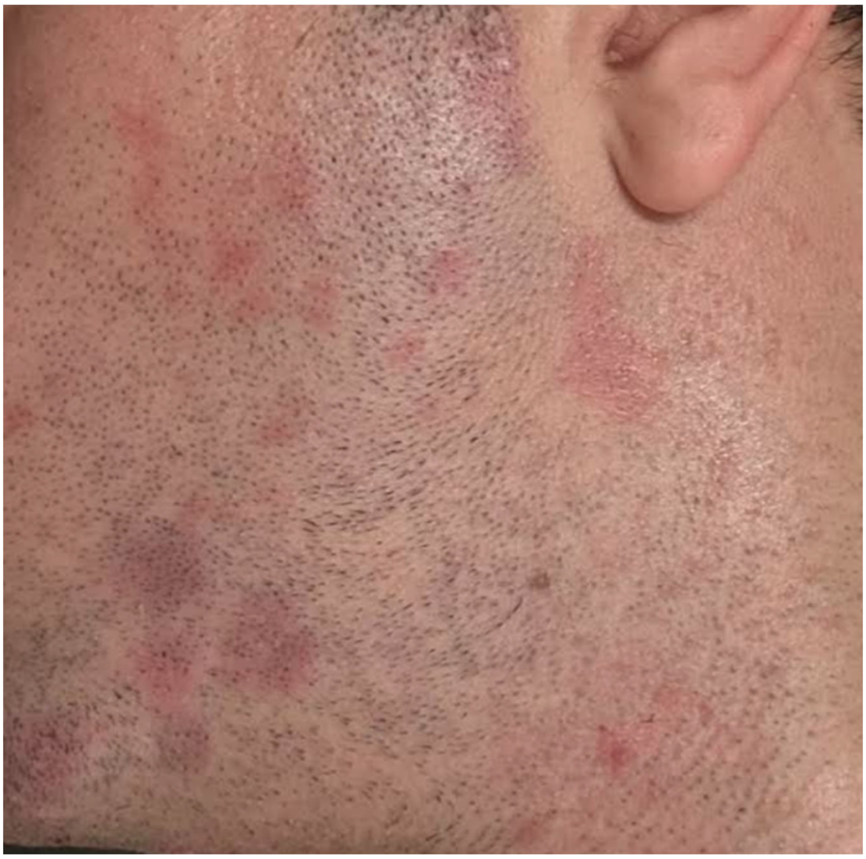

Description	Completeness	Lack of false information (accuracy)	Evidence-based content	Appropriateness	Relevance
1.The image shows the lower cheek and jawline of an adult male, exhibiting multiple erythematous papules and pustules scattered across the beard area, indicative of inflammation. The lesions are generally small to medium in size, red to reddish-purple (erythematous), and appear somewhat					

irregular in shape, especially where they coalesce. The texture is characterized by bumps and inflamed follicles overlying the closely cropped hair shafts (stubble). The distribution is primarily over the shaving area. The overall presentation is highly suggestive of Pseudofolliculitis Barbae (razor bumps), which often occurs in individuals with curly hair after shaving, or potentially a form of folliculitis.					
2. Based on the image, the skin on the side of the face and jawline, which has short, coarse hair (stubble), shows multiple erythematous (red to purplish-red) papules and pustules. The texture appears somewhat bumpy and inflamed. These lesions are clustered and scattered across the hair-bearing skin. This clinical presentation is highly suggestive of pseudofolliculitis barbae (razor bumps) or folliculitis, which are common inflammatory conditions of the hair follicles in this area, often exacerbated by shaving.					
3.<br>Based on the image, the patient's skin, primarily in the beard area of the lower face/jaw, exhibits multiple erythematous papules and patches and areas of follicular inflammation. The lesions are characterized by redness (erythema) and slight swelling, some appearing to be centered around hair follicles. The texture is somewhat irregular due to the papules and the surrounding stubble. The distribution is localized to the shaved area. This presentation is highly suggestive of Pseudofolliculitis Barbae (razor bumps), an inflammatory reaction common in people with curly hair who shave, where hair curves back into the skin causing inflammation. However, other diagnoses like folliculitis (bacterial or fungal) could also present similarly.					
4. The image displays a close-up of a person's cheek/jaw area, showing a shaved or stubbled patch of skin with multiple erythematous (red to reddish-purple) papules and plaques. The lesions appear inflamed and are clustered primarily in the lower cheek and jawline, coinciding with the area of hair growth. The skin texture surrounding the lesions is generally irritated with visible hair follicles. This presentation, characterized by inflammation, redness, and papules in a hair-bearing area that has recently been shaved, is highly suggestive of Pseudofolliculitis Barbae (razor bumps) or possibly an infectious folliculitis.					
5. The image shows the skin of the lower cheek/jaw area with close-cropped hair growth (stubble). The skin exhibits multiple erythematous (red to reddish-					

purple), somewhat papular or slightly raised lesions scattered across the area. The surface texture appears mildly irregular and irritated, possibly with some post-inflammatory hyperpigmentation (darker spots) mixed with the redness. The distribution is mainly limited to the beard area. This presentation is highly suggestive of pseudofolliculitis barbae (razor bumps) or possibly folliculitis, which is inflammation of the hair follicles, often caused by ingrown hairs or shaving irritation in individuals with curly or coarse hair.					
6. The image displays a close-up of a man's cheek and jawline, characterized by numerous, poorly defined erythematous (red to purplish-red) papules and macules scattered across the beard area. The texture appears slightly bumpy and inflamed over the stubble, which indicates a follicular process. The distribution is localized to the beard-growing skin of the lower cheek. These features, involving inflammation of the hair follicles exacerbated by shaving, are highly suggestive of Pseudofolliculitis Barbae (razor bumps), although other follicular inflammatory conditions like bacterial folliculitis or irritant contact dermatitis could be considered.					
1. L'image présente une zone de la joue et de la mâchoire couverte de follicules pileux (barbe naissante) sur laquelle on observe de multiples papules et petites plaques érythémateuses à violacées (rouges à rouge-pourpre). Les lésions sont surélevées et de taille variable, certaines semblant regroupées ou confluentes, donnant à la peau une texture irrégulière et inflammée. La distribution est localisée sur une zone de rasage. Cette présentation est fortement suggestive d'une Pseudofolliculite de la barbe (poils incarnés), une affection inflammatoire courante dans les zones de pilosité rasée, mais pourrait également correspondre à une acné inflammatoire (papulo-pustuleuse) ou une folliculite bactérienne.					
2. L'image clinique montre la peau du visage d'un homme au niveau de la mâchoire et de la joue, caractérisée par une éruption de lésions érythémateuses et papuleuses qui semblent être inflammatoires et douloureuses ou prurigineuses (démangeaisons). Ces lésions sont distribuées dans la zone de la barbe et se manifestent par des taches rouges à violacées de taille variable, certaines présentant un aspect légèrement surélevé ou nodulaire. La peau environnante montre un poil					

court (barbe de quelques jours). La distribution et les caractéristiques des lésions suggèrent fortement un diagnostic de folliculite de la barbe (Sycosis barbae), qui est une inflammation des follicules pileux, souvent causée par une infection bactérienne (staphylocoque) ou une irritation liée au rasage.					
3. D'après l'image clinique, on observe une éruption cutanée au niveau de la région de la barbe (zone mentonnière et joues) d'un homme. Les lésions se présentent sous forme de papules et de pustules érythémateuses (rougeâtres), dont certaines semblent plus profondes et violacées, suggérant une inflammation et potentiellement des kystes ou des nodules. Ces lésions sont polymorphes (plusieurs types de lésions coexistantes) et ont une distribution focale, principalement dans la zone de rasage. La texture de la peau environnante semble être celle d'une peau rasée ou avec une barbe naissante, ce qui pourrait être un facteur déclenchant ou aggravant. Le diagnostic possible le plus probable est la folliculite de la barbe (sycosis barbae), souvent une folliculite bactérienne (ex : Staphylococcus aureus), ou une forme inflammatoire d'acné vulgaire dans cette zone, éventuellement exacerbée par le rasage.					
4. Sur cette image clinique, on observe une peau d'homme au niveau de la région de la barbe, présentant une éruption cutanée caractérisée par de multiples papules et plaques érythémateuses (rouges) et parfois violacées (discrètement bleutées), de tailles variées. La distribution semble être péri-folliculaire (autour des poils de la barbe) et localisée sur cette zone du visage. La peau apparaît irritée avec une texture potentiellement légèrement surélevée au niveau des lésions. Compte tenu de l'aspect et de la localisation, le diagnostic le plus probable à considérer est la folliculite de la barbe (ou sycosis barbae), qui est une inflammation des follicules pileux souvent causée par une infection bactérienne ou une irritation liée au rasage.					
5. L'image clinique montre une zone de la joue et de la mâchoire d'un homme recouverte de poils de barbe courts, présentant de multiples lésions inflammatoires. Ces lésions sont principalement érythémateuses à rouge-violacé, se manifestant comme des papules et des macules. Elles sont parfois regroupées ou légèrement surélevées. La distribution est caractéristique de la zone de rasage (région de la barbe). La texture semble présenter une irritation et une induration légère à modérée autour des follicules pileux. Cette présentation est					

fortement évocatrice d'une Pseudofolliculitis Barbae (PFB), une réaction inflammatoire chronique causée par la pénétration du poil rasé et courbé dans la peau, fréquente chez les personnes ayant des poils bouclés. D'autres diagnostics à considérer seraient une folliculite bactérienne ou un acné inflammatoire, mais la PFB est la principale hypothèse clinique vu la localisation et l'aspect des lésions chez un homme qui se rase.					
6. L'image présente une zone de peau (vraisemblablement la joue ou la mâchoire d'un homme à en juger par la présence de poils de barbe courts) affichant des lésions érythémateuses (rougeâtres à violacées) de différentes tailles. Les lésions sont principalement des papules ou des plaques inflammatoires, certaines semblant légèrement surélevées. La texture générale de la peau semble comporter des bosses et des irrégularités. La distribution des lésions est diffuse dans la zone visible. Ces caractéristiques cliniques sont fortement suggestives d'une folliculite (inflammation des follicules pileux), qui est souvent une complication du rasage (connue sous le nom de pseudofolliculite de la barbe chez les hommes ayant des poils bouclés) ou d'une infection bactérienne (folliculite bactérienne), ou il pourrait également s'agir d'une acné inflammatoire de degré modéré à sévère.					
1. Βλέπω μια εικόνα που δείχνει μια περιοχή δέρματος, πιθανώς στο πρόσωπο ενός άνδρα, με εμφανείς δερματικές βλάβες. Παρατηρούνται ερυθρές έως ιώδεις κηλίδες και βλατίδες, ορισμένες εκ των οποίων φαίνονται να είναι φλεγμονώδεις και ελαφρώς ανασηκωμένες. Η κατανομή των βλαβών είναι διάσπαρτη στην περιοχή που καλύπτει ο κοντινός φακός, η υφή του δέρματος είναι εμφανώς ερεθισμένη και ανομοιόμορφη και φαίνεται να υπάρχει και πρόσφατη ανάπτυξη τριχών (γένια). Αυτή η κλινική εικόνα είναι συμβατή με φλεγμονώδη ακμή (π.χ., βλατιδοφλυκταινώδης ακμή), ή με θύλακίτιδα (φλεγμονή των τριχοθυλακίων), η οποία μπορεί να επιδεινώνεται από το ξύρισμα (ψευδοθυλακίτιδα γενείου).					
2. Με βάση την κλινική εικόνα, παρατηρείται δέρμα προσώπου με κοντή τριχοφυΐα (γένια/αξύριστο), το οποίο εμφανίζει πολλαπλές φλεγμονώδεις βλάβες. Το χρώμα των βλαβών είναι κυρίως ερυθηματώδες (κόκκινο), με ορισμένες μεγαλύτερες και βαθύτερες εστίες να παρουσιάζουν μια πιο μωβ ή					

μελαχρωματική απόχρωση. Η υφή χαρακτηρίζεται από βλατίδες και μικρές πλάκες που είναι ελαφρώς επήρμενες, υποδηλώνοντας φλεγμονή. Η κατανομή είναι πολυεστιακή και εντοπίζεται αποκλειστικά στην περιοχή όπου αναπτύσσονται οι τρίχες. Η εμφάνιση αυτών των ερυθηματωδών, φλεγμονωδών βλατίδων σε περιοχή με τριχοφυΐα μετά από ξύρισμα καθιστά την ψευδοθυλακίτιδα της γενειάδας (Pseudofolliculitis barbae) την πιθανότερη διάγνωση.					
3. Στην κλινική εικόνα παρατηρείται δέρμα στην περιοχή της γενειάδας (πιθανότατα μάγουλο και σαγόνι) που φέρει ερυθηματώδεις βλάβες (κοκκινίλες). Οι βλάβες αυτές είναι κυρίως βλατίδες (μικρά ανυψωμένα σπυράκια) και σε ορισμένα σημεία εμφανίζονται ως συρρέουσες (ενωμένες) και πιο βαθιές φλεγμονώδεις περιοχές με πορφυρό έως σκούρο κόκκινο χρώμα, υποδηλώνοντας βαθύτερη φλεγμονή ή μεταφλεγμονώδη υπερμελάγχρωση. Η κατανομή των βλαβών είναι διάσπαρτη αλλά συγκεντρωμένη στην περιοχή ανάπτυξης των τριχών. Η υφή του δέρματος φαίνεται να είναι ερεθισμένη με έντονη κοκκινίλα γύρω από τους θύλακες των τριχών. Η εικόνα είναι ιδιαίτερα ενδεικτική για θυλακίτιδα ή ψευδοθυλακίτιδα γενειάδας (Folliculitis barbae ή Pseudofolliculitis barbae), συνήθως σχετιζόμενη με το ξύρισμα, όπου οι τρίχες είτε μολύνονται είτε αναπτύσσονται προς τα μέσα προκαλώντας φλεγμονώδη αντίδραση.					
4. Στην εικόνα παρατηρείται δέρμα προσώπου (μάλλον στην περιοχή της παρειάς/γένειας ενός άνδρα), καλυμμένο με κοντό τρίχωμα. Το κύριο εύρημα είναι η παρουσία πολλαπλών ερυθηματωδών (κόκκινων/ροζ) βλαβών με ποικίλη διαμόρφωση και μέγεθος. Ορισμένες βλάβες φαίνονται ως διηθημένα, ερυθηματώδη οζίδια ή πλάκες, με ορισμένα σημεία να έχουν μια πιο πορφυρική ή υπομελανωτική/μεταφλεγμονώδη απόχρωση. Οι βλάβες είναι συγκεντρωμένες στην περιοχή του γενείου και φαίνεται να σχετίζονται με την ανάπτυξη των τριχών. Η υφή του δέρματος φαίνεται να είναι τραχιά ή ανομοιόμορφη στις πληγείσες περιοχές. Η κατανομή των βλαβών στην περιοχή του ξυρίσματος και τα χαρακτηριστικά τους είναι συμβατά με φλεγμονώδεις καταστάσεις των τριχοθυλακίων, όπως η Ψευδοθυλακίτιδα της Γένεια (Pseudofolliculitis Barbae) ή μια μορφή θυλακίτιδας.					
5. Η εικόνα δείχνει το δέρμα στην περιοχή του προσώπου και συγκεκριμένα στο κάτω μέρος της παρειάς (μάγουλο) ενός άνδρα με κοντή γενειάδα/τριχοφυΐα. Παρατηρούνται πολλαπλές					

ερυθρο-ιώδεις βλατίδες και οζίδια (μικρά εξογκώματα), ορισμένα από τα οποία φαίνονται να είναι επιφανειακά και άλλα βαθύτερα, με ελαφρώς πρησμένη υφή. Η κατανομή των βλαβών είναι διάσπαρτη, αλλά με τάση να σχηματίζουν συστάδες, κυρίως στην περιοχή που καλύπτεται από τα γένια (περιοχή ξυρίσματος). Τα χαρακτηριστικά αυτά, που συνδυάζουν ερυθρότητα, φλεγμονώδεις βλάβες και την κατανομή στην περιοχή των τριχοθυλακίων, είναι συμβατά με διάγνωση ψευδοθυλακίτιδας της γενειάδας ή θυλακίτιδας.					
6. Βλέπω μια εικόνα που απεικονίζει το δέρμα στο πρόσωπο ενός άνδρα, πιθανότατα στην περιοχή του μάγουλου και του σαγονιού, το οποίο παρουσιάζει πολλαπλές ερυθηματώδεις (κόκκινες) βλατίδες και πλάκες με διαφόρους βαθμούς φλεγμονής και, σε ορισμένα σημεία, με ελαφρώς ιώδες (μπλε-μωβ) χρωματισμό (πιθανώς μεταφλεγμονώδης υπερμελάγχρωση ή βαθύτερη φλεγμονή). Η κατανομή φαίνεται να είναι διάσπαρτη στην περιοχή της γενειάδας, με την υφή να είναι ανώμαλη λόγω των εξογκωμάτων. Τα ευρήματα αυτά, ιδιαίτερα η φλεγμονή στην περιοχή του ξυρίσματος και η παρουσία βλατίδων, είναι έντονα ενδεικτικά θυλακίτιδας, και πιο συγκεκριμένα, θα μπορούσε να είναι θυλακίτιδα της γενειάδας ή βακτηριακή θυλακίτιδα, ιδιαίτερα αν συνδέεται με το ξύρισμα.					
1.<br>Basierend auf dem klinischen Bild ist eine Hautirritation im Bereich des Bartwuchses und der angrenzenden Wange einer männlichen Person zu sehen. Die dermatologischen Merkmale umfassen rötliche bis leicht violette (erythematöse und purpurfarbene) Flecken und Papeln. Die Textur scheint leicht erhaben und entzündlich zu sein. Die Verteilung der Läsionen ist unregelmäßig, konzentriert sich jedoch auf den Bereich, der regelmäßig rasiert wird. Die umliegende Haut weist einen kurzen Bartstoppel-Wuchs auf. Eine mögliche Diagnose für dieses Erscheinungsbild ist Pseudofolliculitis barbae, eine entzündliche Reaktion, die auftritt, wenn sich scharfe Haarenden nach dem Schneiden in die Haut zurückkrümmen und einwachsen, oder eine follikuläre Entzündung (z. B. Follikulitis), die durch Reizung, Rasur oder Infektion verursacht wird.					
2.<br>Auf diesem klinischen Bild ist die Haut eines männlichen Gesichts zu sehen, die eine Reihe von entzündlichen und erythematösen Läsionen					

aufweist, die in einem Bereich des unteren Gesichts und des Kiefers verteilt sind, in dem ein kurzer Bartstoppeln (Stoppeln) vorhanden ist. Die Farbe der Läsionen variiert von leuchtendem Rot (Erythem) bis hin zu tieferen, rötlich-violetten Flecken, was auf eine aktive Entzündung und möglicherweise auf eine postinflammatorische Hyperpigmentierung oder Blutergüsse hindeutet. Die Textur scheint papulös oder knötchenförmig zu sein, mit erhabenen, unebenen Bereichen. Die Verteilung ist eher diffus und patchy in den Bartbereichen. Die gesamte Präsentation ist typisch für Follikulitis oder Pseudofollikulitis Barbae (durch eingewachsene Haare), eine häufige Hauterkrankung, die durch Entzündung der Haarfollikel infolge von Rasur verursacht wird.					
3. Auf dem klinischen Bild ist die Haut eines männlichen Gesichts zu sehen, die eine Reihe von entzündlichen Läsionen aufweist, die mit einer frisch rasierten oder sehr kurzen Bartstoppel-Haut in Verbindung stehen. Die Läsionen sind überwiegend rötlich bis leicht violett (erythematös bis livid), was auf eine aktive Entzündung hinweist. Sie erscheinen als Papeln oder Pusteln (kleine, erhabene, manchmal eitergefüllte Beulen) und sind unregelmäßig im Bereich des Kiefers und der Wange verteilt, primär dort, wo die Bartstoppeln am dichtesten sind. Die Textur der betroffenen Haut ist uneben und entzündet. Diese Merkmale, insbesondere in der Bartregion, sind stark verdächtig für eine Follikulitis oder Pseudofollikulitis Barbae, also eine Entzündung der Haarfollikel, oft hervorgerufen durch Bakterien, Pilze oder eingewachsene Haare nach der Rasur					
4. Auf dem klinischen Bild ist die Wange eines Mannes mit stoppeliger Gesichtsbehaarung zu sehen, auf der sich mehrere dermatologische Läsionen befinden. Die Läsionen erscheinen als rötliche bis violette (erythematöse) Papeln und Plaques, einige mit leicht erhabener Textur. Sie sind verstreut und unregelmäßig verteilt, konzentrieren sich aber vorwiegend auf den rasierten/gestoppelten Bereich. Die Farbe und die Entzündung deuten auf einen entzündlichen Prozess hin. Eine mögliche Diagnose wäre Pseudofolliculitis Barbae oder entzündliche Akne, wobei das Erscheinungsbild im Bartbereich stark auf erstere hindeutet, eine häufige Erkrankung, bei der sich Haare nach innen kräuseln und Entzündungen und Beulen verursachen.					
5.<br>Auf dem klinischen Bild ist die Haut eines männlichen Gesichts zu sehen, die eine Reihe von					

entzündlichen Läsionen aufweist, die mit einer Pseudofolliculitis barbae (Rasurbrand) oder einer Form von follikulärer Akne vereinbar sind. Die Läsionen erscheinen als rötliche bis leicht violette (erythematöse und hyperpigmentierte) Papeln und Pusteln, die unregelmäßig und dicht im Bereich des Bartwuchses (Wange/Kiefer) verteilt sind. Die Hauttextur um die Läsionen herum erscheint leicht gereizt und rau mit sichtbaren kurzen Bartstoppeln, wobei einige entzündete Bereiche von der Rasur verschlimmert zu sein scheinen. Die Verteilung ist auf den rasierten/rasierten Bereich des Gesichts beschränkt, was die wahrscheinliche Diagnose einer Pseudofolliculitis barbae unterstützt, bei der scharfe Haare in die Haut zurückwachsen und eine Entzündungsreaktion hervorrufen.					
6. Auf dem klinischen Bild ist die Wange und der Kieferbereich eines Mannes mit relativ kurzem Bartwuchs zu sehen. Es sind multiple rötliche bis leicht violette (erythematöse und livide) Papeln und möglicherweise oberflächliche Knoten oder Plaques erkennbar, die hauptsächlich im Bereich des Bartes (perifollikulär) verteilt sind. Die Textur der Haut erscheint rau und uneben, mit entzündlichen Läsionen, die erhaben sind. Die Verteilung ist auf das barttragende Areal beschränkt. Die Merkmale, insbesondere die Entzündung und Rötung in den Bereichen der Haarfollikel, sind stark suggestiv für eine Follikulitis oder, in Anbetracht der Stärke der Entzündung und der dunkleren, livideren Läsionen, möglicherweise für eine tiefere Form wie die Pseudofolliculitis barbae (wenn die Ursache eingewachsene Haare nach der Rasur sind) oder eine Sycosis barbae (eine bakterielle Follikulitis im Bartbereich).					

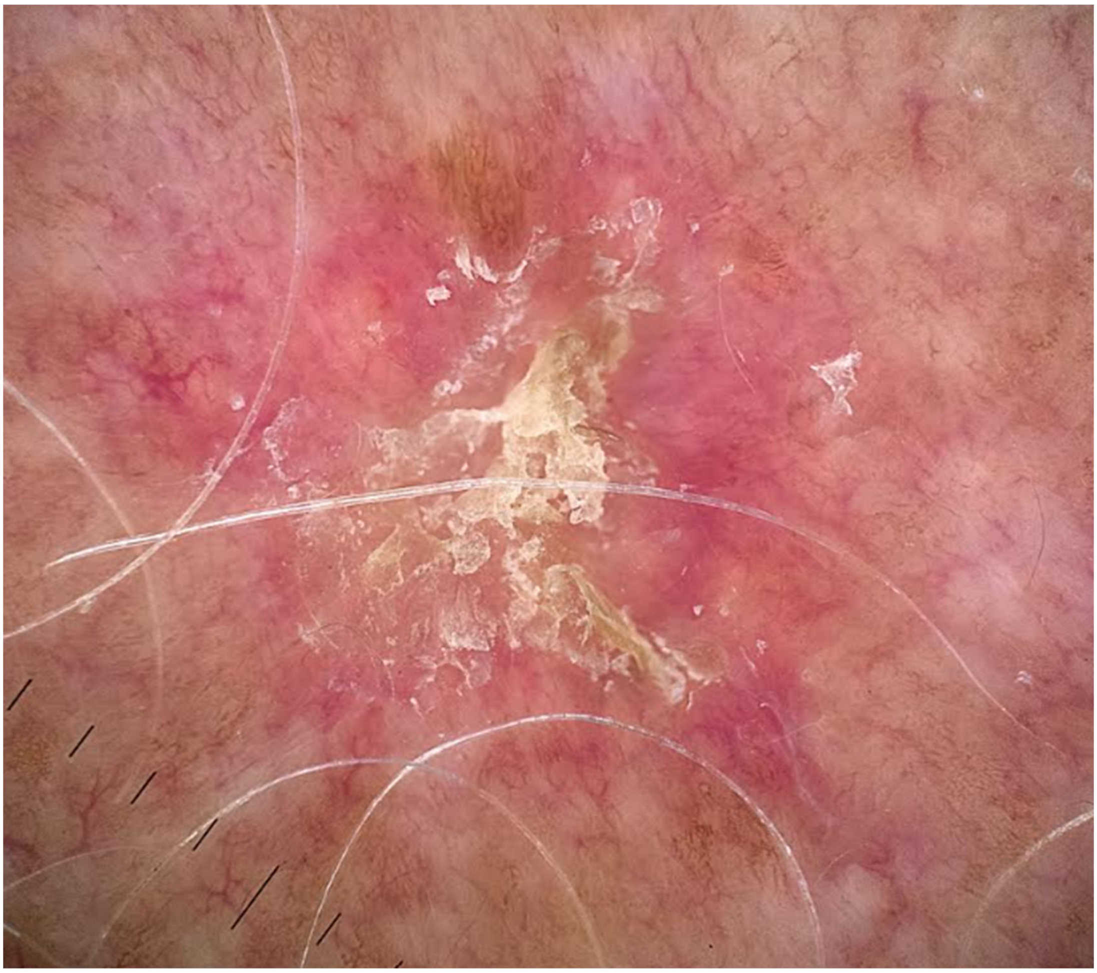

Description	Completeness	Lack of false information (accuracy)	Evidence-based content	Appropriateness	Relevance
1. The dermoscopic image shows a prominent pink-reddish background suggesting increased vascularity or inflammation, characteristic of an amelanotic lesion. Centrally, there is a large, irregular area of milky-white to yellowish structureless change with overlying fine white scales and potential crusts, which could represent regression structures, scar-like areas, or hyperkeratosis. While the vascular pattern is not distinctly visible throughout the center, the overall appearance of an amelanotic lesion with atypical pink-white structures, scales, and possible vessels warrants a high suspicion for malignancy, such as Amelanotic Melanoma or possibly an Amelanotic Basal Cell Carcinoma or Squamous Cell Carcinoma.					
2. The dermoscopic image shows a prominent reddish-pink background punctuated by a central polymorphous or whitish-yellow/tan structure characterized by scaling or crusting. The periphery displays a diffuse erythematous					

pattern with fine, linear, telangiectatic or comma-shaped vessels suggesting a non-melanocytic lesion. The borders appear somewhat ill-defined and gradually fade into the surrounding skin. This overall presentation, with the mix of erythema, scaling, and specific vascular patterns, is highly suggestive of a Basal Cell Carcinoma (BCC), possibly the superficial or nodular type, though other possibilities like inflammation or specific benign lesions must be considered.					
3. Based on the dermoscopic image, I observe a reddish-pink background which is characteristic of erythema (inflammation). Within this erythematous area, there is a central white-yellowish, scaly, and crusty structure that appears amorphous or somewhat fern-like (leaf-like). The pattern is generally irregular and polymorphous, lacking a clear, organized pigment network or distinct border. The reddish background color is due to vessel dilation (erythema), and the white-yellowish material likely represents hyperkeratosis and scaling/crusting. These features—erythema, scaling, and an irregular structure—are highly suggestive of a non-melanocytic inflammatory or benign epithelial proliferation, with Psoriasis or Lichen Planus being among the possible inflammatory diagnoses, or potentially a Basal Cell Carcinoma or Squamous Cell Carcinoma given the prominent scale/crust, although the image lacks classic malignant vascular or pigment features.					
4. Based on the dermoscopic image, I observe a polymorphous vascular pattern characterized by significant erythema (redness), indicating inflammation. The lesion shows an irregular, ill-defined border and a central area of yellowish-white scaling and crusting, which may represent serocrusts and hyperkeratosis. There are also areas of brownish-yellow pigmentation or remnants of pigment in the periphery. The overall features—including the prominent inflammation, scaling/crusting, and irregular structure—are highly suggestive of an inflammatory or infectious dermatosis, with Psoriasis, Lichen Planus-like Keratosis (LPLK), or a Dermatophytosis (fungal infection) being among the possible differential diagnoses, requiring clinical correlation and potentially a biopsy for definitive diagnosis					
5. This dermoscopic image prominently features an extensive, diffuse pink-to-red erythema, indicating substantial inflammation and vascularity, surrounding a central, ill-defined, structureless white-to-yellowish area of scaling or crust. The overall pattern is non-melanocytic and appears irregular, lacking typical pigment networks or specific vascular structures (though the redness implies increased vessels). This combination of severe erythema and central crust/hyperkeratosis is highly suggestive of a non-melanocytic skin neoplasm or a prominent inflammatory condition, with Actinic Keratosis (particularly hypertrophic) or Squamous Cell Carcinoma being key considerations.					

6. This dermoscopic image displays a lesion characterized by a central polymorphic whitish-yellowish area of scaling and crusting, which obscures the underlying structures. This central focus is surrounded by a prominent, asymmetric pink-to-reddish erythema, indicating inflammation. While the borders are generally ill-defined due to the inflammatory halo, the overall pattern is non-melanocytic. The findings are highly suggestive of an inflammatory or infectious dermatosis, such as eczema, psoriasis, or potentially an actinic keratosis with inflammation, rather than a pigmented skin cancer like melanoma.					
1. Cette image dermoscopique révèle une lésion cutanée caractérisée principalement par un érythème diffus (couleur rouge vif et rosé) suggérant une inflammation importante. Au centre, on observe des squames épaisses, blanchâtres à jaunâtres, avec un aspect croûteux ou desquamant et une possible érosion sous-jacente. Les contours de la lésion sont mal définis et irréguliers, se fondant progressivement dans la peau environnante. La vascularisation n'est pas clairement détaillée en termes de motifs spécifiques (comme des points ou des vaisseaux en virgule), mais l'érythème généralisé implique une forte composante vasculaire. Ce tableau clinique et dermoscopique, combinant érythème et squames/croûtes, est compatible avec une dermatose inflammatoire telle qu'un lupus érythémateux discoïde actif ou une autre forme de dermatose érythémato-squameuse.					
2. C'est une image dermoscopique centrée sur une lésion cutanée qui se présente principalement comme une zone érythémateuse (rouge) diffuse, suggérant une inflammation ou une augmentation du flux sanguin. Au centre, on observe une desquamation (écailles blanchâtres/jaunâtres) irrégulière et des croûtes/squames, évoquant un processus d'exfoliation ou de guérison. Le motif général est plutôt non-spécifique ou polymorphe. Les bords de la lésion ne sont pas clairement définis et se fondent dans la peau environnante. La présence d'érythème et de squames centrales est fréquemment observée dans les lésions inflammatoires ou infectieuses telles que la dermatite (eczéma, psoriasis) ou des tumeurs non-mélanocytaires superficielles (comme un carcinome basocellulaire superficiel, bien que moins typique sans autres structures vasculaires spécifiques ou pigmentaires). Un diagnostic différentiel précis nécessiterait une évaluation clinique et histologique.					
3. L'image dermoscopique révèle une lésion non-pigmentée (amélanotique), dominée par un fond érythémateux intense de couleur rose-rouge, témoignant d'une inflammation ou d'une vascularisation marquée. Au centre, on observe une zone de croûtes épaisses et irrégulières, de couleur jaune-blanchâtre, potentiellement associées à des squames et/ou une érosion. Bien que difficiles à caractériser en raison de					

l'inflammation globale, l'arrière-plan érythémateux peut contenir des structures vasculaires (vaisseaux fins ou en points) qui sont courantes dans les lésions non-pigmentées. L'absence de structures pigmentaires classiques (réseau, globules) et la présence d'une inflammation et d'une desquamation croûteuse/hyperkératose rendent ce tableau compatible avec plusieurs diagnostics, notamment une Kératose Actinique inflammatoire, une maladie inflammatoire comme l'eczéma, ou une tumeur épithéliale amélanotique comme un Carcinome Basocellulaire superficiel ou un Carcinome Épidermoïde, nécessitant une évaluation clinique et histologique					
4. L'image présente une lésion cutanée caractérisée par un fond majoritairement rouge-rose vif diffus, signe d'un érythème important. Au centre, on note une zone blanchâtre/jaunâtre de squames épaisses et de croûtes, suggérant une hyperkératose et/ou une érosion. Le motif vasculaire est prédominant, constitué de vaisseaux fins, courts et linéaires ou en "point d'interrogation" répartis de manière irrégulière. L'absence de réseau pigmentaire ou de structures mélanocytaires est notable. Ces caractéristiques dermoscopiques, notamment l'érythème et les vaisseaux linéaires irréguliers associés à des squames/croûtes, sont hautement suspectes d'une tumeur cutanée non mélanocytaire maligne, tel un Carcinome Basocellulaire superficiel achromique ou une Maladie de Bowen (Carcinome épidermoïde in situ).					
5. Cette image dermoscopique présente une lésion cutanée caractérisée par un fond érythémateux (rouge vif à rose) qui domine le cliché, indiquant une inflammation et une forte vascularisation. Au centre, on observe des squames de couleur blanc-jaunâtre, poudreuses et adhérentes. Le motif vasculaire est principalement composé de vaisseaux en tire-bouchon (ou vaisseaux glomérulaires) et de vaisseaux linéaires irréguliers, visibles sur le fond érythémateux, ainsi que des lignes blanches et brillantes (ou stries blanches/cristallines) qui se croisent, suggérant une fibrose ou une prolifération épidermique. Les contours de la lésion sont irréguliers et mal définis. Ces caractéristiques (érythème, squames centrales, vaisseaux en tire-bouchon) sont fortement suggestives d'une kératose actinique hyperkératosique ou d'un carcinome épidermoïde in situ (Maladie de Bowen), le motif vasculaire et les structures blanches étant des signes d'inquiétude.					
6.<br>L'image dermoscopique révèle une zone centrale marquée par un fond érythémateux intense, indiquant une inflammation. Au centre de cette zone rouge, on observe une desquamation (squames) de couleur blanc-jaunâtre et une érosion ou une croûte jaunâtre, suggérant une rupture de la surface cutanée. Le motif vasculaire est caractérisé par					

la présence de vaisseaux linéaires et irréguliers sur le fond érythémateux, ainsi que potentiellement des structures vasculaires plus diffuses ou polygonales, typiques des troubles inflammatoires. L'ensemble de ces signes (érythème, squames/croûtes, et vaisseaux irréguliers) est hautement suggestif d'une dermatose inflammatoire du visage, et en particulier, d'une forme de rosacée papulo-pustuleuse ou d'une autre dermatose érythémato-squameuse comme la dermatite séborrhéique ou un eczéma facial, nécessitant une évaluation clinique pour un diagnostic définitif.					
1.Η δερματοσκοπική εικόνα παρουσιάζει μια ασαφώς οριοθετημένη βλάβη, η οποία κυριαρχείται από έντονο ερύθημα (κόκκινο/ροζ φόντο), υποδηλώνοντας φλεγμονή και αυξημένη αγγείωση. Στο κέντρο της βλάβης παρατηρείται μια υπόλευκη έως κιτρινωπή άμορφη δομή με πλακώδη ή λεπιδώδη στοιχεία και κρούστες/εφελκίδες, πιθανόν λόγω υπερκεράτωσης, απολέπισης ή εξιδρώματος. Επίσης, φαίνεται να υπάρχουν λεπτά λευκά ινώδη δοκίδια/γραμμές (λευκές λαμπερές δομές) και αγγειακές δομές που δεν είναι σαφώς διακλαδιζόμενες, αλλά μάλλον σημειακές ή γραμμοειδείς (στικτά ή γραμμοειδή αγγεία) διάσπαρτα στο ερυθρό φόντο. Η απουσία τυπικού μελανοκυτταρικού δικτύου και η κυριαρχία του ερυθήματος και των λευκών/κιτρινωπών δομών παραπέμπουν σε φλεγμονώδη δερματοπάθεια (π.χ., έκζεμα, ψωρίαση, ή ακανθοσμίαση) ή σε μη μελανοκυτταρική νεοπλασία (π.χ., πρώιμο βασικοκυτταρικό καρκίνωμα					
2. Η δερματοσκοπική εικόνα παρουσιάζει ένα έντονα ερυθηματώδες (κόκκινο/ροζ) υπόβαθρο, το οποίο υποδηλώνει έντονη φλεγμονή. Κυρίαρχο στοιχείο είναι η παρουσία λευκωπών έως κιτρινωπών εφελκίδων και λεπίων που καλύπτουν το κεντρικό τμήμα της βλάβης, δημιουργώντας μία ακανόνιστη, ασαφή δομή. Επίσης, παρατηρούνται αγγειακές δομές (πιθανώς διάσπαρτες κόκκινες κουκκίδες ή άτυπα αγγεία) που ενισχύουν το ερύθημα. Τα όρια της βλάβης είναι ασαφή και σταδιακά μεταβαίνουν στο γύρω φυσιολογικό δέρμα. Ο συνδυασμός ερυθήματος, λεπιών/εφελκίδων και αγγειακών δομών είναι χαρακτηριστικός φλεγμονωδών δερματοπαθειών (φλεγμονωσκόπηση), με πιθανή διάγνωση έκζεμα (δερματίτιδα) ή σμηγματορροϊκή δερματίτιδα.					
3. Η παρεχόμενη δερματοσκοπική εικόνα απεικονίζει μια ερυθηματώδη βλάβη (έντονο κόκκινο/ροζ φόντο) με ασαφή και ανομοιόμορφα όρια. Στο κέντρο της βλάβης κυριαρχεί ένα λευκωπό/κιτρινωπό άμορφο υλικό που υποδηλώνει έντονη απολέπιση ή υπερκεράτωση, πιθανώς και έλκος ή νέκρωση σε ορισμένες περιοχές, καθώς και κηλίδες/δομές καστανού χρώματος. Το αγγειακό μοτίβο, αν και δεν είναι					

απόλυτα ευδιάκριτο, φαίνεται να είναι πυκνό και μη ειδικό (π.χ., γραμμοειδή ή διάσπαρτα αγγεία), το οποίο συνεισφέρει στον έντονο ερυθρό τόνο. Η έλλειψη τυπικού μελαγχρωματικού δικτύου ή άλλων ειδικών μελαγχρωματικών δομών, σε συνδυασμό με το ερύθημα, την απολέπιση/υπερκεράτωση και τα ασαφή όρια, υποδηλώνει μια μη μελαγχρωματική βλάβη. Η εικόνα είναι συμβατή με διάφορες δερματικές παθήσεις, αλλά τα χαρακτηριστικά αυτά συχνά παρατηρούνται σε ενεργό φλεγμονώδη ή υπερπλαστική διεργασία, όπως Βασικοκυτταρικό Καρκίνωμα (ιδίως το επιφανειακό ή υπερκερατωσικό του υποτύπου), Ακανθοκυτταρικό Καρκίνωμα ή σοβαρή Ακτινική Κεράτωση / Νόσος του Bowen. Χρειάζεται βιοψία για οριστική διάγνωση.					
4. Η δερματοσκοπική εικόνα παρουσιάζει μια ερυθηματώδη (κόκκινη) βλάβη με ασαφή και ακανόνιστα όρια. Στο κέντρο, κυριαρχεί μια λευκοκίτρινη-καφέ, άμορφη δομή που υποδηλώνει πλάκες (κρούστα) και απολέπιση/νέκρωση της επιδερμίδας. Παρατηρείται ένα ευρύ ερυθηματώδες υπόστρωμα με γραμμοειδείς/ευθεία αγγεία διατεταγμένα στο περιφερικό τμήμα (τηλεαγγειεκτασίες) και πιθανώς λευκό-ροζ πέπλο (white-pink veil) ή πρόσφατη ουλοποίηση σε κάποια σημεία. Το κυρίαρχο μοτίβο είναι μη ειδικό και φλεγμονώδες/αγγειακό, με στοιχεία επιφανειακής καταστροφής. Η κλινικο-δερματοσκοπική εικόνα, με την έντονη ερυθρότητα και την κεντρική νέκρωση/απολέπιση, είναι ενδεικτική μιας φλεγμονώδους, διαβρωτικής ή εξελκωτικής διεργασίας, και θα μπορούσε να αντιστοιχεί σε μια ευρεία γκάμα διαγνώσεων, συμπεριλαμβανομένων φλεγμονωδών δερματοπαθειών, ακτινικών βλαβών, ή δυνητικά κακοήθων νεοπλασμάτων όπως καρκίνωμα πλακωδών κυττάρων (SCC) ή βασικοκυτταρικό καρκίνωμα (BCC) με εξέλκωση. Απαιτείται ιατρική εκτίμηση και βιοψία για την οριστική διάγνωση.					
5. Η παρεχόμενη δερματοσκοπική εικόνα παρουσιάζει μια βλάβη με έντονο ερύθημα (κόκκινο/ροζ φόντο) και ακανόνιστα, ασαφή όρια. Κυριαρχεί ένα πολυμορφικό αγγειακό μοτίβο, χαρακτηριζόμενο από εμφανή αγγεία, συχνά διατεταγμένα σε γραμμές ή θύσακες, που υποδηλώνουν αυξημένη αγγείωση και φλεγμονή. Στο κέντρο της βλάβης παρατηρείται μια περιοχή με λευκοκιτρινωπά λέπια και/ή κρούστα, υποδηλώνοντας υπερκεράτωση και ίσως εξέλκωση ή έντονη απολέπιση. Συνολικά, η εικόνα είναι συμβατή με ένα μη μελανοκυτταρικό νεόπλασμα ή μια έντονα φλεγμονώδη κατάσταση. Λόγω του ερυθήματος, των ακανόνιστων αγγειακών δομών και της κεντρικής απολέπισης/λέπιασης, η εικόνα είναι ισχυρά ενδεικτική πιθανής Βασικοκυτταρικής Καρκινομάτωσης, ιδίως της επιφανειακής ή οζώδους μορφής, αν και θα μπορούσε επίσης να αντιπροσωπεύει Ακανθοκυτταρικό Καρκίνωμα ή μια φλεγμονώδη					

δερματοπάθεια. Η τελική διάγνωση απαιτεί πάντα ιστοπαθολογική εξέταση.					
6. Η δερματοσκοπική εικόνα παρουσιάζει μια βλάβη που χαρακτηρίζεται από έντονη ερυθρότητα στο φόντο, υποδηλώνοντας φλεγμονή ή αυξημένη αγγείωση. Κεντρικά παρατηρείται μια περιοχή με λευκωπό-κιτρινωπό χρώμα και λεπτή απολέπιση/κρούστα, η οποία μπορεί να αντιστοιχεί σε επιφανειακή νέκρωση, ίνωση, ή έντονη υπερκεράτωση. Διακρίνονται ευκρινή, αλλά ακανόνιστα όρια, με τη βλάβη να περιβάλλεται από το ερύθημα. Το αγγειακό μοτίβο, αν και δυσδιάκριτο λόγω του έντονου ερυθήματος, μπορεί να περιλαμβάνει διάχυτα, λεπτά αγγεία. Η συνολική εικόνα, με το ερύθημα, την απολέπιση/κρούστα και την κεντρική λευκωπή/κιτρινωπή αλλοίωση, είναι ενδεικτική για μη μελανωματικούς όγκους, όπως ένα βασικοκυτταρικό καρκίνωμα (ιδίως το επιφανειακό ή το ινώδες) ή πιθανώς μια ακτινική κεράτωση/νόσος Bowen με φλεγμονώδη αντίδραση, αλλά απαιτείται ιστοπαθολογική επιβεβαίωση για την τελική διάγνωση.					
1. Auf dem dermatoskopischen Bild ist eine unscharf begrenzte Läsion zu erkennen, die primär durch eine intensive Rötung (Erythem) des Hintergrunds dominiert wird. Zentral fällt eine auffällige, weißlich-gelbliche Struktur auf, die schuppig, krustig oder hyperkeratotisch erscheint und sich schuppenartig und unregelmäßig über einen Teil der Läsion erstreckt. In der Peripherie und um die zentralen Veränderungen herum sind lineare, gewundene oder verzweigte Gefäße zu sehen, die typisch für entzündliche oder infiltrative Prozesse sein können, wie sie beispielsweise beim Lupus erythematodes (subakut oder diskoid), Rosazea oder Lichen planus (Wickham-Streifen-ähnliche weiße Linien) beobachtet werden. Die Kombination aus starker Rötung, unregelmäßiger weißlicher Schuppung/Hyperkeratose und linearen Gefäßen ist kennzeichnend für eine entzündliche Dermatose. Eine spezifische Diagnose, wie z.B. Lichen planus (wegen der potenziellen Ähnlichkeit zur Wickham-Streifung) oder Lupus erythematodes (wegen der linearen Gefäße und Erythem), müsste durch Korrelation mit dem klinischen Bild und einer Biopsie bestätigt werden.					
2. Auf diesem dermatoskopischen Bild ist eine entzündlich erscheinende Läsion zu erkennen. Die vorherrschenden Merkmale sind ein diffuses, rotes bis rosa-rotes Erythem als Hintergrund. Zentral dominiert eine weißlich-gelbliche, unstrukturierte, krustöse bis schuppende Auflagerung, die einen Großteil des Zentrums bedeckt. An der Peripherie und im umgebenden Erythem sind feine, weiße, lineare, geschwungene Linien erkennbar, die ansatzweise ein netzartiges oder retikuläres Muster bilden. Die Ränder der					

gesamten Läsion erscheinen eher unscharf und diffus. Diese Kombination aus Erythem, Schuppen/Krusten und feinen weißen Linien (vergleichbar mit Wickham-Streifen) ist charakteristisch für entzündliche Dermatosen (Inflammoskopie) und kann insbesondere bei Lichen ruber planus, aber auch bei anderen schuppenden Hauterkrankungen (z. B. Ekzemen) beobachtet werden.					
3.Auf diesem dermatoskopischen Bild ist eine Läsion zu sehen, die durch ein prominentes erythematöses Hintergrundmuster mit diffus verteilten rötlichen Bereichen gekennzeichnet ist, was auf eine erhöhte Vaskularisierung oder Entzündung hindeutet. Im Zentrum der Läsion befindet sich eine unstrukturierte, gelblich-weiße bis bräunliche Region, die möglicherweise eine Kruste, Schuppung oder Regression darstellt. Es sind auch feine weiße Linien zu erkennen, die über die gesamte Läsion verlaufen, was Artefakte (z.B. Haar oder Fussel) oder möglicherweise dermatoskopische Strukturen (wie Schuppung oder feine Fibrose) sein könnten. Atypische Gefäße oder klare Pigmentnetzwerke sind nicht offensichtlich. Die unscharfen, unregelmäßigen Ränder der Läsion und die allgemeine entzündliche Erscheinung sind Merkmale, die bei entzündlichen Hauterkrankungen (z. B. Ekzem, Psoriasis) oder bestimmten nicht-melanozytären Hautkrebsarten wie dem Plattenepithelkarzinom (PEK) in Betracht gezogen werden müssen, insbesondere wenn die zentralen Veränderungen auf Erosion oder Krustenbildung hindeuten. Eine definitive Diagnose ist ohne klinische Korrelation nicht möglich.					
4. Dieses dermatoskopische Bild zeigt eine unscharf begrenzte, erythematöse (rötliche) Läsion, die zentral weiß-gelbliche Schuppen oder Krusten aufweist. Das vorherrschende Muster ist ein strukturloses Erythem, was auf eine Entzündung und erweiterte Gefäße in der Dermis hindeutet. Im zentralen Bereich sind unregelmäßige weiß-gelbliche Bereiche sichtbar, die Erosionen, Verkrustungen oder Hyperkeratose darstellen könnten, typisch für eine Schädigung oder chronische Entzündung der Epidermis. Ein klassisches Pigmentnetzwerk oder klare melanozytäre Strukturen fehlen, was auf eine nicht-melanozytäre Läsion hindeutet. Die unscharfen Ränder und die Kombination aus Erythem und Schuppung/Kruste lassen an entzündliche Hauterkrankungen (Dermatitiden) oder bestimmte nicht-melanozytäre Tumore denken, wie zum Beispiel ein Superfizielles Basalzellkarzinom (baumartig verzweigte Gefäße sind hier nicht klar erkennbar, aber Erythem und Erosionen sind möglich) oder eine aktinische Keratose (Erythem, Schuppung, ggf. unscharfe Grenzen). Die Unterscheidung erfordert eine klinische Korrelation.					
5. Auf dem dermatoskopischen Bild ist eine relativ scharf begrenzte, aber insgesamt unregelmäßig konfigurierte					

Läsion zu sehen, die hauptsächlich ein prominentes, homogenes Erythem (Rötung) zeigt. Diese starke Rötung deutet auf eine ausgeprägte entzündliche Komponente hin. Zentral in der Läsion dominieren weiße, unstrukturierte Areale sowie gelblich-weiße, krustige oder schuppige Auflagerungen. Die weißen Areale werden in der Dermatoskopie häufig als "Shiny White Structures" (SWS) interpretiert, die auf Fibrose oder Regression hinweisen. Die gelblich-weiße Schuppung kann auf Hyperkeratose oder Krustenbildung hindeuten. Ein Pigmentnetzwerk oder andere spezifische Melanom- oder Nävus-Strukturen sind nicht erkennbar.<br>Zusammenfassend lassen die Strukturen auf eine regressive entzündliche Läsion schließen:<br>Farben: Dominantes Erythem (Rot), Gelblich-Weiß.<br>Muster: Strukturlos (im Hinblick auf Pigment), weiße unstrukturierte/narbenartige Areale (Regression).<br>Ränder: Können unscharf sein, aber die gesamte Läsion grenzt sich von der umliegenden Haut ab.<br>Die Kombination aus intensivem Erythem, Schuppung und Anzeichen von Regression (weiße Strukturen/Narbenbildung) ist sehr typisch für eine Lichen planus-artige Keratose (LPLK), insbesondere in einem späteren oder regressivem Stadium. Differentialdiagnostisch kommen andere entzündliche Dermatosen (z. B. Ekzem) oder auch regressive pigmentierte Neoplasien in Betracht.					
6. Das dermatoskopische Bild zeigt eine rosarote bis erythematöse (gerötete) Läsion, die den gesamten sichtbaren Hintergrund dominiert und auf eine Entzündung hinweist. Im Zentrum ist eine auffällige, unregelmäßig geformte Fläche mit weißlich-gelblichen, amorphen Schuppen oder Krusten zu sehen, die fest aufzuliegen scheinen. Die Ränder der Läsion sind unscharf und fließend in die umgebende Haut übergehend. Es sind keine eindeutigen pigmentierten Muster wie Netze oder Pseudopoden erkennbar, jedoch können vereinzelte, feine, linear verzweigte Gefäße oder punktförmige Gefäße im erythematösen Hintergrund vorhanden sein, obwohl sie aufgrund der zentralen Schuppung schwer zu beurteilen sind. Die Kombination aus einem erythematösen Hintergrund und einer weißlich-gelben Schuppung ist ein typisches Merkmal für entzündliche Dermatosen (Inflammoskopie), wobei Differenzialdiagnosen wie Ekzem/Dermatitis oder Seborrhoische Dermatitis (insbesondere mit fleckiger Verteilung der Schuppung und gelblich-rotem Farbton) oder auch andere schuppende entzündliche Erkrankungen in Betracht gezogen werden müssen.					

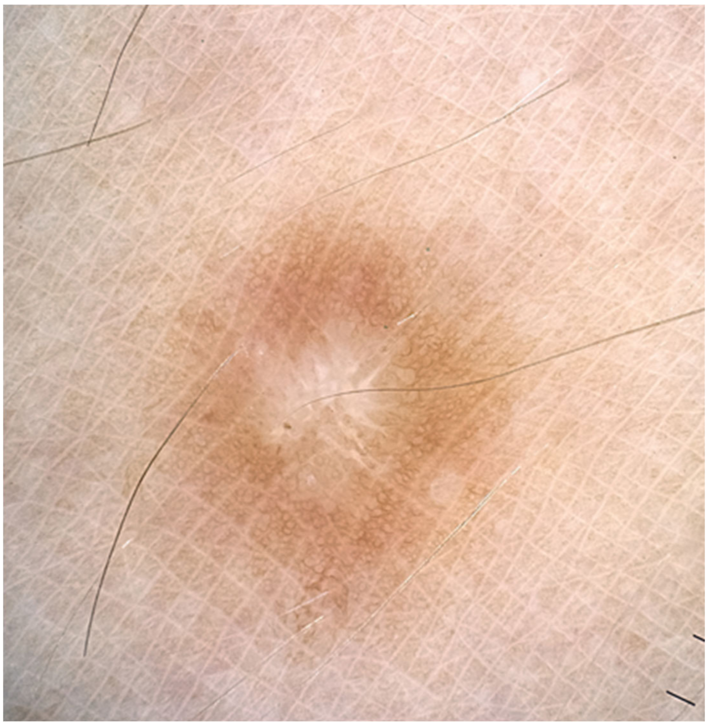

Description	Completeness	Lack of false information (accuracy)	Evidence-based content	Appropriateness	Relevance
1. Based on the dermoscopic image, I observe a light brown/tan lesion with a central area of depigmentation and a whitish, starburst-like or radial streaking pattern. The overall lesion appears relatively symmetrical and somewhat polygonal or irregular in shape, with a slightly blurry or fading border transitioning into the surrounding skin. The peripheral brown coloration often shows a fine, reticular (net-like) or pseudopod-like pattern. This presentation, particularly the central white depigmentation and peripheral pigment, is highly suggestive of a Nevus Spilus (or Speckled Lentiginous Nevus) undergoing spontaneous regression or a Halo Nevus (Sutton's Nevus). However, the radial white streaks and overall appearance can also be a finding in certain melanomas undergoing regression, making a definitive diagnosis without clinical context and professional evaluation impossible.					
2. Based on the dermoscopic image, I observe a light brown/tan macule with a distinct peripheral reticular (net-like) pattern of pigment. Centrally, there is a regressing or involuting area, characterized by a whitish, scar-like depigmentation (often referred to as white scar-like depigmentation or regression structures like pearly white areas and fine streaks/vessels), and some pinkish-red discoloration surrounding this white area, suggesting inflammation or regression activity. The borders are					

generally ill-defined where the reticular pattern fades into the surrounding skin. This combination of a peripheral reticular pattern and central regression features is a common dermoscopic presentation of a regressing nevus (melanocytic mole) or, less commonly, a regressing melanoma, making clinical correlation and close follow-up or excision necessary for definitive diagnosis.					
3. The dermoscopic image displays a pigmented skin lesion characterized by a somewhat irregular, ill-defined border with subtle pseudopods or streaks radiating outward. The predominant color is light to dark brown with a central area of whitish-blue regression structures (scar-like depigmentation and subtle pearly white chrysalis structures or scale). The overall pattern is asymmetrical and features a faint pigment network/pseudonetwork in the periphery. Given these features, particularly the presence of regression structures, asymmetry, and multiple colors/patterns, a possible differential diagnosis includes a regressing melanoma, or an evolving atypical/dysplastic nevus, which would require further clinical correlation and potentially biopsy for definitive diagnosis					
4. This dermoscopic image displays an asymmetrically pigmented lesion with several notable features. The overall color is predominantly light to medium brown with a faint pinkish-red hue centrally. The pattern is characterized by a central area exhibiting a white scar-like depigmentation (scar-like patch) with fine, radially oriented white streaks and delicate telangiectasias (fine, red vessels) suggesting regression. Surrounding this central regressive area is a faint, somewhat reticular/pseudonetwork pigment pattern, particularly at the periphery. The border is ill-defined and fades into the surrounding skin. These features—asymmetry, a brown pseudonetwork, and prominent central regression (white scar-like depigmentation, white streaks, and telangiectasias)—are highly suggestive of a regressing melanocytic nevus or, given the degree of regression, a regressing melanoma.					
5. The image displays an asymmetrically pigmented lesion with an overall tan to light-brown color, featuring a central area of hypopigmentation (white/light pink) that exhibits a prominent starburst or pseudopodal pattern of fine, radiating, whitish streaks (veiled white color) extending outward. The surrounding tan pigmentation shows a subtle, light-brown pseudonetwork at the periphery. The lesion's border is ill-defined and fades into the surrounding skin. The combined features—especially the central depigmentation with radiating structures and peripheral pseudonetwork—are classic dermoscopic					

findings consistent with a regressing melanocytic nevus (a mole undergoing spontaneous involution), which is a possible diagnosis, though other regressing lesions, including regressing melanoma, must also be considered in a differential diagnosis.					
6. In this dermoscopic image, the most prominent feature is a central whitish-to-bright white, starburst-like area with shiny white lines radiating outwards, which suggests a scar-like or fibrotic change, often referred to as white scar-like depigmentation or chrysalis structures . This central hypopigmentation is surrounded by a peripheral, delicate, brownish pigment network which fades into the normal skin. The combination of a central white area/scar-like patch and a peripheral pigment network is a highly characteristic dermoscopic pattern, most classically associated with a Dermatofibroma (a common, benign skin tumor).					
1. L'image dermoscopique révèle une lésion cutanée caractérisée par une zone centrale hypopigmentée ou cicatricielle (blanche-laiteuse) avec des structures blanches et fines suggérant une régression. Cette zone centrale est entourée d'un réseau pigmentaire brun clair à marron foncé, qui est plus dense en périphérie. Le motif du réseau semble irrégulier et parfois pseudoréticulaire ou globulaire fin dans la partie pigmentée. Les contours de la lésion ne sont pas parfaitement définis et l'on note la présence de poils, indiquant une localisation sur une peau pileuse.<br>Structures Clés et Diagnostic Possible<br>Couleurs : Blanc laiteux/cicatriciel (central), brun clair à marron foncé (périphérique).<br>Motifs : Structures de régression (voile blanc/cicatriciel, fines lignes blanches radiées) au centre, réseau ou pseudoréseau pigmentaire en périphérie.<br>Contour : Assez irrégulier, avec une transition progressive entre le centre et la périphérie.<br>Cet aspect, en particulier la présence d'une régression centrale entourée d'une pigmentation périphérique, est très évocateur d'un Naevus de Sutton (ou halo naevus), un naevus bénin en phase de régression immunitaire. Cependant, dans un contexte de lésion isolée et chez l'adulte, la régression peut également être observée dans le Mélanome, nécessitant toujours une évaluation clinique et histologique pour un diagnostic définitif.					
2. L'image dermoscopique présentée révèle une lésion pigmentée de forme globalement ovalaire ou circulaire. La lésion est caractérisée par une pigmentation brun clair à beige en périphérie, formant un réseau pigmentaire irrégulier et souvent interrompu. Au centre, on observe une zone de fibrose blanche ou de régression, de forme					

stellaire ou cicatricielle, ce qui suggère un processus de régression de la lésion. Cette zone centrale est légèrement hypopigmentée et présente des structures vasculaires fines et irrégulières ou des points blancs et bleus (ces derniers étant plus discrets). Les contours de la lésion sont flous et irréguliers. L'ensemble des caractéristiques, notamment la régression centrale blanche/stellaire, le réseau pigmentaire irrégulier en périphérie et les variations de couleur, sont hautement suggestifs d'un Mélanome en régression (Regressing Melanoma), bien que le diagnostic différentiel inclue d'autres lésions comme le nevus de Spitz ou le nevus en régression.					
3. L'image dermoscopique présente une lésion pigmentée et asymétrique, principalement de couleur brun clair à marron en périphérie. On observe une zone centrale hypopigmentée ou blanc-bleuâtre avec des structures blanches et fibreuses (probablement des voiles ou des cicatrices). Cette zone centrale est caractéristique d'une régression au sein de la lésion. En périphérie, la pigmentation présente un motif pseudo-réticulaire ou une structure réticulaire irrégulière. La combinaison de couleurs (brun, blanc/bleu) et la présence de signes de régression (zone blanc-bleu, structures cicatricielles) sont des critères dermoscopiques qui soulèvent une forte suspicion de Mélanome, potentiellement en phase de régression.					
4. L'image dermoscopique révèle une lésion cutanée caractérisée par une pigmentation principalement brune-beige en périphérie, s'estompant vers un halo blanchâtre-rosé central. La structure la plus notable est une zone de régression centrale (le halo blanchâtre) avec des stries radiales blanches fines (voile blanc ou fibrose) et une possible dépigmentation irrégulière. Le motif est globalement asymétrique en termes de couleur et de structure. La pigmentation périphérique présente un pseudo-réseau incomplet ou un aspect homogène-flou. La présence de régression et d'asymétrie, combinée à une pigmentation hétérogène, pourrait évoquer un diagnostic différentiel incluant un nævus en régression (halo nævus) ou, de manière plus préoccupante, un mélanome en phase de régression (mélanome regressif). Une évaluation clinique et histopathologique est nécessaire pour un diagnostic définitif.					
5. L'image dermoscopique présentée est très suggestive d'un Naevus de Sutton (Halo Naevus). Elle montre une lésion pigmentée centrale (le naevus) entourée d'un halo blanc (achromique), régulier et symétrique, correspondant à la zone de dépigmentation.<br>Structures dermoscopiques observées :					

Lésion centrale (Naevus): Elle présente une pigmentation principalement homogène ou globulaire/réticulaire (difficile à affirmer avec certitude sans un grossissement plus élevé, mais typiquement bénigne), dans des tons de brun clair à moyen.<br>Halo périphérique: Il est caractérisé par une zone blanche, structureless (sans structure), qui entoure de manière uniforme et régulière la lésion centrale. Cette blancheur est due à la régression mélanocytaire.<br>Contour: Le contour du naevus central est net, mais la transition vers le halo est abrupte, typique de ce phénomène de régression.<br>Le diagnostic possible est donc un Naevus de Sutton (Halo Naevus), une lésion bénigne caractérisée par une destruction auto-immune des mélanocytes.					
6. L'image dermoscopique montre une lésion pigmentée (nævus) entourée d'un halo blanchâtre et homogène, qui est une zone de dépigmentation. Le nævus central est caractérisé par une pigmentation de couleur brun clair à rougeâtre avec un motif principalement globulaire ou structuré en nid d'abeille (légèrement réticulaire) en périphérie, évoluant vers une zone de régression centrale blanchâtres et étoilées. Les bords de la lésion pigmentée centrale sont assez réguliers et le contour du halo est également symétrique (circulaire ou ovale). Le diagnostic possible le plus fréquent pour ce type d'image, en particulier chez un sujet jeune, est un nævus de Sutton (ou halo nævus), une lésion mélanocytaire bénigne en cours de régression immunitaire.					
1. Παρατηρείται μία καλοήθης δερματοσκοπική βλάβη, πιθανότατα ένας αχρωματικός ή υποχρωματικός σπίλος με άλω. Η βλάβη έχει κεντρική λευκωπή/υποχρωματική περιοχή με ακτινωτές ή αστεροειδείς λευκές δομές (πιθανόν ινώδης ιστός/ουλή ή παλινδρομούσα ζώνη). Γύρω από την κεντρική περιοχή υπάρχει μία περιφερική καφέ/καστανή ζώνη με ακανόνιστο, δικτυωτό ή κοκκώδες μοτίβο (πιθανόν υπολείμματα μελανίνης). Τα όρια φαίνονται ασαφή και δαντελωτά στην περιφέρεια, ενώ η συνολική βλάβη περιβάλλεται από μία λευκωπή άλω (halo), τυπική ένδειξη ανοσολογικής παλινδρόμησης (υποστροφής). Το επικρατούν χρώμα είναι το καφέ σε συνδυασμό με το λευκό/υποχρωματικό.					
2. Η δερματοσκοπική εικόνα δείχνει μια καλοήθης εμφάνισης μελαγχρωματική βλάβη με σαφή χαρακτηριστικά παλινδρόμησης. Κυριαρχεί μια μεγάλη, κεντρική περιοχή υπομελάγχρωσης/αποχρωματισμού (λευκό/υπολευκό χρώμα), η οποία περιέχει έντονες, λευκές, ακτινωτές ινώδεις δομές (λευκή ουλή/ίνωση), χαρακτηριστικές του φαινομένου της παλινδρόμησης. Η					

λευκή αυτή περιοχή περιβάλλεται από έναν ακανόνιστο, μερικώς διατηρημένο, περιφερικό δακτύλιο μελαγχρωματικού δέρματος, όπου διακρίνεται ένα ανοιχτό καφέ δικτυωτό μοτίβο. Τα όρια μεταξύ της λευκής και της καφέ ζώνης είναι ακανόνιστα. Αυτά τα ευρήματα (κεντρική παλινδρόμηση με περιφερικό μελαγχρωματικό δίκτυο) είναι ενδεικτικά ενός Σπίλου Sutton στο στάδιο της παλινδρόμησης, αν και η τελική διάγνωση απαιτεί κλινική και ιστοπαθολογική συσχέτιση					
3. Η δερματοσκοπική εικόνα απεικονίζει μια βλάβη με κεντρική λευκή ινώδη δομή που περιβάλλεται από μια περιοχή υποκυανού (γκριζο-μπλε) χρωματισμού, η οποία εξωτερικά περιβάλλεται από ένα καφέ δικτυωτό μοτίβο. Τα όρια της βλάβης εμφανίζονται ασαφή και ακτινωτά. Η παρουσία της κεντρικής λευκής ινώδους δομής (που μπορεί να υποδηλώνει ίνωση ή ουλή) και του υποκυανού χρωματισμού (που συχνά υποδηλώνει μελανίνη στο χόριο) είναι σημαντικά ευρήματα. Το πολύμορφο αυτό μοτίβο, με την ακτινωτή επέκταση και τα ασαφή όρια, καθιστά αναγκαία την περαιτέρω διερεύνηση, καθώς θα μπορούσε να αντιπροσωπεύει μια άτυπη μελαγχρωματική βλάβη, όπως ένα σπιτζοειδές νεοπλάσμα (ιδιαίτερα αμελανωτικό/υπομελανωτικό) ή άτυπο σπίλο, ή ακόμη και μελάνωμα με περιοχές παλινδρόμησης (η λευκή δομή). Η πιθανή διάγνωση απαιτεί ιστοπαθολογική εξέταση.					
4. Η εικόνα δείχνει μια μελαγχρωματική βλάβη με ασαφή όρια και ανομοιόμορφη κατανομή χρώματος. Κυριαρχούν οι καφέ και ανοιχτό καφέ (ταν) αποχρώσεις στο περιφερικό τμήμα, ενώ στο κέντρο παρατηρείται μια περιοχή λεύκανσης ή αποχρωματισμού, η οποία είναι πιο λευκή από το περιβάλλον δέρμα. Αυτό το κεντρικό λευκό τμήμα εμφανίζει και κάποια λευκά, ακτινωτά ή αστεροειδή νημάτια/δομές (μοιάζουν με λευκές ουλές ή δομές ίνωσης), καθώς και ενδεχομένως διάσπαρτα γκριζο-μπλε στίγματα/κόκκους περιφερικά του κεντρικού αποχρωματισμού. Οι δομές αυτές, δηλαδή οι λευκές ουλές και οι γκριζο-μπλε κόκκοι είναι χαρακτηριστικά σημεία παλινδρόμησης) της βλάβης. Το μοτίβο παλινδρόμησης καθιστά τη διάγνωση δύσκολη, καθώς μπορεί να εμφανιστεί σε καλοήθεις βλάβες (όπως μελαγχρωματικοί σπίλοι ή lichen planus-like keratosis) ή σε κακοήθες μελάνωμα. Ως εκ τούτου, μια βλάβη με εμφανή χαρακτηριστικά παλινδρόμησης χρήζει άμεσης περαιτέρω διερεύνησης και βιοψίας για αποκλεισμό μελανώματος.					
5. Η δερματοσκοπική εικόνα παρουσιάζει μια καλοήθους όψεως βλάβη, πιθανότατα σπίλο, με αρκετά σαφή, αν και ελαφρώς ακανόνιστα, όρια. Κυριαρχεί ένα καφέ, δικτυωτό μοτίβο στην περιφέρεια, το οποίο					

σταδιακά εξασθενεί προς το κέντρο. Το κεντρικό τμήμα της βλάβης χαρακτηρίζεται από μια υποχρωματισμένη ή λευκωπή περιοχή, η οποία περιβάλλεται από λευκές ακτινωτές ή αστεροειδείς δομές (λευκές ακτινωτές/ακτινοειδείς γραμμές), που θα μπορούσαν να αντιστοιχούν σε περιοχές ίνωσης ή σε φαινόμενο τύπου Halo Nevus (σπίλος με άλω), όπου υπάρχει μια κεντρική περιοχή παλινδρόμησης. Η συνολική συμμετρία και η ομοιογένεια των χρωμάτων (κυρίως καφέ) υποστηρίζουν τη διάγνωση ενός καλοήθους μελανοκυτταρικού σπίλου, ενδεχομένως με στοιχεία παλινδρόμησης.					
6. Η δερματοσκοπική εικόνα απεικονίζει μια βλάβη με κεντρική λευκή ινώδη δομή (λευκή ουλή ή ίνωση) και περιφερικό καφεοειδές δίκτυο (reticular pattern) που είναι ελαφρώς ακανόνιστο, υποδηλώνοντας την παρουσία μελανίνης στην επιδερμίδα. Το χρώμα κυμαίνεται από ανοιχτό καφέ στην περιφέρεια έως λευκό/υποπληρωμένο στο κέντρο, με μία ήπια ερυθρότητα γύρω από την κεντρική λευκή περιοχή, πιθανώς λόγω φλεγμονής ή αγγείωσης. Τα όρια της βλάβης είναι σχετικά σαφή αλλά όχι απολύτως ομοιόμορφα. Το σύνολο των ευρημάτων, ειδικά η κεντρική λευκή ίνωση (πιθανή υποστροφή) σε συνδυασμό με το ακανόνιστο περιφερικό μελαγχρωματικό δίκτυο, θέτει την υποψία για ένα μελαγχρωματικό μόρφωμα που έχει υποστεί μερική παλινδρόμηση. Η παρουσία τριχών εντός της βλάβης είναι επίσης ορατή.					
1. Auf diesem dermoskopischen Bild ist eine Pigmentläsion zu sehen, die in der Peripherie durch ein bräunliches, pseudo-netzartiges Muster gekennzeichnet ist, welches möglicherweise auf eine beginnende Pigmentierung im Übergang zur normalen Haut hindeutet. Im Zentrum der Läsion dominiert eine weißlich-blasse bis leicht rötliche Fläche mit einigen weißlich-sternförmigen (narbenartigen) Strukturen, die auf Regression oder eine entzündliche Komponente hindeuten. Die Grenzen der Läsion sind insgesamt unscharf und verwaschen. Das zentrale Muster der Regression zusammen mit dem peripheren Pigmentnetzwerk ist charakteristisch für einen Regressiven Halo-Nävus (Sutton-Nävus), wobei zur definitiven Diagnose eine klinische Korrelation und gegebenenfalls eine weitere Abklärung erforderlich sind.					
2. Auf diesem dermoskopischen Bild ist eine relativ gut umschriebene, ringförmig strukturierte Läsion sichtbar. Farben und Muster: Die zentrale Struktur wird von einem prominenten, weißlichen, sternförmigen oder strahlenförmigen Bereich (Fibrose/Narbengewebe)					

dominiert. Dieser ist umgeben von einer konzentrischen Zone bräunlicher Pigmentierung, welche ein feines pseudoretikuläres (netzartiges) Muster aufweist. Das Pigmentnetzwerk erscheint fein und in der Peripherie leicht verblassend.<br>Grenzen: Die äußere Begrenzung der gesamten Läsion erscheint insgesamt unscharf und leicht auslaufend ohne eine klar definierte, scharfe Kante.<br>Mögliche Interpretation: Die Kombination aus der zentralen weißlichen (narbenähnlichen) Struktur und der peripheren Pigmentierung ist ein deutliches Zeichen für eine Läsion mit Regression (Rückbildung). Solche dermoskopischen Befunde können bei einem regressiven Nävus (Muttermal) oder, was eine sofortige und sorgfältige Abklärung erfordert, bei einem regressiven Melanom beobachtet werden. Die zentrale weiße Fibrose deutet auf einen entzündlichen Prozess hin, der zur Zerstörung von Pigmentzellen geführt hat.					
3. Auf dem dermoskopischen Bild ist eine relativ symmetrische, unregelmäßige Läsion mit einer zentralen, unscharf begrenzten hypopigmentierten (weißlichen) Struktur zu erkennen, die eine sternförmige oder radiäre Ausbreitung feiner weißer Linien aufweist. Diese zentrale Region ist von einer braunen, pigmentierten Peripherie umgeben. Das periphere Pigmentmuster erscheint als ein Pseudo-Netzwerk (ein braunes, unregelmäßiges Netzwerk, das sich über der Hautstruktur abzeichnet), wobei die Pigmentierung von der Mitte nach außen hin abzunehmen scheint. Die äußere Grenze der gesamten Läsion ist unscharf und unregelmäßig. Eine mögliche Diagnose bei diesem dermoskopischen Erscheinungsbild, insbesondere mit der zentralen Hypopigmentierung, dem Pseudonetzwerk und den unscharfen Rändern, könnte ein Riesenzellnaevus (Spitz-Naevus) im regressiven Stadium oder eine andere Form der regressiven Läsion sein.					
4. Das dermoskopische Bild zeigt eine leicht erhöhte, zentripetal angeordnete Läsion mit einer Kombination von Farben. Im Zentrum befindet sich eine auffällige weißliche, sternförmige (narbenähnliche) oder milchig-weiße Struktur, die auf Regression oder Fibrose hindeuten kann. Diese zentrale Zone ist von einem bräunlichen Areal umgeben, das ein atypisches retikuläres Muster oder eine pseudoretikuläre Textur mit unregelmäßigen Maschen zeigt. Die äußere Begrenzung der Läsion erscheint unscharf und unregelmäßig. Zusätzlich sind möglicherweise feine, weiße Linien an der Peripherie erkennbar. Das Vorhandensein von weißen und bläulich-weißen Schleiern oder Strukturen, kombiniert mit dem atypischen Pigmentnetz und der Regression, lässt den Verdacht auf ein malignes					

Melanom, insbesondere einen regressiven Typ, aufkommen. Es muss jedoch eine breite Palette an Differenzialdiagnosen, einschließlich eines Basalzellkarzinoms oder anderer pigmentierter Läsionen, in Betracht gezogen werden.					
5. Auf diesem dermoskopischen Bild ist eine bräunliche Makula zu sehen, die eine reguläre Struktur aufweist. Die Farbe ist überwiegend hellbraun und zeigt ein netzartiges (retikuläres) Muster, das zur Peripherie hin ausläuft. Charakteristisch ist eine zentrale, scharf begrenzte, weiße oder hypopigmentierte (narbenartige) Fläche, aus der feine, weiße, glänzende Streifen (Shiny White Streaks/Chrysalis-Strukturen) radial nach außen strahlen, was auf eine zentrale Fibrose hinweist. Die peripheren Grenzen erscheinen relativ symmetrisch und regulär. Dieses dermoskopische Bild ist typisch für ein Dermatofibrom (gutartige fibröse Histiozytom), insbesondere aufgrund der Kombination aus peripherem Pigmentnetzwerk und zentraler weißer, narbenartiger Struktur mit den radialen weißen Streifen.					
6. Auf diesem dermoskopischen Bild ist eine relativ große, bräunliche, scharf begrenzte Makula auf der hellen umgebenden Haut sichtbar. Die Makula weist eine leichte, gleichmäßige Hyperpigmentierung auf, ähnlich einem Café-au-lait-Fleck. Im Zentrum dieser Läsion befindet sich ein helleres, weißlich-rosa Areal mit feinen, sternförmigen (radiären) oder fiederartigen Ausläufern, die in die bräunliche Umgebung ragen. In dem bräunlichen Bereich sind zudem feine, unregelmäßige netzartige Strukturen (Pigmentnetzwerk) erkennbar. Die Ränder der gesamten Läsion erscheinen scharf begrenzt, wobei die bräunliche Färbung relativ abrupt in die normale Hautfarbe übergeht. Die sichtbaren Strukturen könnten mit einem Naevus spilus (Kiebitzei-Naevus) in einem frühen oder regredienten Stadium der aufgesetzten Nävi vereinbar sein, wobei die zentrale Aufhellung eine Fibrose oder Regression innerhalb der pigmentierten Läsion andeuten könnte.					

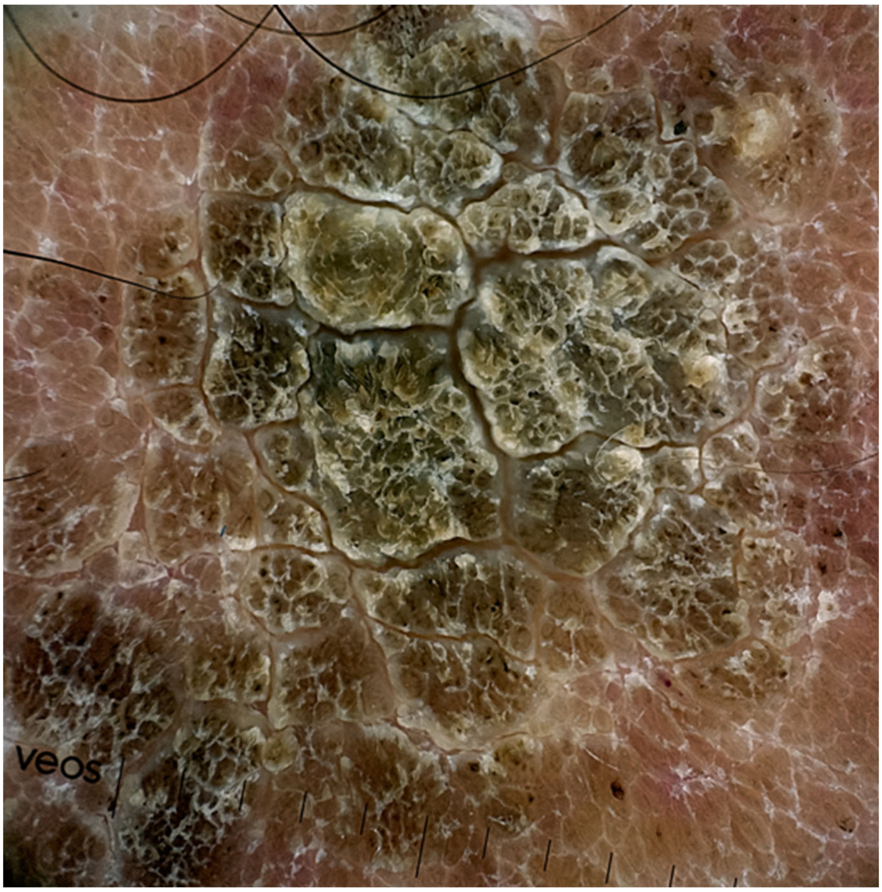

Description	Completeness	Lack of false information (accuracy)	Evidence-based content	Appropriateness	Relevance
1. This dermoscopic image displays a large, exophytic lesion characterized by a highly irregular, cerebriform surface pattern composed of numerous, interconnecting gyri and sulci, giving it a verrucous or 'brain-like' appearance. The predominant colors are brown (varying from light to dark) and a significant component of yellow-green/grey-green crusting or keratin centrally. The surrounding skin shows a slightly erythematous and perhaps dry or scaly appearance. The lesion exhibits a sharp, well-defined border (suggesting benignity) that gradually fades into the surrounding skin at some points. The overall structure and colors are highly suggestive of a large Seborrheic Keratosis (SK), especially the typical "fat fingers" or cerebriform pattern, often accompanied by scale or keratin buildup					
2. This dermoscopic image displays a sharply demarcated and highly textured lesion characterized by a distinct cerebriform (gyri and sulci) or cobblestone pattern. The colors primarily range from light to dark brown, interspersed with areas of					

yellowish-gray scale or crusting on the surface. Within this lobulated structure, key dermoscopic features, including numerous milia-like cysts (white/yellow globular structures) and comedo-like openings (dark, pseudofollicular openings), are prominently visible. This combination of patterns and structures is classically diagnostic of a Seborrheic Keratosis.					
3. Based on the dermoscopic image, the lesion exhibits a highly textured, verrucous surface with a characteristic cerebriform (gyriform) pattern composed of interconnected ridges and sulci. The colors are variable, showing shades of brown and tan with notable central greenish-grey or dark brown areas, likely representing scale, keratin, or crusting. Key dermoscopic structures observed include numerous dark, punctate comedo-like openings and scattered small, white/yellowish milial-like cysts. The overall border appears relatively well-demarcated but with a somewhat irregular or moth-eaten periphery. This specific combination of features is overwhelmingly diagnostic of a Seborrheic Keratosis					
4. This dermoscopic image displays a predominantly brown-to-light-tan lesion with a sharply demarcated, lobulated border and an overall highly textured, warty surface. The most prominent dermoscopic structures are numerous fissures and ridges forming a cerebriform pattern (brain-like appearance) throughout the lesion, which are characteristic of a papillomatous surface. Scattered yellowish-white structures likely represent milia-like cysts and light brown/greenish keratin is visible within the depressed areas and fissures. These features—sharp border, cerebriform pattern, fissures/ridges, and milia-like cysts—are classic dermoscopic findings for a Seborrheic Keratosis.					
5. The image displays a polypoidal, cerebriform, or cobblestone-like surface pattern characterized by distinct, rounded or polygonal lobules separated by deep, pale fissures/clefts, which gives it a gyrate or brain-like appearance. The overall color is a yellowish-brown to dark brown base with yellowish-white to greenish-grey keratotic material or crusting (scale-crust) particularly in the center and on the tops of the lobules. Scattered white or light blue/grey structures (possibly milia-like cysts or horn pseudocysts) may also be present, although the dense crusting obscures some detail. The border appears sharply demarcated and relatively regular. This combination of features—cerebriform/cobblestone pattern, yellowish-brown color, sharp border, and presence of keratotic material—is highly suggestive of a Seborrheic					

Keratosis, particularly an irritated or hyperkeratotic variant.					
6. The image displays a well-demarcated, polygonal or cobblestone-like pattern characterized by prominent, deeply convoluted fissures separating lobules or gyri. The dominant colors are various shades of light to dark brown, often with a yellowish-white/grayish-green scale or crusting visible on the surface, particularly in the center. The overall pattern is highly suggestive of a Seborrheic Keratosis, a common, benign epidermal tumor, with the cerebriform (brain-like) surface being a classic feature. Other features that might be present in Seborrheic Keratoses, though less distinct here, could include comedo-like openings (milia-like cysts) or hairpin vessels.					
1. Cette image dermoscopique révèle une lésion cutanée caractérisée par une coloration dominante brun-jaunâtre et vert-grisâtre, présentant une structure de surface extrêmement irrégulière et verruqueuse (papillomateuse). Le motif principal est cérébriforme ou en forme de circonvolution, créé par des crêtes épidermiques (épaississements) séparées par des sillons pigmentés. On observe la présence de multiples ouvertures pseudo-comédoniennes et de pseudo-kystes cornés (structures rondes ou ovalaires de couleur blanche/jaunâtre), qui sont des éléments dermoscopiques classiques et très évocateurs d'un diagnostic bénin. L'aspect général (couleur, relief, pseudo-kystes/comédons, et patron cérébriforme) est fortement suggestif d'une Kératose Séborrhéique (verrue séborrhéique).					
2. L'image dermoscopique révèle une lésion cutanée présentant un motif pavimenteux ou polylobulé bien délimité, communément appelé structure cérébriforme ou en gyrus. La lésion est composée de structures claires, souvent appelées pseudo-kystes cornés (ou "milia-like cysts"), et d'une pigmentation hétérogène allant du brun clair au brun foncé, avec des zones verdâtres/grisâtres, souvent interprétées comme des ouvertures folliculaires pigmentées ou des dépôts de kératine et de mélanine. La présence de ces caractéristiques (structure cérébriforme, pseudo-kystes cornés) est très suggestive du diagnostic de Kératose Séborrhéique. La démarcation nette des bords contribue également à orienter vers une lésion bénigne.					
3. Cette image dermoscopique révèle une lésion cutanée caractérisée par un motif papillaire-réniforme (ou en pavés) proéminent, typique de l'hyperplasie					

épidermique. Les pavés centraux présentent une coloration hétérogène allant du jaunâtre-vert au brun-gris foncé, avec des zones squameuses et des crevasses profondes. Les contours de la lésion apparaissent lobulés ou dentelés. Ces caractéristiques dermoscopiques, notamment le motif en pavés, la pigmentation irrégulière et la desquamation/kératose, sont fortement évocatrices d'une Kératose Séborrhéique de type hyperkératosique et pigmentée. D'autres diagnostics pourraient inclure le carcinome basocellulaire ou l'épithéliome spinocellulaire, mais le motif général est très suggestif d'une lésion bénigne.					
4. Sur cette image dermoscopique, on observe une lésion cutanée qui semble être une Kératose Séborrhéique (verrue séborrhéique) en raison de la présence de plusieurs structures caractéristiques. Les couleurs dominantes sont le brun clair au brun-vert/gris-vert central, le tout sur un fond érythémateux-rosé de peau. La lésion présente un contour bien défini mais irrégulier ou « mité ». Les motifs structurels clés incluent de multiples pseudo-kystes cornés (structures rondes, blanches à jaunâtres) et des ouvertures pseudo-comédoniennes (trous ovales, brun-noir), ainsi qu'un patron dit « cérébriforme » (crêtes et sillons ressemblant à la surface du cerveau) dans la zone plus pigmentée et verruqueuse. Ces éléments sont fortement évocateurs d'une prolifération épidermique bénigne.					
5. Cette image dermoscopique révèle une lésion pigmentée et hautement verruqueuse ou kératosique avec un aspect lobulé distinct, souvent décrit comme cérébriforme (ressemblant aux circonvolutions du cerveau). Les couleurs dominantes sont le brun foncé au marron clair, avec des zones centrales ou en surface de teinte jaunâtre et verdâtre (probablement de la kératine et des croûtes). On observe des fissures profondes ou des sillons qui séparent les lobules surélevés (crêtes). La présence de structures en forme de trous (pores élargis ou ouvertures folliculaires) et de ce qui pourrait être des kystes de milium (petites structures blanches-jaunâtres) renforce ce diagnostic. Les contours semblent assez bien définis, mais le bord de la lésion apparaît irrégulier, avec une démarcation nette par rapport à la peau saine adjacente.<br>Diagnostic possible : L'ensemble de ces caractéristiques (structures cérébriformes, fentes/sillons, trous, possibles kystes de milium, aspect kératosique) est fortement évocateur d'une Kératose Séborrhéique, une lésion cutanée bénigne très courante.					

6. Cette image dermoscopique montre une lésion papuleuse ou en plaque, caractérisée par un motif "en pavé" ou polygonal, avec des structures lobulées bien délimitées, séparées par des sillons. Les couleurs observées sont hétérogènes, allant du brun clair/chair au brun foncé, avec une composante importante de couleur vert-grisâtre ou jaune-vert au centre des structures. Cette combinaison de couleurs et ce motif de surface très structuré et squameux sont fortement évocateurs d'une Kératose Séborrhéique Irritée ou Pigmentée ou d'une lésion d'aspect verruqueux. La présence de la couleur vert-grisâtre, en particulier, peut parfois suggérer la présence d'une kératine ou de croûtes/squames spécifiques, mais dans ce contexte général, le motif en pavé est la clé du diagnostic bénin le plus probable.					
1. Η δερματοσκοπική εικόνα παρουσιάζει μια καλά αφοριζόμενη, πολυλοβωτή βλάβη με ποικιλία χρωμάτων, κυρίως καφέ, κίτρινου-γκρι (σαν λιπαρός φλοιός) και μαύρου. Η επιφάνεια έχει ένα χαρακτηριστικό ψευδοδικτυωτό μοτίβο που σχηματίζεται από ευρείες, λευκές γραμμές που περιβάλλουν μικρές, καφέ ή κιτρινωπές νησίδες, δίνοντας μια όψη που συχνά περιγράφεται ως "εγκεφαλοειδής" ή "ρυτιδωτή". Τα όρια είναι έντονα και φαίνεται να έχουν ένα χαρακτηριστικό "ψευδοθύλακα" ή "κερατοειδές θυλάκιο" γύρω από τις δομές. Η γενική εμφάνιση αυτών των δομών, συμπεριλαμβανομένης της παρουσίας κυστεοειδών δομών γεμάτες κερατίνη και του ακανόνιστου, λιπαρού φλοιού, είναι ιδιαίτερα ενδεικτική για Σμηγματορροϊκή Κεράτωση					
2. Στη δερματοσκοπική εικόνα παρατηρείται μια καλά αποσαφηνισμένη βλάβη με λοβώδη όρια και πολυχρωμία, με κυρίαρχους τους καφέ, κιτρινωπούς και πρασινωπούς-γκρι τόνους. Το κυρίαρχο μοτίβο είναι εγκεφαλοειδές ή πολυλοβωτό, που σχηματίζεται από ένα δίκτυο γραμμικών αυλακώσεων και κορυφών, που ορίζουν μικρότερους κύκλους ή οζίδια. Επίσης, είναι εμφανείς δομές όπως λευκοκίτρινες κύστεις που μοιάζουν με μίλια και ανοίγματα που μοιάζουν με φαγέσωρες χαρακτηριστικές δομές που υποδηλώνουν σμηγματορροϊκή κεράτωση μια συχνή, καλοήθης επιδερμική βλάβη.					
3. Η παρεχόμενη δερματοσκοπική εικόνα παρουσιάζει μια καλά περιγεγραμμένη βλάβη με ένα χαρακτηριστικό πολυλοβωτό ή εγκεφαλοειδές επιφανειακό μοτίβο, συχνά περιγραφόμενο ως "πέτρινες πλάκες" (cobbly or paving-stone appearance). Τα χρώματα που κυριαρχούν είναι το					

καφέ σε διάφορες αποχρώσεις, από ανοιχτό έως σκούρο, με ενδιάμεσες περιοχές που φαίνονται κιτρινωπές ή πρασινοκίτρινες λόγω της παρουσίας κερατίνης ή σμήγματος. Παρατηρούνται ψευδοκύστες κερατίνης και ψευδο-θυλακικά ανοίγματα , δομές που είναι ιδιαίτερα ενδεικτικές. Τα όρια είναι σαφή και απότομα. Αυτά τα δερματοσκοπικά χαρακτηριστικά είναι άκρως τυπικά για μια Σμηγματορροϊκή Υπερκεράτωση .					
4. Στη δερματοσκοπική αυτή εικόνα παρατηρείται μια καλά αφοριζόμενη, πολυλοβωτή βλάβη με ασύμμετρη αρχιτεκτονική. Το κυρίαρχο μοτίβο είναι το μοτίβο εγκεφαλικής βοστρύχους, χαρακτηριζόμενο από στρογγυλές ή ωοειδείς, ακανόνιστες, δίκτυο-όμοιες δομές, οι οποίες περιβάλλονται από θηλώδεις ή βοστρυχώδεις προεκβολές. Τα χρώματα ποικίλλουν και περιλαμβάνουν αποχρώσεις του καφέ, του κιτρινωπού/ωχρού και κάποιες πράσινες ή γκριζοπράσινες περιοχές (πιθανώς λόγω εγκλωβισμένου σμήγματος ή δευτερογενούς λοίμωξης/ρύπανσης), καθώς και ρόδινες ή ερυθηματώδεις περιοχές στο περιβάλλον δέρμα. Τα όρια της βλάβης φαίνονται σαφή και ομαλά, αλλά εμφανίζουν πολυλοβωτή ή ακανόνιστη μορφή λόγω του μοτίβου ανάπτυξης. Αυτά τα χαρακτηριστικά (πολυλοβωτή όψη, ψευδο-κύστες κερατίνης, μοτίβο εγκεφαλικής βοστρύχους, ποικιλία χρωμάτων) είναι ισχυρά ενδεικτικά μιας Σμηγματορροϊκής Κεράτωσης.					
5. Η δερματοσκοπική εικόνα παρουσιάζει μία καλά περιγεγραμμένη βλάβη με πολυλοβωτή, σαν-εγκάρσια-τομή εμφάνιση. Το κυρίαρχο μοτίβο είναι αυτό των εγκεφαλόμορφων (ελικοειδών) αυλακώσεων και βοθρίων, δίνοντας μια όψη που θυμίζει "λαχανικό". Παρατηρείται ένα ευρύ φάσμα χρωματικών αποχρώσεων, συμπεριλαμβανομένου του κίτρινου-γκρι, ανοιχτού καφέ, σκούρου καφέ και ελαφρώς πρασινωπού (ιδίως στο κέντρο), υποδεικνύοντας υπερκεράτωση και πιθανώς κηρώδη στοιχεία. Τα όρια της βλάβης είναι σαφή και απότομα, ενώ η περιφέρεια φαίνεται να αποτελείται από μικρότερες, κυκλικές ή ωοειδείς δομές (ψευδοκύστες κερατίνης/κυψέλες). Αυτή η συνολική εμφάνιση είναι πολύ χαρακτηριστική και ισχυρά ενδεικτική για Σμηγματορροϊκή Κεράτωση.					
6. Αυτή η δερματοσκοπική εικόνα απεικονίζει μια καλοήθη κεράτωση (πιθανότατα σμηγματορροϊκή κεράτωση), χαρακτηριζόμενη από ένα πολυλοβωτό (λοφώδες) μοτίβο που θυμίζει "γυράκια" ή "περιοχές εγκεφάλου" (δομές που μοιάζουν με έλικες και αύλακες). Τα όρια της βλάβης είναι σαφώς οριοθετημένα, δίνοντας την αίσθηση ότι είναι					

"κολλημένη" πάνω στο δέρμα. Τα επικρατέστερα χρώματα είναι το ανοιχτό και σκούρο καφέ, το κιτρινωπό-πρασινωπό (κερατίνη) και το υπόλευκο-ροζ (δέρμα βάσης). Παρατηρείται έντονη επιφανειακή υπερκεράτωση και ψευδοθυλάκια (ανοίγματα που μοιάζουν με θυλάκια) ή/και κυρτώσεις (κύστες που μοιάζουν με μικροκύστεις), τα οποία αποτελούν κλασικά δερματοσκοπικά σημεία αυτής της βλάβης.					
1. Auf diesem dermoskopischen Bild ist eine stark pigmentierte, deutlich erhabene Läsion zu erkennen, deren Erscheinungsbild typische Merkmale einer Seborrhoischen Keratose (Alterswarze) aufweist. Die vorherrschenden Farben reichen von verschiedenen Braun- und Gelbbrauntönen bis hin zu grau-grünlichen und fast schwarzen Arealen, wobei die Farben oft opak wirken. Ein charakteristisches Muster sind die polymorphen, oft **abgerundeten oder ovalen Pseudohornzysten (Milien-ähnliche Strukturen), die als weißlich-gelbliche, opake Flecken erscheinen. Ein weiteres auffälliges Muster ist die hirnwindungsartige oder fingerabdruckartige Oberfläche, die durch die deutliche Furchung und die netzartigen Linien entsteht. Die Grenzen der Läsion zur umgebenden, normal erscheinenden Haut sind scharf und deutlich begrenzt. In der Gesamtschau der Strukturen (multiple Pseudohornzysten, fette/opake Farben, zerklüftete/hirnwindungsartige Oberfläche und scharfe Begrenzung) ist die mögliche Diagnose am wahrscheinlichsten eine pigmentierte Seborrhoische Keratose.					
2. Auf diesem dermoskopischen Bild ist eine Läsion mit einem deutlichen zerebriformen (hirnartigen) Muster zu sehen, das durch Furchen (Fissuren) und Erhebungen (Grübchen/Rücken) gekennzeichnet ist. Die Läsion weist verschiedene Brauntöne auf, von hellbraun bis dunkelbraun, mit Bereichen von gelblich-grauer Keratose in der Mitte. Die Grenzen erscheinen an vielen Stellen scharf begrenzt und leicht ausgefranst ("moth-eaten" oder scharf demarkiert). Diese Kombination von Strukturen – zerebriformes Muster, Fissuren/Rücken, milienartige Zysten (könnten die helleren, gelblichen Strukturen sein) und scharfe Begrenzung – sind typische dermoskopische Merkmale einer seborrhoischen Keratose (auch Alterswarze genannt), einer häufigen gutartigen epidermalen Neoplasie.					
3. Auf diesem dermoskopischen Bild ist eine stark polymorphe Läsion zu erkennen, die primär durch das sogenannte zystenartige Muster und eine scharf					

begrenzte Randstruktur gekennzeichnet ist. Im Zentrum dominieren gelblich-weiße und grünlich-braune Bereiche mit einer gehirnartigen (zerebriformen) Oberfläche, die aus Furchen und Windungen besteht. Diese Strukturen sind typisch für Seborrhoische Keratosen (Alterswarzen) und entsprechen histologisch den Hornzysten und der papillomatösen Hyperplasie. Die Ränder der Läsion sind relativ abrupt und scharf abgegrenzt vom umgebenden Gewebe, was ebenfalls stark für eine benigne, epitheliale Läsion spricht. Die Mischung aus hell- und dunkelbraunen sowie gelblichen Farbtönen mit der einzigartigen zerebriformen Struktur lässt mit hoher Wahrscheinlichkeit die Diagnose einer Seborrhoischen Keratose stellen.					
4. Das dermoskopische Bild zeigt eine deutlich gelappte und unregelmäßig begrenzte Läsion mit einem auffälligen polymorphen Muster. Es sind mehrere Kompartimente sichtbar, die durch weiße, leicht erhabene oder verdickte Septen (Wände oder Fissuren) voneinander getrennt sind, was an eine hirnwindungsartige oder cobblestone-artige Oberfläche erinnert. Die Farben reichen von Hellbraun bis Dunkelbraun in der Peripherie, wobei der zentrale Bereich eine Mischung aus grünlich-gelben bis schmutzig-grauen Tönen zeigt, was auf eine Ansammlung von Keratin und/oder serösem Exsudat hinweisen könnte. Dieses Gesamtbild, insbesondere das unregelmäßige, gelappte Muster mit den deutlichen Fissuren und den zentralen Farbunterschieden (grau-grünlich-gelbliche Krusten oder Schuppen), ist sehr charakteristisch für eine verruköse Läsion wie eine Seborrhoische Keratose, insbesondere die irritierte oder hyperkeratotische Variante.					
5. Das dermoskopische Bild zeigt eine polymorphe Läsion mit einem pflastersteinartigen oder gehirnwindungsartigen Muster (gyri-like/cerebriform pattern), das aus unregelmäßig geformten, gelblich-braunen bis grünlich-grauen Follikelöffnungen (Krypten) besteht, die durch weiße bis hellbraune Septen getrennt sind. Die Ränder der Läsion erscheinen scharf und sind von einer leicht geröteten, schuppigen Haut umgeben. Die vorherrschenden Farben sind Gelb, Braun und Grünlich-Grau, was auf eine hyperkeratotische, talgige und möglicherweise entzündliche Komponente hindeutet. Dieses Gesamtbild ist stark suggestiv für eine seborrhoische Keratose, insbesondere eine dickere, hyperkeratotische oder irritierte Variante.					

6. Das dermatoskopische Bild zeigt eine polymorphe Läsion mit einem hirnwindungsartigen (gyrierten) oder gelappten Muster, das aus tiefen Furchen (Pseudo-Fissuren) besteht, die die Läsion in zahlreiche kleine, runde bis ovale lobuläre Strukturen unterteilen. Die vorherrschenden Farben sind Gelb, Hellbraun und Dunkelbraun/Schwarz, oft mit einer weiß-grünlichen oder wachsartigen Kruste/Schuppe zentral und in den Vertiefungen. Die Läsion weist eine scharfe, aber unregelmäßige Grenze zur umgebenden normalen Haut auf. Die Gesamtstruktur ist charakteristisch für eine seborrhoische Keratose, insbesondere eine stark pigmentierte oder irritierte Variante (Keratosis seborrhoica). Andere dermatoskopische Merkmale, die typischerweise bei seborrhoischer Keratose beobachtet werden, sind die gelappten oder "fingerabdruckartigen" Strukturen, die als Pseudozysten (Milien-ähnliche Zysten) und Komedo-ähnliche Öffnungen (Pseudofollikuläre Öffnungen) erscheinen können, wobei das Gelb in den zystischen Strukturen prominent ist.					

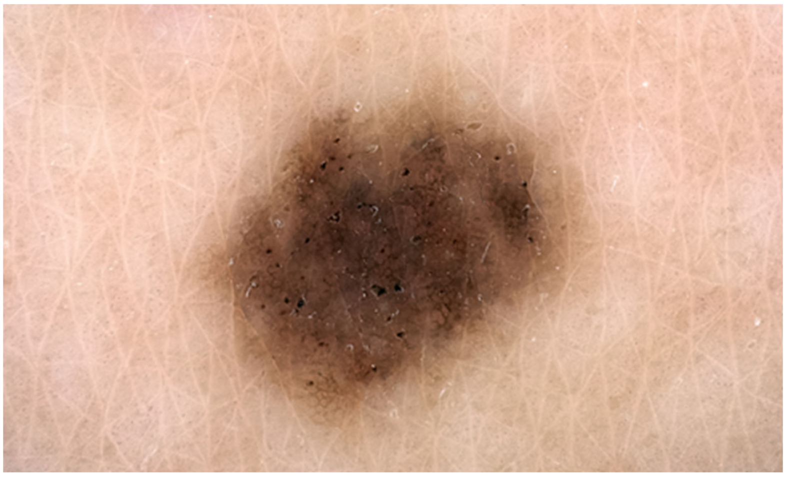

Description	Completeness	Lack of false information (accuracy)	Evidence-based content	Appropriateness	Relevance
1. The image displays a pigmented lesion observed under dermoscopy. Key dermoscopic structures include a dark brown to black color with areas of lighter brown. The overall pattern is irregular, featuring a globular or blotchy center with black dots/globules (indicating pigment aggregates in the superficial dermis or dermoepidermal junction) and reticular/pseudopodal projections extending irregularly toward the periphery. The border is ill-defined and asymmetrical. This combination of asymmetry, irregular border, multiple colors, and					

varied/atypical structures (like irregular globules and blotches) raises suspicion and is concerning for a melanoma					
2. This dermoscopic image displays a pigmented skin lesion with several concerning features. The lesion exhibits a predominantly dark brown to black color with areas of lighter brown. The pigment network is irregular and in parts, appears pseudopodal or streaming, especially toward the periphery, suggesting an atypical or malignant pattern. The overall pattern is asymmetric and the borders are poorly defined and irregular. The presence of black dots/globules and areas of homogeneous dark pigmentation further suggests a potential diagnosis of melanoma or a severely atypical nevus (dysplastic nevus). Immediate dermatological evaluation is necessary for a definitive diagnosis and treatment plan.					
3. The lesion displays significant asymmetry and an irregular border. The color is multifaceted, predominantly dark brown/black, with areas of lighter brown and possibly subtle shades of blue/grey, indicating different depths of melanin. The internal pattern appears polymorphous or atypical, lacking a classic, benign structure like a uniform reticular pattern or a central globular arrangement. The very dark areas (black dots/globules) and structureless regions suggest active pigment production and possible dermal involvement. The presence of these A, B, C, D (Asymmetry, Border irregularity, Color variation, Diameter/Dermoscopic structures) features strongly warrants further evaluation.					
4. The dermoscopic image shows an asymmetrical, darkly pigmented lesion displaying color variegation, predominantly featuring shades of dark brown and black. Key dermoscopic structures include a structureless, irregularly pigmented center with scattered black dots/globules across the surface, and an irregular, blurred border where the pigment network is largely absent or highly atypical. The combination of asymmetry, color variation, irregular borders, and structureless areas/atypical pigmentation raises suspicion for a melanocytic lesion, potentially an early melanoma (consistent with ABCDE criteria for malignancy), and requires further dermatological evaluation.					
5. Based on the dermoscopic image, I observe a lesion characterized by multiple colors, including shades of brown and darker black/blue-black specks. The pigment network appears irregular and disrupted in parts, with areas showing a globular or structureless pattern. The borders of the lesion are asymmetrical					

and ill-defined. These features—specifically the color variegation, asymmetry, irregular border, and atypical structures—are concerning findings often associated with dysplastic nevi or, more importantly, potentially melanoma.					
6. The dermoscopic image displays a pigmented skin lesion with several characteristic features. The overall color is heterogenous, predominantly a mixture of brown tones, ranging from light brown at the periphery to a darker, almost black area in the center. The pattern appears somewhat globular or irregular with a subtle tendency toward a reticular (net-like) pattern at the edges, though the central area is structureless or composed of dark dots/globules. The border is ill-defined and irregular, particularly at the bottom-left edge. This combination of asymmetry, border irregularity, color heterogeneity, and potentially atypical pigment network raises suspicion and warrants further investigation, as these are features often seen in atypical nevi or melanoma.					
1. L'image dermoscopique présente une lésion cutanée pigmentée de forme irrégulière et asymétrique. Elle est caractérisée par une hétérogénéité des couleurs, allant du brun clair au centre à des nuances de brun foncé et même quelques points noirs au centre. Le motif principal est celui d'une zone non structurée ou de globules/points foncés agrégés, avec une possible et subtile tendance à un réseau pigmentaire périphérique. Les contours sont mal définis et irréguliers. Cette combinaison de couleurs variées, de bordures atypiques et de structure non homogène est suspecte et nécessite une évaluation plus approfondie, car ces caractéristiques sont courantes dans les naevi atypiques ou les mélanomes.					
2. L'image dermoscopique présente une lésion pigmentée se caractérisant par une asymétrie de couleur et de structure. Les couleurs visibles sont principalement le brun foncé à noir avec des variations d'intensité. On observe une pigmentation diffuse et irrégulière avec la présence de points et de globules irréguliers, parfois confluents, particulièrement dans la partie centrale plus foncée. Les contours de la lésion apparaissent flous et irréguliers. En l'absence de réseau pigmentaire typique sur une grande partie de la lésion et compte tenu des structures irrégulières (asymétrie, couleurs multiples, points/globules irréguliers), ce tableau est très suspect et pourrait évoquer un diagnostic possible de mélanome malin.					
3. L'image présente une lésion pigmentée de couleur dominante brun-noir, avec une distribution de pigment					

irrégulière et des zones plus foncées (ponctuations ou globules noirs) suggérant une pigmentation en profondeur ou focale. Le contour est mal défini et irrégulier, sans symétrie claire. Le motif global pourrait être décrit comme atypique ou réticulo-globulaire irrégulier, sans la présence évidente d'un réseau pigmentaire bien formé sur l'ensemble de la lésion. Ces caractéristiques (multiples couleurs, asymétrie, bordure irrégulière, structures irrégulières) sont des critères d'inquiétude selon la règle de l'ABCDE (Asymétrie, Bordures irrégulières, Couleurs multiples, Diamètre, Évolution). Bien qu'un diagnostic précis soit impossible, l'aspect dermoscopique soulève la suspicion d'une lésion mélanocytaire atypique ou d'un mélanome, et nécessite une évaluation urgente par un dermatologue.					
4. L'image présente une lésion pigmentée dont le contour est irrégulier et mal défini. La couleur est hétérogène, allant du brun clair au brun foncé, avec des zones très sombres, voire noires, et la présence de multiples points et globules noirs/brun foncé répartis de manière irrégulière, en particulier au centre et en périphérie. Un motif réticulaire pigmenté est visible de manière incomplète et irrégulière, principalement vers les bords. La distribution des structures et de la pigmentation apparaît asymétrique. Ces caractéristiques (asymétrie, bords irréguliers, couleurs multiples, pigmentation et structures irrégulières) sont des signes qui, selon la règle mnémotechnique ABCDE de la dermoscopie (Asymétrie, Bords irréguliers, Couleur non homogène, Diamètre, Évolution), sont souvent associés à des lésions mélanocytaires suspectes ou atypiques, et soulèvent la possibilité d'un mélanome. Une évaluation et un diagnostic par un dermatologue sont nécessaires.					
5. Cette image montre une lésion pigmentée dont la couleur dominante est un brun foncé à noir avec des variations d'intensité. On observe une asymétrie de la forme et une irrégularité des bords qui semblent mal définis. Le motif dermoscopique principal est caractérisé par une agrégation de points et de globules noirs/bruns répartis de manière irrégulière, avec des zones de pigmentation diffuse. L'ensemble de ces caractéristiques, notamment l'asymétrie, l'irrégularité des bords et des couleurs, et la présence de structures pigmentaires irrégulières, est suspect et pourrait évoquer un mélanome ou un nævus dysplasique. Une évaluation clinique et histopathologique par un spécialiste est indispensable pour établir un diagnostic précis.					

6. L'image dermoscopique révèle une lésion pigmentée qui présente plusieurs caractéristiques potentiellement suspectes. La lésion est asymétrique en couleur et en structure. On observe une hétérogénéité des couleurs, allant du brun clair au brun foncé, avec des zones plus noires ou gris-noir (points noirs, fins et irréguliers, pouvant être des agrégats de mélanine ou des points/globules irréguliers). Les contours de la lésion apparaissent irréguliers et flous par endroits, et la structure générale semble atypique sans réseau réticulaire bénin classique bien défini. En raison de l'asymétrie, des bords irréguliers, et de la distribution hétérogène des couleurs et des pigments (points/globules irréguliers), cette lésion doit être considérée comme suspecte de mélanome (critères ABCDE : Asymétrie, Bords irréguliers, Couleur hétérogène) et nécessite une évaluation plus approfondie par un dermatologue.					
1. Η δερματοσκοπική εικόνα απεικονίζει μια μελαγχρωματική βλάβη με ασύμμετρη μορφή και ακανόνιστα όρια, τα οποία σε ορισμένα σημεία είναι θολά ή ψευδοποδικά. Παρατηρείται κυρίως ένα σκούρο καφέ έως μαύρο χρώμα, με πολυχρωμία και ασύμμετρη κατανομή της μελανίνης. Το κυρίαρχο μοτίβο φαίνεται να είναι άμορφο ή πολυεστιακό, με την παρουσία σκούρων κουκκίδων και σφαιριδίων διαφόρων μεγεθών, ιδιαίτερα στην περιφέρεια, καθώς και πιθανές ακανόνιστες γραμμές δικτύου. Αυτά τα χαρακτηριστικά (Ασύμμετρη μορφή, Ακανόνιστα όρια, Πολυχρωμία, και Δομική ανομοιογένεια, σύμφωνα με τον κανόνα ABCD) είναι ύποπτα για κακόηθες μελάνωμα και απαιτούν άμεση αξιολόγηση από δερματολόγο.					
2. Η παρούσα δερματοσκοπική εικόνα παρουσιάζει μία μελαγχρωματική βλάβη με ακανόνιστα όρια και ετεροχρωμία. Το κυρίαρχο χρώμα είναι το σκούρο καφέ έως μαύρο, με διακριτές διασπορές του μελανίνης (σαν "σκόνη") που υποδηλώνουν την παρουσία μαύρων κουκκίδων ή/και μικρών σφαιριδίων στο κέντρο της βλάβης. Το μοτίβο φαίνεται να είναι άμορφο ή με στοιχεία άτυπου δικτύου περιφερικά, και χαρακτηρίζεται από ασυμμετρία. Η ύπαρξη έντονης ετεροχρωμίας, ακανόνιστων ορίων, και άτυπων δομών είναι ενδεικτική για δυσπλαστικό σπίλο ή, ενδεχομένως, μελάνωμα , και χρήζει άμεσης κλινικής εκτίμησης και πιθανής βιοψίας για την τελική διάγνωση.					
3. Η δερματοσκοπική εικόνα απεικονίζει μια μελαγχρωματική βλάβη με ασύμμετρη μορφολογία και ακανόνιστα όρια που φαίνονται να σβήνουν					

σταδιακά στο περιβάλλον δέρμα. Το κυρίαρχο χρώμα είναι το σκούρο καφέ έως μαύρο, με ορισμένες περιοχές να εμφανίζουν ετεροχρωμία (ποικιλία χρωμάτων/αποχρώσεων). Ως προς τα μοτίβα, παρατηρείται ένα ασαφές, μη-δικτυωτό μοτίβο που περιλαμβάνει διάσπαρτα στίγματα/κηλίδες σκούρου χρώματος, ιδίως στην περιφέρεια και στο εσωτερικό της βλάβης, σε ένα φόντο με πιο ακανόνιστη κατανομή χρωστικής (π.χ., περιοχές μπλε-γκρι ή ομοιογενούς χρωματισμού). Το σύνολο των χαρακτηριστικών, δηλαδή η ασυμμετρία, τα ακανόνιστα όρια, η ετεροχρωμία και η παρουσία μη-συγκεκριμένου/άτυπου μοτίβου χρωστικής, είναι ενδεικτικά ενός άτυπου σπίλου ή δυνητικά ενός μελανώματος,γι' αυτό και απαιτείται άμεση περαιτέρω αξιολόγηση.					
4. Η εικόνα απεικονίζει μια μελαγχρωματική βλάβη του δέρματος με ακανόνιστα όρια και ασύμμετρη μορφή. Το χρώμα κυμαίνεται από ανοιχτό καφέ έως πολύ σκούρο καφέ/μαύρο, υποδεικνύοντας πολλαπλές αποχρώσεις (ποικιλοχρωμία). Το κυρίαρχο μοτίβο φαίνεται να είναι άμορφο (structureless) στο κέντρο, με ορισμένες ακανόνιστες γραμμώσεις ή ψευδοπόδια να προεκτείνονται προς τα όρια, αν και η εικόνα είναι κάπως θολή για οριστική ταυτοποίηση. Παρατηρούνται επίσης μαύρες κουκκίδες και κοκκία εντός της βλάβης. Η παρουσία πολλαπλών χρωμάτων, ασαφών ορίων και ασυμμετρίας την καθιστούν μια ύποπτη βλάβη που απαιτεί άμεση αξιολόγηση από δερματολόγο για τον αποκλεισμό του μελανώματος.					
5. Η παρεχόμενη δερματοσκοπική εικόνα παρουσιάζει μια καφέ-μαύρη βλάβη με ακανόνιστα όρια και ασύμμετρη μορφή. Το κυρίαρχο μοτίβο φαίνεται να είναι ετερογενές, με την παρουσία τόσο ενός αποκλειστικού, πιο σκούρου κεντρικού τμήματος όσο και λεπτών γραμμών (πιθανώς δικτυωτό ή ψευδοπόδια/ακτίνες στα όρια) που ξεθωριάζουν σταδιακά στο περιβάλλον δέρμα. Παρατηρούνται επίσης μικροσκοπικές, μαύρες κουκκίδες (στίγματα) εντός της βλάβης, υποδηλώνοντας την παρουσία μελανίνης σε ανώτερες επιστρώσεις του δέρματος. Αυτά τα χαρακτηριστικά—η ασυμμετρία, τα ακανόνιστα όρια, το πολυχρωματικό/ετερογενές χρώμα και οι διαφορετικές δομές—ενεργοποιούν το ABCDE κριτήριο (Ασυμμετρία, Ακανόνιστα όρια, Χρώμα, Διάμετρος/Εξέλιξη) και είναι ύποπτα για μελάνωμα. Ως εκ τούτου, η βλάβη αυτή απαιτεί άμεση βιοψία και ιστοπαθολογική εξέταση για την τελική διάγνωση.					
6. Αυτή η δερματοσκοπική εικόνα παρουσιάζει μια μελαγχρωματική βλάβη με αρκετά χαρακτηριστικά:					

Χρώματα: Παρατηρείται ένα κυρίαρχο σκούρο καφέ έως μαύρο χρώμα, το οποίο είναι ακανόνιστα κατανεμημένο.<br>Μοτίβα/Δομές: Το κυρίαρχο μοτίβο είναι ένα άμορφο, κεντρικά τοποθετημένο, σκούρο καφέ/μαύρο χρώμα, που μοιάζει με κυανό πέπλο ή συσσωματωμένες κουκκίδες και κηλίδες στο κέντρο. Τα όρια της βλάβης περιφερικά φαίνονται να έχουν μια ακανόνιστη δικτυωτή δομή η οποία είναι ετερογενής και ασαφής σε ορισμένα σημεία.<br>Όρια: Τα όρια είναι ασύμμετρα και ακανόνιστα σε σχέση με το περιβάλλον δέρμα.<br><br>Πιθανή Διάγνωση: Η παρουσία ακανόνιστων χρωμάτων και δομών, η ασυμμετρία, τα ακανόνιστα όρια, και ιδιαίτερα η πιθανή παρουσία κυανού πέπλου (αν και απαιτείται περαιτέρω αξιολόγηση) εγείρουν ισχυρή υποψία για κακόηθες μελάνωμα ή ένα άτυπο σπίλο (δυσπλαστικό). Η περαιτέρω κλινικο-δερματοσκοπική αξιολόγηση και πιθανή βιοψία είναι απαραίτητες για την οριστική διάγνωση.					
1. Auf dem dermoskopischen Bild ist eine asymmetrische, pigmentierte Läsion dargestellt. Die Farbgebung ist heterogen, sie reicht von dunkelbraun bis fast schwarz im Zentrum und geht in hellere Brauntöne am Rand über, was dem Kriterium "Color" (Farbe) der ABCDE-Regel entspricht. Das sichtbare dermoskopische Muster ist gestört und atypisch: Es fehlt ein regelmäßiges Pigmentnetzwerk; stattdessen dominieren strukturlos wirkende dunkle Areale gemischt mit unregelmäßigen, feinen Punkten und Kügelchen. Die Begrenzung der Läsion zur umliegenden Haut ist unscharf, verwaschen und unregelmäßig, was zusammen mit der Asymmetrie (A-Kriterium) und der unterschiedlichen Farbe (C-Kriterium) dringende Warnsignale für eine melanozytäre Neoplasie darstellt. Diese Befunde sind hochgradig verdächtig auf ein malignes Melanom und erfordern eine sofortige dermatologische Abklärung und potenziell eine histologische Untersuchung.					
2.<br>Auf diesem dermoskopischen Bild ist eine Läsion mit unregelmäßiger Pigmentierung zu sehen, die verschiedene Brauntöne, von hell bis sehr dunkel, aufweist. Die Grenzen der Läsion sind unscharf und unregelmäßig, besonders an den Rändern, wo die Pigmentierung in die umgebende Haut ausläuft. Das vorherrschende Muster ist ein atypisches Netzmuster oder ein unstrukturiertes Areal, wobei in der Mitte einige dunkle, punktartige oder kleine klumpige					

Pigmentierungen (ähnlich Punkten und Globuli) erkennbar sind, die unregelmäßig verteilt sind. Das allgemeine Erscheinungsbild, insbesondere die unregelmäßige Form, die ungleichmäßige Pigmentierung und die unscharfen Ränder, erfüllt mehrere der ABCDE-Kriterien und ist verdächtig auf ein malignes Melanom. Zur Sicherung der Diagnose ist eine ärztliche Untersuchung und gegebenenfalls eine Biopsie erforderlich.					
3. Das dermoskopische Bild zeigt eine pigmentierte Läsion mit Asymmetrie in Form und Farbe sowie unscharfen und unregelmäßigen Grenzen. Die dominierende Farbe ist ein dunkles Braun bis Schwarz, wobei die Pigmentierung heterogen erscheint und verschiedene Farbtöne sowie eine Verteilung von braun-schwarzem Pigment (wahrscheinlich als Netzwerk oder ungeordnetes Pigment mit möglicherweise einigen schwarzen Punkten/Globuli) innerhalb der Läsion aufweist. Die Läsion ist insgesamt polychromatisch (mehrfarbig). Die Beobachtung von Unregelmäßigkeit in Form, Farbe, und Begrenzung legt eine mögliche Diagnose in Richtung einer melanozytären Neoplasie nahe, wobei insbesondere die Kriterien für ein Melanom (ABCDE-Kriterien: Asymmetrie, Begrenzung unregelmäßig, Color [Farbe] heterogen, Diameter [Größe, auch wenn diese im Bild nicht abschätzbar ist], Evolution [Entwicklung]) erfüllt sein könnten. Eine umfassende klinische Korrelation und eine Biopsie wären zur endgültigen Diagnose dringend erforderlich.					
4. Auf dem dermoskopischen Bild ist eine atypische melanozytäre Läsion mit folgenden Merkmalen erkennbar: Die Farben sind inhomogen und reichen von hellem Braun bis zu sehr dunklen, fast schwarzen Arealen in der Mitte, sowie einigen sehr feinen schwarzen Punkten und/oder Globuli am Rand. Das Muster ist überwiegend strukturlos in der Mitte (braunes strukturloses Areal), mit einer undeutlichen Ausprägung eines Pigmentnetzwerks, das zum Rand hin diffus und unregelmäßig wird. Die Grenzen sind unscharf und unregelmäßig. Die Kombination aus Asymmetrie (in der Pigmentierung und Form), inhomogener Färbung und einem zentralen strukturlosen Areal mit schwarzen Punkten in der Peripherie sind alarmierende Zeichen und stark verdächtig für ein malignes Melanom.					
5. Auf diesem dermatoskopischen Bild ist eine Pigmentläsion zu sehen, die Auffälligkeiten aufweist. Die Läsion ist unsymmetrisch und die Grenzen sind in Teilen unscharf oder unregelmäßig. Die vorherrschende Farbe ist dunkelbraun bis schwarz,					

wobei verschiedene Brauntöne und eine gewisse Farbvariabilität innerhalb der Läsion erkennbar sind. Das vorherrschende Muster scheint ein atypisches Netzmuster oder ein homogenes Areal mit Pfefferkorn-ähnlichen Strukturen (feine, dunkle Punkte/Kügelchen) zu sein, insbesondere in den dunkleren Zentralbereichen. Diese Merkmale – Asymmetrie, unregelmäßige Grenzen, Farbvariabilität und atypische Strukturen – erfüllen Kriterien, die eine atypische oder dysplastische Nävuszelle oder ein Melanom vermuten lassen. Eine umgehende klinische und histopathologische Abklärung durch einen Facharzt ist dringend erforderlich.					
6. Das dermoskopische Bild zeigt eine unregelmäßig geformte, pigmentierte Läsion mit einer Mischung aus dunkelbraunen und schwarzen Farben. Die Grenzen erscheinen an vielen Stellen unscharf und unregelmäßig, was als Unregelmäßigkeit der Ränder interpretiert werden kann. Als dominierendes dermoskopisches Muster sind diffus verteilte, feine, punktartige und globuläre Strukturen im Zentrum zu erkennen, die in ihrer Dichte variieren. In den äußeren Bereichen ist eine unschärfere, netzartige oder pseudo-netzartige Struktur sichtbar, die möglicherweise an einigen Stellen unterbrochen ist oder in feinen radialen Linien (Pseudopodien) ausläuft, obwohl dies nicht eindeutig ist. Die Kombination dieser Merkmale – insbesondere die Unregelmäßigkeit von Farbe und Rand sowie die Anwesenheit von multiplem oder polymorphem Pigment – weckt den Verdacht auf eine melanozytäre Läsion wie ein dysplastischer Nävus oder, aufgrund der starken Pigmentierung und Unregelmäßigkeit, die Möglichkeit eines Melanoms. Eine definitive Diagnose und weitere Schritte erfordern eine histopathologische Untersuchung nach Exzision.					

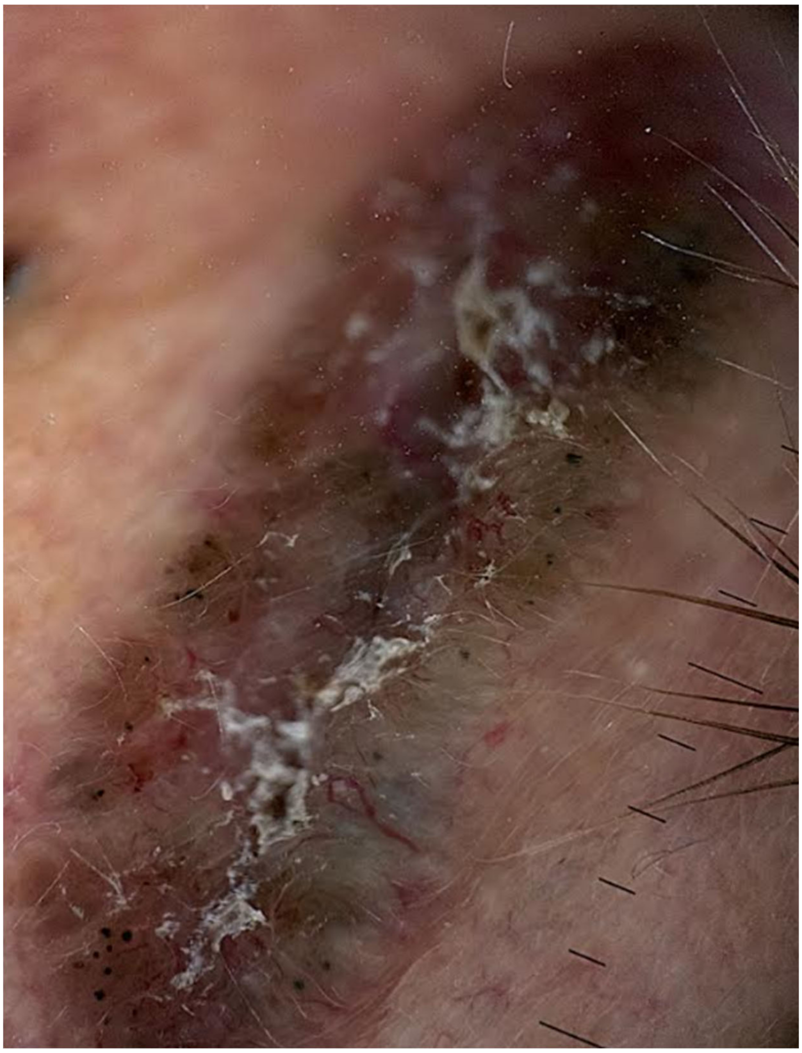

Description	Completeness	Lack of false information (accuracy)	Evidence-based content	Appropriateness	Relevance
1. This dermoscopic image displays a linear scar-like structure with surrounding areas of hyperpigmentation (dark brown/black). Within the central linear area, there is evidence of whitish-gray streaks and scale/crust which may represent fibrosis or surface hyperkeratosis/scaling. Several fine, tortuous red vessels (telangiectasias) are also visible running along and adjacent to the main lesion. The borders of the lesion appear somewhat irregular and ill-defined. Given the findings of a scar-like/atrophic appearance, linear configuration, and peripheral scaling/pigmentation, a possible differential diagnosis includes a healed or active lichenoid dermatosis, such as Lichen Planus (especially a hypertrophic or atrophic variant), or potentially a scar undergoing post-inflammatory changes, though a					

definitive diagnosis cannot be made from this image alone.					
2. The image reveals an elongated, poorly defined lesion characterized by a mix of colors including dark brown/black pigmentation, areas of erythema (redness), and significant patches of white-to-grayish scale/crust centrally. The lesion follows a linear or scar-like configuration. Visible structures include fine, irregular telangiectasias (red lines/vessels) suggesting vascular compromise or inflammation, and a few scattered dark dots/globules at the periphery. The borders are focally ill-defined and irregular. Given the scar-like appearance, central scaling, and mixed pigmentation/inflammation, this may represent an inflammatory dermatosis overlying a scar, or potentially a recurrent/residual skin cancer (like a Basal Cell Carcinoma or Squamous Cell Carcinoma) with inflammation and scarring. The presence of surrounding terminal hairs confirms this is a hairy area of skin.					
3.<br>The dermoscopic image shows a lesion characterized by a central whitish-yellow structureless area suggesting significant keratinization or scar tissue, which is typical for tumors arising on a scar, like a Marjolin's ulcer (squamous cell carcinoma on a scar). The borders are ill-defined and irregular, blending into the surrounding skin. Throughout the lesion, a prominent polymorphous vascular pattern is visible, including linear irregular vessels and hairpin vessels which are characteristic findings for invasive squamous cell carcinoma (SCC). The presence of these aggressive vascular structures and the white-to-yellow keratin mass, without typical features of basal cell carcinoma or melanoma, strongly supports a diagnosis of invasive SCC.					
4. The image displays a lesion or scar exhibiting features suggestive of a healing or inflammatory process. Notable colors include a dark brown/black pigmentation along the center, suggesting post-inflammatory hyperpigmentation or residual pigment, and areas of redness/erythema potentially indicating inflammation or vascularity. The					

most striking pattern is the presence of white, amorphous material/crust covering and lining the central structure, which might represent scale, crust, or fibrosis/scar tissue. The borders of the central area appear somewhat irregular and ill-defined, transitioning into the surrounding skin which also shows sparse, dark terminal hairs. The combination of hyperpigmentation, erythema, and white crusting/scaling on a scarred or damaged area raises the possibility of a non-specific inflammatory dermatosis, a complicated healing scar, or an infection, but no definitive features of melanoma or other specific skin cancers are clearly visible; however, a proper diagnosis requires clinical correlation and, often, a biopsy.					
5. Based on the dermoscopic image, the lesion appears to be a longitudinal scar or surgical incision site with features suggestive of healing and possible superficial infection or inflammation. Key visible structures include:<br>Color: A central dark, likely post-inflammatory hyperpigmentation (black/brown) or healing tissue, flanked by areas of erythema (redness) suggesting inflammation or vascularity. There is also prominent white, scaly material or crusting along the length of the lesion.<br>Pattern/Structures: The overall pattern is linear, following the expected contour of a healed incision. There is a fibrillar/reticular whitish pattern overlying the dark center, which could be a crust, scar tissue maturation, or fungal elements. Thin, irregular, telangiectatic vessels (red linear structures) are visible, indicative of reactive inflammation or wound healing.<br>Borders: The lesion has ill-defined borders blending into the surrounding skin, with hair follicles visible at the edges.<br>Possible Diagnosis/Interpretation: Given the linear shape and background, the lesion is a scar. The white scaling, crusting, and underlying erythema are highly suggestive of a superimposed condition, such as Tinea Incognito (if topical steroids were used), Dermatophytosis, or a contact/irritant dermatitis along the scar line. It is unlikely to					

be a primary skin cancer due to the linear, post-traumatic appearance.					
6. Based on the dermoscopic image, I observe a linear, violaceous to brownish-black lesion with a fibrotic or scarred appearance, suggesting it may be a scar, potentially post-inflammatory or post-traumatic. Within the lesion, there are areas of whitish-yellow structureless material or scale, which could indicate superimposed inflammation, infection (e.g., fungal), or a residual topical application. Fine, short, light-brown to black hairs (vellus hairs) are visible, and the lesion appears to traverse an area of denser hair, possibly the eyebrow or scalp edge. The borders are somewhat irregular and ill-defined. Given the linear, possibly scarred nature, the pattern is non-specific for typical melanocytic lesions, but the violaceous/brownish hue and irregular features warrant professional assessment to rule out any underlying skin pathology, although a scar with secondary changes seems the most likely simple explanation.					
1. L'image présente une structure cutanée linéaire, suggérant une cicatrice ou une zone d'alopécie cicatricielle. La caractéristique principale est une large zone blanche et fibreuse (fibrose), typique des cicatrices matures ou de la destruction folliculaire. Au sein et autour de cette zone blanche, on observe une pigmentation irrégulière (couleur marron/noire) sous forme de points et de globules noirs et marron, qui pourraient être des résidus de mélanine ou des signes d'inflammation/activité mélanocytaire. On remarque également la présence de duvets et de poils terminaux résiduels ou en repousse, indiquant que la destruction n'est pas complète ou qu'il y a une tentative de repousse le long des bords. Enfin, des lignes et des vaisseaux rouge-violacé très fins (télangiectasies ou vaisseaux linéaires) sont visibles, suggérant une composante vasculaire ou inflammatoire persistante ou récente. L'ensemble des motifs (large zone blanche, pigmentation irrégulière, présence de follicules et de vaisseaux) est atypique et pourrait correspondre à une cicatrice, ou					

potentiellement à une lésion cutanée (comme un mélanome développé sur une cicatrice ou un carcinome basocellulaire sclérodermiforme) dans une zone cicatricielle ou alopécique, nécessitant une évaluation clinique et histologique pour un diagnostic précis.					
2. 'observe une image dermoscopique qui semble montrer une cicatrice ou une lésion cutanée en régression/inflammation, peut-être au niveau du sourcil (à cause de la présence de poils terminaux coupés ou cassés sur la droite).<br>Cette image met en évidence une structure centrale hypopigmentée et cicatricielle (zone blanche-rosée et légèrement atrophique) qui traverse une zone cutanée. On note la présence de voile blanc-bleuté ou blanc-grisâtre et de kératine en surface (les amas blancs irréguliers), suggérant soit une hyperkératose, soit des résidus de traitement topique, ou une inflammation/infection superficielle. La lésion est bordée de pigmentation gris-brun et noire (petits points et globules) qui pourrait indiquer de l'hémosidérine post-inflammatoire, des mélanocytes ou des résidus de croûtes/saignements. Des vaisseaux sanguins télangiectasiques (lignes rouges fines) sont visibles, confirmant une activité inflammatoire ou une cicatrice récente/pathologique. La présence de ces structures cicatricielles et pigmentées, ainsi que le voile blanc, peut être associée à diverses conditions, notamment un carcinome basocellulaire sclérodermiforme/en régression, une cicatrice pathologique (chéloïde/hypertrophique), une dermite inflammatoire ou une lésion pigmentée en régression/post-traitement.					
3. L'image dermoscopique montre une lésion cutanée qui semble être une zone de cicatrisation ou de fibrose (fond blafard, blanchissant/rose) avec des signes d'inflammation et de desquamation/croûtage sur une partie de l'arcade sourcilière ou une zone pileuse.<br>Les structures observées sont :<br>Couleurs et Fond : Prédominance d'une couleur blanchâtre, nacrée et					

rosée/érythémateuse, typique du tissu cicatriciel ou scléreux/fibreux.<br>Structures de Surface : Présence de croûtes épaisses, blanchâtres à jaunâtres et filamenteuses au centre de la zone, suggérant un exsudat séreux séché ou une hyperkératose importante et de l'inflammation. On distingue également de petits points noirs et bruns qui pourraient être des croûtes hémorragiques ou des dépôts de mélanine dans une lésion sous-jacente ou une réaction post-inflammatoire.<br>Vaisseaux : Quelques vaisseaux linéaires et ramifiés (arborisations vasculaires) très fins sont visibles, ainsi que des zones de rougeur diffuse (érythème).<br>Pilosité : Des poils sont présents en périphérie et traversent la zone, indiquant que la lésion est dans une zone pileuse, mais l'aspect cicatriciel central suggère une perte pileuse ou une alopécie cicatricielle localisée.<br>Le diagnostic possible est orienté vers une lésion cicatricielle pathologique (par exemple, une cicatrice hypertrophique ou une forme cicatricielle de carcinome basocellulaire sclérodermiforme ou une dermatose inflammatoire chronique de l'unité pilosébacée comme une dermatose pustuleuse érosive du cuir chevelu - si l'emplacement est le scalp - ou une autre cause d'ulcération/croûtage dans une zone cicatricielle ou atrophique). Cependant, la présence de structures vasculaires et de croûtes dans un contexte cicatriciel nécessite toujours une évaluation clinique et histologique pour exclure une néoplasie cutanée.					
4. L'image dermoscopique montre une lésion cutanée qui semble être une cicatrice ou une zone de peau altérée, possiblement au niveau du sourcil ou d'une autre zone pileuse, comme en témoignent les poils résiduels sur la droite. La structure principale est une zone centrale hypopigmentée et atrophique, typique d'une cicatrice, qui est traversée par des fines télangiectasies (vaisseaux fins et linéaires, de couleur rouge). Sur cette cicatrice, on observe un dépôt blanchâtre et squameux irrégulier, suggérant potentiellement une infection fongique (comme une candidose ou une					

dermatophytie) ou une kératose/desquamation sur la surface cicatricielle. La couleur de fond est principalement brun-noirâtre irrégulière autour de la zone centrale claire, avec des points noirs isolés qui pourraient être des résidus de poils ou des amas de pigment. Les contours sont globalement irréguliers et flous. Un diagnostic dermoscopique direct d'un mélanome ou d'un carcinome n'est pas évident d'après les motifs classiques seuls ; les caractéristiques sont davantage compatibles avec une cicatrice enflammée/infectée ou une lésion récidivante si elle est post-opératoire. Une biopsie serait essentielle pour exclure toute récidive tumorale si la lésion initiale était maligne.					
5. L'image présente une structure cutanée linéaire, suggérant probablement une cicatrice ou une lésion très allongée. La caractéristique la plus frappante est la présence de matériel blanc-jaunâtre et écailleux ou croûteux recouvrant une zone centrale, ce qui peut indiquer une hyperkératose ou un dépôt sérique/nécrotique. La couleur du fond est principalement marron-noirâtre (hyperpigmentation résiduelle ou hémosidérine) avec des zones rouge-violacées (érythème/inflammation) et des lignes vasculaires fines et ramifiées (télangiectasies). Des petits points noirs et des globules sont également visibles. Les poils émergent et traversent la lésion/cicatrice, ce qui donne un aperçu du contexte cutané environnant. Bien que le diagnostic ne puisse être posé uniquement sur cette image, l'aspect général est compatible avec une cicatrice atrophique ou hypertrophique en cours de guérison/inflammation, ou potentiellement une kératose actinique hyperkératosique ou un carcinome basocellulaire sclérodermiforme/cicatriciel, nécessitant une évaluation clinique et histologique pour la certitude.					
6.<br>L'image dermoscopique montre une lésion cutanée caractérisée par une cicatrice ou une zone de peau altérée. On observe une					

structure linéaire et irrégulière, présentant un mélange de couleurs : des zones blanches et squameuses (écailles ou kératine) sur et autour de la ligne centrale, des zones de pigmentation gris-noirâtre (pouvant indiquer du pigment dans le derme profond ou des croûtes/saignements résolus), et des zones érythémateuses/violacées (rougeurs ou vaisseaux sanguins dilatés). La texture générale est cicatricielle ou fibreuse (aspect blanc-bleuâtre au centre). En périphérie, des poils terminaux sont visibles, encadrant la lésion. Les bords de la zone centrale semblent irréguliers et mal définis.<br>L'ensemble de ces structures (irrégularité des couleurs, des contours, présence de squames et de pigmentation hétérogène dans une zone altérée/cicatricielle) pourrait nécessiter un examen plus approfondi pour exclure des diagnostics tels qu'un carcinome basocellulaire sclérodermiforme, une cicatrice chéloïde, ou d'autres processus inflammatoires ou néoplasiques, mais une interprétation définitive est impossible sans contexte clinique complet et évaluation par un spécialiste					
1. Αυτή η δερματοσκοπική εικόνα, η οποία εστιάζει σε μια ουλή (πιθανότατα μετεγχειρητική), εμφανίζει ένα ακανόνιστο μοτίβο που χαρακτηρίζεται από ποικίλα χρώματα και δομές.<br><br>Παρατηρείται μια επιμήκης, υποκείμενη δερματική βλάβη/ουλή με:<br>Υπολειμματικές τρίχες κατά μήκος και γύρω από αυτήν.<br>Υπολειπόμενη υπέρχρωση (σκούρο καφέ/μαύρο), πιθανόν αιμοσιδηρίνη ή μελαμίνη, εντός της ουλής.<br>Λευκό/κρεμώδες υλικό στην επιφάνεια, το οποίο μπορεί να αντιπροσωπεύει εφελκίδα/κρούστα, έκκριση (π.χ. πύον ή ορό), ή υπολείμματα τοπικής θεραπείας/αλοιφής.<br>Εντός της βλάβης, διακρίνονται λεπτά, ακανόνιστα αγγεία (πιθανώς γραμμικά ή κόμματα) και ερυθρότητα, που υποδηλώνουν φλεγμονή ή επούλωση.					

Τα όρια της βλάβης είναι ακανόνιστα και δυσδιάκριτα λόγω της φλεγμονής και των επιφανειακών υλικών.<br>Η εικόνα δεν είναι τυπική για κοινές μελανωματικές ή μη μελανωματικές δερματικές βλάβες, αλλά δείχνει σημάδια επούλωσης, φλεγμονής και πιθανής δευτερογενούς λοίμωξης ή αντίδρασης σε μια ουλή. Χωρίς κλινικό ιστορικό, οποιαδήποτε διάγνωση είναι αδύνατη, αλλά η εμφάνιση είναι συμβατή με την παρακολούθηση μιας μετεγχειρητικής ουλής ή μιας τραυματικής βλάβης σε διαδικασία επούλωσης					
2. Παρατηρείται μια εικόνα που απεικονίζει μια γραμμική ουλή, πιθανότατα μετεγχειρητική, που βρίσκεται στην περιοχή των φρυδιών (όπως υποδηλώνουν οι τρίχες δεξιά). Η ουλή φαίνεται ατροφική (λεπτή, επίπεδη) και υπερχρωματισμένη (σκουρόχρωμη, καφέ-μαύρη απόχρωση) κατά μήκος του κέντρου της. Υπάρχει λευκωπό υλικό (πιθανόν νεκρωτικό υλικό ή κρούστα) και ερυθρότητα (ερύθημα) κατά μήκος των χειλέων της ουλής, καθώς και διάσπαρτες μικρές μαύρες κηλίδες (πιθανόν ενσφηνωμένες τρίχες ή αιμορραγικές κηλίδες). Τα όρια της ουλώδους περιοχής με τον περιβάλλοντα ιστό είναι σαφή, αλλά το εσωτερικό της φαίνεται ανομοιογενές. Η συνολική εικόνα είναι συμβατή με μια πρόσφατη ή υπό επούλωση ουλή ή με μια δυσχρωμική ουλή, και δεν παρέχει στοιχεία για πρωτοπαθή δερματική βλάβη όπως μελάνωμα ή καρκίνωμα, αλλά δείχνει σημάδια φλεγμονής ή επούλωσης.					
3. Η παρούσα δερματοσκοπική εικόνα απεικονίζει μια γραμμική ουλή με λευκές-λευκογκρίζες, ακανόνιστες δομές που μοιάζουν με κρούστα ή λευκή δαντέλα, διάσπαρτες κατά μήκος της. Η περιοχή της ουλής φαίνεται υπομελαγχρωματική ή ουλική με μια υποκείμενη σκούρα καφέ έως μαύρη, ακανόνιστη μελάγχρωση (πιθανόν μεταφλεγμονώδης ή υπολειπόμενη) και ερυθρές/ιώδεις περιοχές (πιθανή φλεγμονή ή αγγείωση). Στα όρια της βλάβης και εντός αυτής παρατηρείται τριχοφυΐα, ενώ τα όρια φαίνονται σχετικά ασαφή και ακανόνιστα. Λόγω της σαφούς γραμμικής εμφάνισης και των ουλικών χαρακτηριστικών, η εικόνα είναι					

συμβατή με μια ουλή (π.χ., από προηγούμενη βιοψία, τραυματισμό, ή θεραπεία), η οποία μπορεί να εμφανίζει δευτερογενείς αλλαγές όπως φλεγμονή ή υποτροπιάζον/παραμένον δερματικό πρόβλημα. Δεν είναι δυνατή η ακριβής διάγνωση χωρίς κλινική συσχέτιση και ιστορικό, αλλά η γραμμική μορφή υποδηλώνει μια προϋπάρχουσα παρέμβαση ή τραύμα.					
4. Στη δερματοσκοπική εικόνα παρατηρείται μια γραμμοειδής αλλοίωση που φαίνεται να αντιστοιχεί σε μια ουλή ή μια περιοχή με προηγούμενη βλάβη, πιθανώς μετεγχειρητική, λόγω του εμφανή γραμμικού σχήματος και της ατροφίας του δέρματος. Η περιοχή χαρακτηρίζεται από λευκό-γκρίζες έως λευκές, μη δομημένες περιοχές (πιθανώς ίνωση ή ουλές), οι οποίες είναι διάσπαρτες με σκουρότερες, καφέ-μαύρες περιοχές και κηλίδες (πιθανώς μεταφλεγμονώδης υπερμελάγχρωση ή υπολειπόμενο αιματικό ίζημα). Στην περιφέρεια διακρίνονται τριχοθυλάκια (σκούρες τρίχες) και μικρά, κόκκινα, γραμμικά αγγεία (τηλαγγειεκτασίες), ενδεικτικά της διαδικασίας επούλωσης ή φλεγμονής. Τα όρια της αλλοίωσης δεν είναι σαφώς καθορισμένα σε όλη την έκταση, ενώ οι δομές υποδεικνύουν ένα μη ειδικό μοτίβο που σχετίζεται περισσότερο με μια μετατραυματική/μετεγχειρητική αλλοίωση (ουλή) ή μια δερματική πάθηση που επηρεάζει την επούλωση, παρά με μια πρωτογενή μελανωκυτταρική ή μη-μελανωκυτταρική κακοήθεια.					
5. Η εικόνα δείχνει μία βλάβη που φαίνεται να είναι υπολειμματική (ουλές) ή βρίσκεται σε φάση επούλωσης/ανάπλασης. Παρατηρείται μία κεντρική, υπομελανωτική (ανοιχτόχρωμη, σχεδόν λευκή) περιοχή, η οποία διασχίζεται από μία γραμμική, σκούρα καφέ έως μαύρη ζώνη, πιθανώς μία ουλή ή μία περιοχή με εναπόθεση αιμοσιδηρίνης/μελανίνης. Στο κέντρο, πάνω στην ουλή, υπάρχει μία λευκωπή, ινώδης ή κερατίνης επίστρωση (πιθανώς λέπια ή επιφανειακό εξίδρωμα). Τα όρια είναι σχετικά απροσδιόριστα και εξογκωμένα σε ορισμένα σημεία. Επίσης, είναι ορατά ερυθρά, γραμμικά αγγειακά στοιχεία					

(τηλεαγγειεκτασίες) και μαύρες κουκκίδες (πιθανώς θύλακες τριχών, μελανινικές εναποθέσεις ή θρόμβωση). Στην περιφέρεια διακρίνονται τρίχες (πιθανόν από την περιοχή των φρυδιών), ενισχύοντας την πιθανότητα η βλάβη να βρίσκεται σε περιοχή του προσώπου και να σχετίζεται με μία προηγούμενη χειρουργική επέμβαση, τραυματισμό ή φλεγμονώδη διεργασία.					
6. Η δερματοσκοπική εικόνα απεικονίζει μια βλάβη που βρίσκεται πιθανώς στην περιοχή των φρυδιών, όπως υποδεικνύουν οι τριχοθυλακικές δομές στα δεξιά. Παρατηρείται μια ατελώς καθορισμένη, ακανόνιστη βλάβη με ποικιλία χρωμάτων, κυρίως μαύρου, καφέ (φαιού), ερυθρού (ερυθρές γραμμοειδείς δομές που μπορεί να είναι αγγεία), και λευκού (το λευκό-γκρι, ινοειδές υλικό φαίνεται να καλύπτει επιφανειακά τμήματα της βλάβης, δημιουργώντας μια εικόνα λεύκης επιφάνειας ή ουλώδους ιστού). Το επικρατέστερο χρώμα είναι το μαύρο/σκούρο καφέ σε μια διάσπαρτη/ακανόνιστη κατανομή χρωστικής. Υπάρχει μια εμφανής ανωμαλία στα όρια και τη δομή της βλάβης. Η συνολική εμφάνιση, με την ανομοιομορφία των χρωμάτων, την ακανόνιστη κατανομή της χρωστικής, τα ακανόνιστα όρια και την παρουσία λευκών δομών, είναι ύποπτη για κακοήθεια και θα μπορούσε να είναι ενδεικτική ενός μελανώματος σε ουλώδη περιοχή ή ενός βασικοκυτταρικού καρκινώματος με μελαινόμενα στοιχεία και ίνωση/έλκος, αν και η εικόνα δεν είναι απολύτως τυπική. Η άμεση βιοψία και ιστοπαθολογική εξέταση είναι απαραίτητη για την τελική διάγνωση.					
1.<br>Auf diesem dermoskopischen Bild ist eine längliche, lineara Narbenstruktur zu erkennen. Die Narbe selbst zeigt eine weiße, glänzende, strukturlose oder faserige Textur, was auf dermale Fibrose hindeutet. Innerhalb und entlang der Narbe sind Bereiche mit bräunlicher bis schwarzer Pigmentierung (Postinflammatorische Hyperpigmentierung oder Hämosiderinablagerungen) und auch rötliche Areale (Erythem) sichtbar. Am auffälligsten sind die unregelmäßigen					

weißen, krausen, fädigen bis flockigen Strukturen auf der Oberfläche, die möglicherweise Desquamation (Abschuppung) oder Pilzbewuchs (z.B. bei einer Superinfektion) darstellen. Die Narbenbegrenzung erscheint in Teilen etwas diffus und geht in die umgebende Haut über, in der terminale Haarfollikel erkennbar sind. Die sichtbaren dermoskopischen Merkmale deuten primär auf eine Narbenbildung (Cicatrization) hin, wobei die unregelmäßige weiße Oberflächenstruktur und das Erythem eine aktive Phase oder eine mögliche sekundäre Infektion/Inflammation suggerieren.					
2. Das dermatoskopische Bild zeigt eine narbenartige Läsion mit mehreren Strukturen. Im Zentrum der Läsion ist eine weißliche, netzartige oder schuppige Textur sichtbar, die möglicherweise auf eine Hyperkeratose oder Narbengewebe hindeutet. Die Hauptfarbe der Läsion selbst ist ein unregelmäßiges, dunkelbraun-schwärzliches bis leicht rötlich-violettes Areal, was auf die Anwesenheit von Pigment und Gefäßen (rötliche Bereiche und fein erweiterte Gefäße) schließen lässt. Die Grenzen der Läsion scheinen insgesamt unscharf und unregelmäßig zu sein. Ein Teil der Läsion zeigt eine Regression oder Narbenbildung. Im umgebenden Bereich sind Haare und normale Haut zu erkennen. Die Kombination aus unregelmäßigem Pigment, Regression/Vernarbung, und der unklaren Begrenzung erfordert eine dermatologische Abklärung, um eine mögliche Diagnose wie ein Melanom (insbesondere ein auf Narbengewebe entstandenes oder regrediertes) oder eine andere pigmentierte oder nicht-pigmentierte Hautneoplasie sicher ausschließen zu können. Die netzartigen, weißlichen Strukturen könnten auch Pilzmyzelien bei einer sekundären Infektion darstellen, was aber in diesem Kontext unwahrscheinlich ist.					
3. Auf dem dermoskopischen Bild ist eine lineare Läsion zu erkennen, die wahrscheinlich einer (älteren) Narbe entspricht, welche möglicherweise durch eine sekundäre entzündliche Reaktion modifiziert ist.					

Farben: Die Läsion zeigt eine unregelmäßige, schmutzig-bräunlich-schwärzliche Pigmentierung (wahrscheinlich postinflammatorische Hyperpigmentierung oder in der Narbe gelegenes Pigment). Daneben sind rötliche Bereiche (Erythem/Entzündung) und weißliche, strukturlose Areale zu sehen, die auf Fibrose/Narbenbildung und/oder Entzündungsaktivität hinweisen.<br>Muster: Auffällig sind weißliche, lamelläre (schuppige) Strukturen auf der Oberfläche, die besonders im zentralen Bereich der Narbe konzentriert sind, sowie einige feine, bräunlich-schwarze Punkte an der Peripherie der zentralen Pigmentierung. Es sind lineare, leicht gewundene oder verzweigte Gefäße im Bereich der Rötung sichtbar, was auf eine Entzündung hindeutet. Die gesamte Struktur wirkt asymmetrisch und unstrukturiert. Die Haarfollikel (mit Haarschäften) sind an den Rändern der Narbe erkennbar.<br>Grenzen: Die Grenze der braunen Pigmentierung ist unscharf und unregelmäßig.<br>Mögliche Diagnose: Das Bild deutet auf eine aktive entzündliche Dermatose auf dem Boden einer Narbe oder einer chronischen Läsion hin. Die Kombination aus weißlichen Schuppen, Pigmentierung und entzündlichen Gefäßen auf einer Narbenplatte kann an einen Lichen planus auf der Narbe (Köbner-Phänomen), eine aktinische Keratose auf Narbenboden oder eine andere entzündliche, lichenoide Reaktion erinnern. Eine sich entwickelnde Malignität (z. B. amelanotisches oder pigmentiertes Karzinom) kann aufgrund der unstrukturierten und polymorphen Merkmale nicht ausgeschlossen werden. Zur definitiven Diagnose ist eine histopathologische Untersuchung erforderlich.					
4. Auf dem dermoskopischen Bild ist eine ältere Narbe im Bereich der Augenbraue oder des behaarten Kopfes zu erkennen, was durch die Hypopigmentierung (blassere, weißliche Farbe) und das Fehlen normaler Hautmuster (Grenzverlust) im zentralen Bereich der Läsion suggeriert wird.					

Farben und Muster: Die zentrale Narbenfläche zeigt eine weißlich-gelbliche Verfärbung, was typisch für Narbengewebe und Fibrose ist. Es sind feine, weiße, fädige Strukturen und schuppenartige oder krustige Auflagerungen zu sehen, die auf eine Flechten-artige (lichenoide) oder entzündliche Aktivität an den Rändern oder in Teilen der Narbe hindeuten könnten (möglicherweise im Zusammenhang mit einer Behandlung oder Reizung).<br>Gefäße: Es sind punktförmige und/oder kommaförmige rötliche Gefäße erkennbar, die auf eine Hypervaskularisierung hinweisen und typisch für aktive, möglicherweise hypertrophe Narben oder Keloide sein können, insbesondere wenn sie von Rötung begleitet sind. Dunkle Bereiche können postinflammatorische Hyperpigmentierung darstellen.<br>Grenzen: Die Narbe ist relativ scharf begrenzt zum umgebenden behaarten Bereich. Es ist zu beachten, dass im angrenzenden Bereich Haare (Augenbrauen oder Kopfhaar) vorhanden sind, die in die Narbe hineinwachsen oder an ihrem Rand enden.<br>Mögliche Diagnose: Das Bild deutet am wahrscheinlichsten auf eine Narbenbildung (z. B. eine ältere, möglicherweise hypertrophe Narbe) mit sekundärer, entzündlicher/lichenoider Aktivität oder Krustenbildung hin. Bei der Bewertung einer Narbe sind immer die klinischen Merkmale (z.B. Erhabenheit, Juckreiz) zu berücksichtigen.					
5. Auf diesem dermoskopischen Bild ist eine lineare Läsion zu erkennen, die am wahrscheinlichsten eine Narbe darstellt.<br>Farben und Strukturen: Auffällig sind die weißen, narbenartigen, faserigen oder streifigen Strukturen, die sich entlang der Läsion erstrecken und auf eine Kollagenreorganisation in der Dermis hindeuten. Darüber hinaus sind braune bis schwärzliche Verfärbungen vorhanden, die möglicherweise auf eine postinflammatorische Hyperpigmentierung oder eine Verunreinigung/Tätowierung durch die ursprüngliche Verletzung (wie eine Schmutzeinsprengung) zurückzuführen sind.					

Vaskuläre Muster: Es sind feine, rötliche Linien (Teleangiektasien) sichtbar, die auf eine erhöhte Vaskularisierung im Heilungsgewebe hinweisen können.<br>Grenzen und Umgebung: Die Grenzen sind unscharf zum umgebenden Gewebe hin, das Haare zeigt, was die Läsion in einer behaarten Region lokalisiert (z. B. Augenbraue oder Kopfhaut).<br>Mögliche Diagnose: Angesichts der weißen fibrotischen Bänder und der linearen Form ist die wahrscheinlichste Diagnose eine atrophe oder hypertrophe Narbe, eventuell mit traumatischer Pigmentierung oder postinflammatorischer Hyperpigmentierung.					
6. Auf dem dermoskopischen Bild ist eine Narbenstruktur erkennbar, die durch das Fehlen von Haarfollikeln in weiten Teilen der zentralen Läsion und eine blass-rosa bis weißliche Grundstruktur mit sichtbaren, teils unregelmäßigen linearen Gefäßen (Teleangiektasien) gekennzeichnet ist. Über dieser Narbenbasis zeigen sich unregelmäßige, netz- oder sternförmige, weißliche, glänzende Strukturen, die an Pseudo-Wickham-Streifen oder eine Oberflächenfibrose erinnern, begleitet von weißlichen, krustigen Auflagerungen/Schuppen und dunkelbraunen bis schwärzlichen Pigmentpunkten (vermutlich postinflammatorische Pigmentierung oder Pigmentstippen). Die Begrenzung der Läsion ist unregelmäßig und unscharf. Die Kombination der weißlichen, netzartigen Strukturen auf einer Narbe mit entzündlichen und pigmentierten Elementen legt differentialdiagnostisch eine entzündliche Dermatose auf Narbengewebe nahe, möglicherweise einen Lichen planus (Knötchenflechte) oder eine lichenoide Reaktion.					
